# Global Incidence, Risk Factors, and Trends of Pharyngeal Cancer by Anatomical Sites: A Systematic Analysis of Cancer Registries

**DOI:** 10.1002/cnr2.70590

**Published:** 2026-07-15

**Authors:** Junjie Huang, Sze Chai Chan, Wing Sze Pang, Yat Ching Fung, Shui Hang Chow, Veeleah Lok, Lin Zhang, Xu Lin, Don Eliseo Lucero‐Prisno, Wanghong Xu, Zhi‐Jie Zheng, Edmar Elcarte, Mellissa Withers, Claire Chenwen Zhong, Martin C. S. Wong, NCD Global Health Research Group, Association of Pacific Rim Universities (APRU)

**Affiliations:** ^1^ The Jockey Club School of Public Health and Primary Care, Faculty of Medicine Chinese University of Hong Kong Hong Kong SAR China; ^2^ Centre for Health Education and Health Promotion, Faculty of Medicine The Chinese University of Hong Kong Hong Kong SAR China; ^3^ Department of Global Public Health, Karolinska Institute Karolinska University Hospital Stockholm Sweden; ^4^ The School of Public Health and Preventive Medicine Monash University Melbourne Victoria Australia; ^5^ Department of Thoracic Surgery, The First Affiliated Hospital, School of Medicine Zhejiang University Hangzhou Zhejiang China; ^6^ Department of Global Health and Development London School of Hygiene and Tropical Medicine London UK; ^7^ School of Public Health Fudan University Shanghai China; ^8^ Department of Global Health, School of Public Health Peking University Beijing China; ^9^ College of Nursing University of the Philippines Manila Philippines; ^10^ Department of Population and Health Sciences, Institute for Global Health University of Southern California Los Angeles USA; ^11^ School of Public Health The Chinese Academy of Medical Sciences and Peking Union Medical College Beijing China

**Keywords:** incidence, pharyngeal cancer, risk factors, temporal trend

## Abstract

**Purpose:**

Nasopharyngeal, oropharyngeal, and hypopharyngeal cancers are the three types of pharyngeal malignancies that vary in prognosis. This study aims to evaluate the global disease incidence, risk factors, and trends of pharyngeal cancer by anatomical sites.

**Methods:**

The *Global Cancer Observatory, Global Burden of Disease, and Cancer Incidence in Five Continents Plus*, were accessed to investigate the disease burden and related risk factors by anatomical site. Average Annual Percentage Changes were computed using Joinpoint regression to assess trends in age‐standardized rates (ASR) of incidence of pharyngeal cancer.

**Results:**

An estimated total of 316,020 (ASR=3.5) pharyngeal cancer cases were reported in 2020, with nasopharyngeal cancer being the most prevalent subtype (1.7), followed by oropharyngeal (1.1) and hypopharyngeal cancer (0.91). Nasopharyngeal, oropharyngeal, and hypopharyngeal cancers were most common in South‐Eastern Asia (ASR = 5.0), Western Europe (ASR = 2.8), and South‐Central Asia (ASR = 2.1), respectively. Higher Human Development Index (HDI), smoking, alcohol drinking, unhealthy dietary habits, hypertension, and lipid disorders were associated with higher pharyngeal cancer incidence. The results indicated a declining or constant trend in hypopharyngeal and nasopharyngeal cancers and a notable rise in oropharyngeal cancer.

**Conclusion:**

The incidence of pharyngeal cancer subtypes demonstrates significant geographical heterogeneity. While hypopharyngeal and nasopharyngeal cancers are declining or stable, oropharyngeal cancer is on a clear upward trajectory. Given the prolonged latency between risk factor exposure and clinical onset, the oropharyngeal cancer burden is projected to rise further.

**Abbreviations**
β: beta coefficient
AAPC: average annual percentage change
ASR: age‐standardized rate
CI5 Plus: Cancer Incidence in Five Continents Plus
CIs: confidence intervals
CrI: credible interval
DM: diabetes mellitus
EBV: Epstein‐Barr Virus
GBD: Global Burden of Disease
GDP: gross domestic product
GLOBOCAN: Global Cancer Observatory
HDI: human development index
HNC: head and neck cancer
HPV: human papillomavirus
IARC: International Agency for Research on Cancer
ICD: International Classification of Diseases
OR: odds ratio
RR: relative risk
SEER: surveillance, epidemiology, and end results
UN: United Nations

## 1 Introduction

Pharyngeal cancer, including nasopharyngeal cancer, oropharyngeal cancer, and hypopharyngeal cancer [1, 2, 3, 4], has a continuous increasing trend worldwide, especially among developed countries [5]. Between 2012–2018, the overall five‐year survival rate for this was 68% [6], a figure that remains low due to the difficulty of early detection and the frequency of advanced‐stage diagnosis, with prognosis varying by anatomical site [5, 7]. Hence, it is necessary for each country to develop targeted detection strategies tailored to the epidemiology of pharyngeal cancer subtypes.

The use of tobacco, alcohol consumption, human papillomavirus (HPV), and Epstein‐Barr virus (EBV) infections are the common risk factors for pharyngeal cancer [2]. Western countries have also discovered a correlation between pharyngeal cancer and oral sex [2]. Occupational exposure to environmental risk factors also increases the risk of pharyngeal cancer [8, 9]. Unhealthy dietary habits and poor hygiene are also found associated with pharyngeal cancer, although more evidence is needed [10, 11, 12]. Nevertheless, risk factors for pharyngeal cancer could vary by anatomical sites.

Previous studies had examined the epidemiology of pharyngeal cancer. However, few studies investigated its subtypes, and they were limited to reporting for either a single country or the typical geographical location [2, 13, 14, 15, 16, 17]. Hence, this study utilized the up‐to‐date and high‐quality data from international and national cancer registries to examine the burden of pharyngeal cancer across different anatomical sites. The novelty of this study was focused on investigating the incidence, associated risk factors, and trend of pharyngeal cancer by subtype. These findings underscore the urgent need for subtype‐specific prevention and early detection strategies tailored to distinct epidemiological profiles across regions.

## 2 Results

### 2.1 Pharyngeal Cancer Incidence in 2020 Overall and by Subtype

**FIGURE 1:**
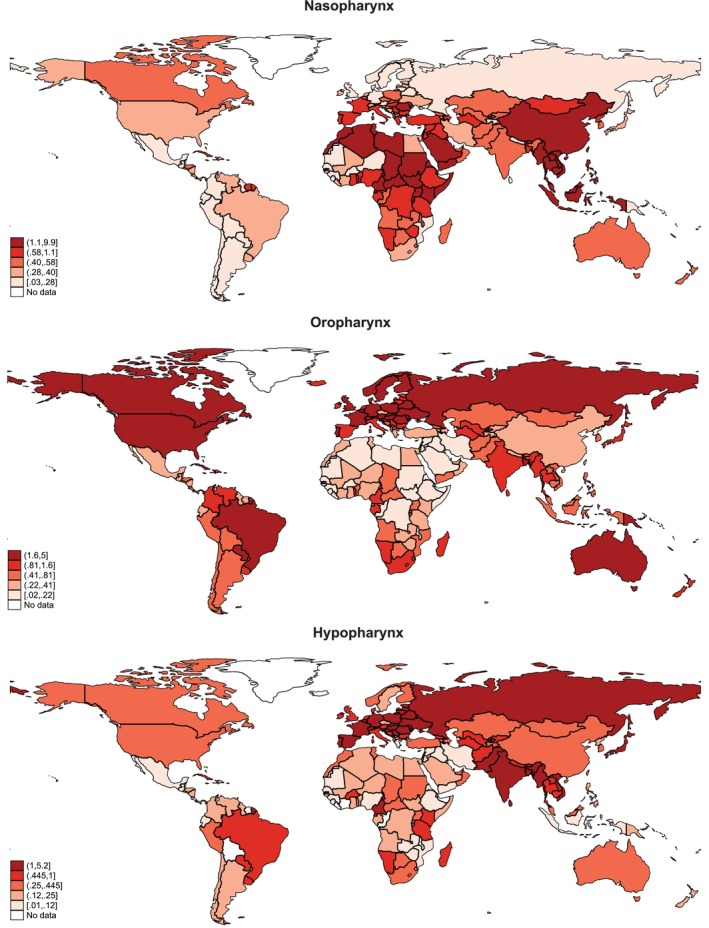
Global age‐standardized incidence of pharyngeal cancer by anatomical sites, in 2020.

An estimated total of 316 020 (age‐standardized rate (ASR) = 3.5) pharyngeal cancer cases were reported in 2020, with nasopharyngeal cancer being the most prevalent subtype (1.7), followed by oropharyngeal (1.1) and hypopharyngeal cancer (0.91) (Figure 1). South‐Eastern Asia (6.4), Western Europe (4.8), and Central and Eastern Europe (4.6) reported the highest ASR, with a 12‐fold difference observed. As for country differences, Brunei Darussalam (9.9), Bangladesh (8.4), and Viet Nam (7.9) reported the highest ASR. There was a geographical disparity in the incidence of pharyngeal cancer by subtype. South‐Eastern Asia (5.0), Micronesia (3.3), and Eastern Europe (2.7) had the highest incidence of nasopharyngeal cancer. For oropharyngeal cancer, a higher incidence was observed in Western Europe (2.8), Northern Europe (2.6) and Northern America (2.4); a higher incidence of hypopharyngeal cancer was found in South‐Central Asia (2.1) and Central and Eastern Europe (1.9).

### 2.2 Pharyngeal Cancer Incidence by Subgroup in 2020

The male incidence of pharyngeal cancer was 245 670 across the world (ASR = 5.6), which was much higher than the female incidence (70 350 cases, ASR = 1.5). The highest incidence in males was reported in South‐Eastern Asia (10.1 vs. 2.9 in females), Central and Eastern Europe (9.0 vs. 1.2), and Western Europe (7.4 vs. 2.3). As for countries, the highest incidence in males was reported in Romania (13.9 vs. 1.8 in females), Bangladesh (13.8 vs. 2.7), and Belarus (13.8 vs. 0.46). By comparing the young and old populations, a higher incidence was reported in the old populations with 202 306 cases and an ASR of 12.6 (young: 78 608, ASR: 1.9). The highest incidence in the old was reported in South‐Eastern Asia (20.9 vs. 4.3 in young), Western Europe (20.8 vs. 1.5), and Central and Eastern Europe (19.1 vs. 2.0). As for countries, the highest incidence in the old was reported in Brunei Darussalam (37.0 vs. 4.5 in the young), France (32.8 vs. 2.2), and Bangladesh (32.1 vs. 4.2).

### 2.3 Associations of Risk Factors With Pharyngeal Cancer Incidence

In all sexes and ages, pharyngeal cancer incidence was associated with higher human development index (HDI) (beta coefficient (β) = 0.270, 95% confidence interval (CI): 0.084 to 0.457, *p* = 0.005), smoking (β = 0.115, 95% CI: 0.066 to 0.164, *p* < 0.001), alcohol drinking (β = 0.100, 95% CI: 0.047 to 0.154, *p* < 0.001), unhealthy dietary habits (β = 0.054, 95% CI: 0.029 to 0.079, *p* < 0.001), hypertension (β = 0.046, 95% CI: 0.012 to 0.079, *p* = 0.008), and lipid disorder (β = 0.044, 95% CI: 0.021 to 0.068, *p* < 0.001) (Table S1). Similar associations were found in the male and old populations. In the female and young populations, pharyngeal cancer incidence was associated with unhealthy dietary habits (Female: β = 0.022, 95% CI: 0.008 to 0.037, *p* = 0.003; Young: β = 0.018, 95% CI: 0.002 to 0.034, *p* = 0.027) and lower prevalence of obesity (Female: β = –0.022, 95% CI: –0.033 to –0.010, *p* < 0.001; Young: β = –0.021, 95% CI: −0.037 to –0.005, *p* = 0.011).

### 2.4 Associations of Risk Factors With Pharyngeal Cancer Incidence by Subtype

**FIGURE 2:**
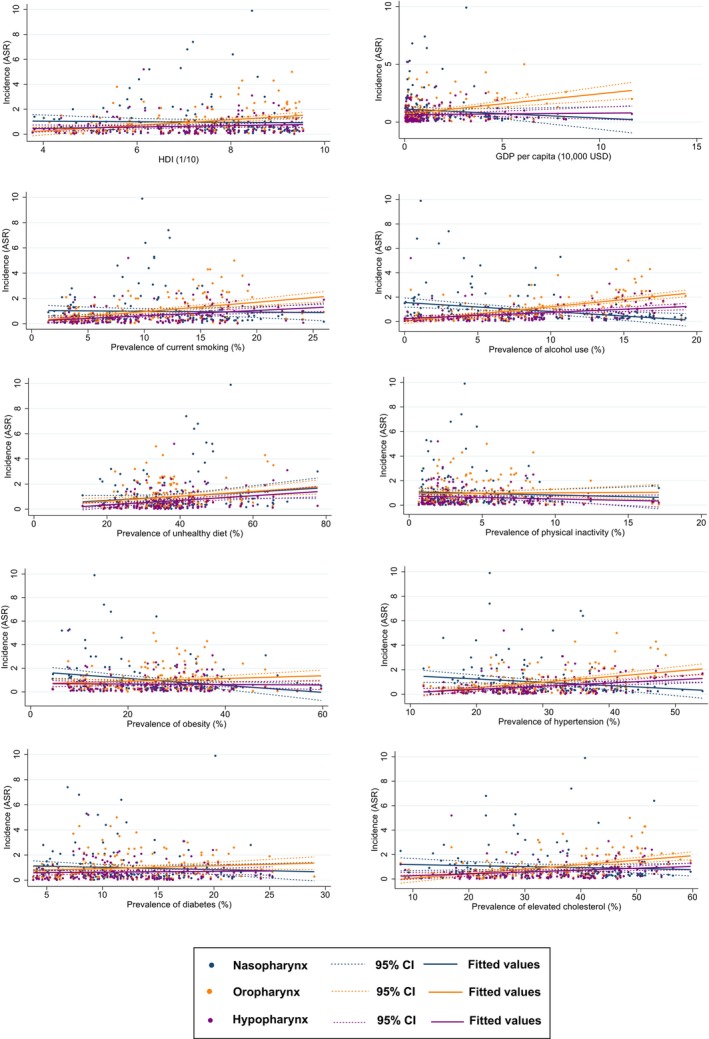
Associations of risk factors with pharyngeal cancer incidence by anatomical sites.

As for the subtypes, a distinctive association was observed in nasopharyngeal cancer; the nasopharyngeal cancer incidence was associated with a lower prevalence of alcohol drinking (β = –0.074, 95% CI: –0.114 to –0.034, *p* < 0.001), obesity (β = –0.030, 95% CI: –0.049 to –0.011, *p* = 0.003), and hypertension (β = –0.027, 95% CI: –0.052 to –0.002, *p* = 0.036) (Table S1) (Figure 2).

The oropharyngeal cancer was associated with higher HDI (β = 0.226, 95% CI: 0.133 to 0.318, *p* < 0.001), gross domestic product (GDP) per capita (β = 0.174, 95% CI: 0.106 to 0.242, *p* < 0.001), higher prevalence of smoking (β = 0.074, 95% CI: 0.050 to 0.098, *p* < 0.001), alcohol drinking (β = 0.118, 95% CI: 0.095 to 0.140, *p* < 0.001), unhealthy dietary habits (β = 0.019, 95% CI: 0.006 to 0.032, *p* = 0.004), hypertension (β = 0.045, 95% CI: 0.029 to 0.061, *p* < 0.001), and lipid disorder (β = 0.037, 95% CI: 0.025 to 0.048, *p* < 0.001). In the old population, diabetes (β = –0.067, 95% CI: –0.134 to –0.001, *p* = 0.048) was negatively associated with the oropharyngeal cancer incidence (Table S1).

The hypopharyngeal cancer incidence was associated with a higher prevalence of smoking (β = 0.042, 95% CI: 0.022 to 0.062, *p* < 0.001), alcohol drinking (β = 0.051, 95% CI: 0.031 to 0.072, *p* < 0.001), unhealthy dietary habits (β = 0.018, 95% CI: 0.008 to 0.028, *p* < 0.001), hypertension (β = 0.026, 95% CI: 0.013 to 0.039, *p* < 0.001), and lipid disorder (β = 0.015, 95% CI: 0.005 to 0.025, *p* = 0.003). In the older population, diabetes (β = –0.071, 95% CI: –0.130 to –0.012, *p* = 0.018) was negatively associated with hypopharyngeal cancer incidence (Table S1).

### 2.5 Trend Analysis of Pharyngeal Cancer Incidence

The overall incidence trend of pharyngeal cancer was increasing, in which 14 countries reported increasing trends and no country reported a decreasing trend (Table S2, Figures S1 and S2). The most notable increase was found in Cyprus (Average Annual Percentage Change (AAPC): 24.42, CI: 10.88 to 39.61, *p* = 0.002), followed by the United Kingdom (AAPC: 7.60, CI: 6.46 to 8.75, *p* < 0.001), and Estonia (AAPC: 7.17, CI 2.24 to 12.34, *p* = 0.010). Considering the male and female subgroups, the incidence trends were both increasing (Male: 14 increases and 3 decreases; Female: 15 increases and 1 decrease). The increasing trend was more evident in females than males. Notable increases for females were found in Cyprus (AAPC: 37.92, CI: 13.64 to 67.38, *p* = 0.005), Estonia (AAPC: 23.21, CI: 8.53 to 39.88, *p* = 0.001), and Japan (AAPC: 9.10, CI: 2.21 to 16.45, *p* = 0.015). Similarly, the incidence trends of pharyngeal cancer in the young and old populations were increasing (Young: 13 increasing and 0 decreasing; Old: 13 increasing and 0 decreasing); the increase in the old population was slightly higher than the young population. Notable increases for the old population were found in Cyprus (AAPC: 26.93, CI: 13.52 to 41.93, *p* = 0.001), the United Kingdom (AAPC: 7.85, CI: 6.85 to 8.85, *p* < 0.001), and Denmark (AAPC: 7.16, CI: 5.76 to 8.59, *p* < 0.001). The other 28 countries did not show significant increases or decreases during the period.

### 2.6 Age‐ and Sex‐Specific Trend Analysis by Subtype

**FIGURE 3:**
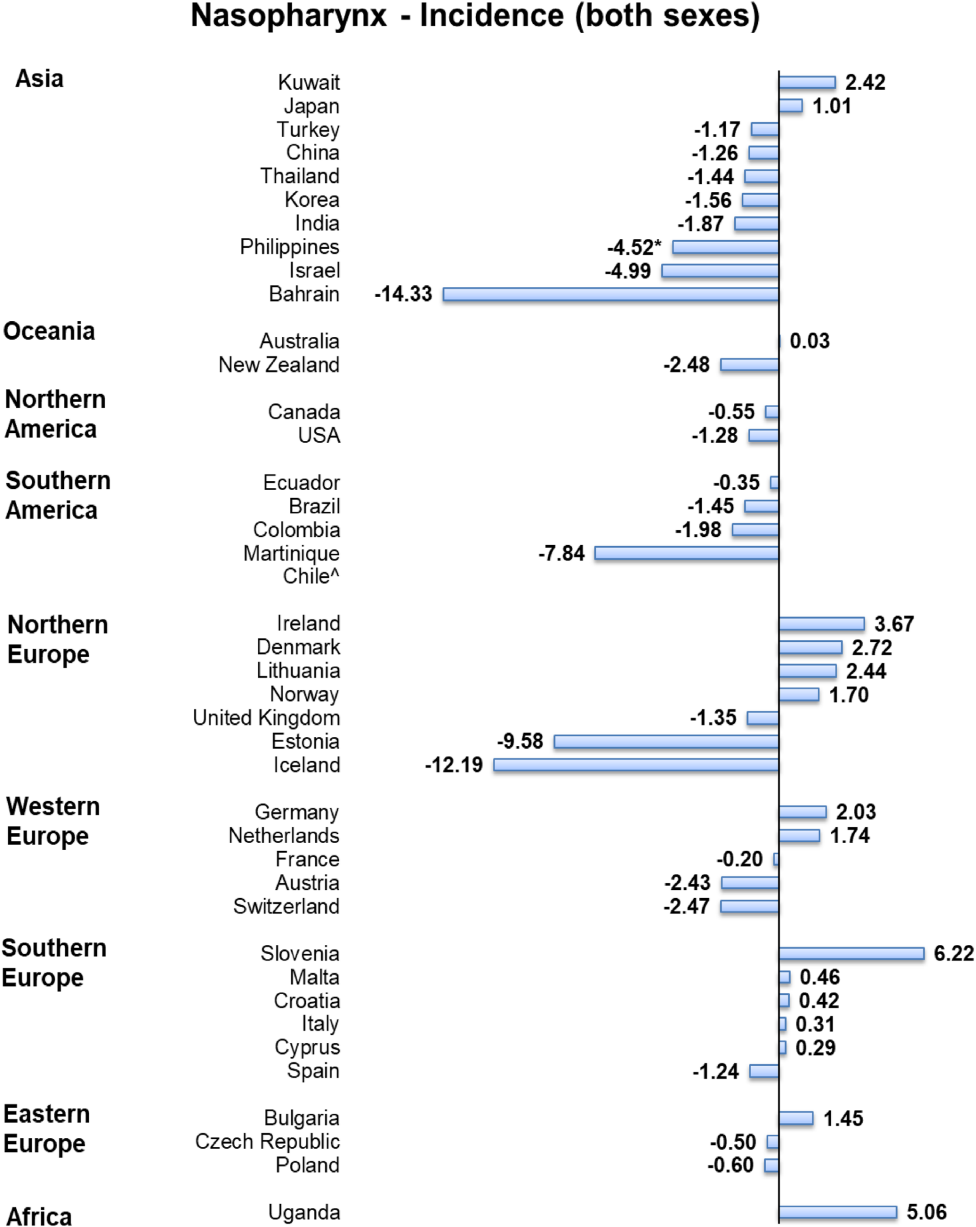
Average Annual Percentage Change (AAPC) of nasopharyngeal cancer incidence.

Comparing the three subtypes, the increasing trend of oropharyngeal cancer was more significant than the other two. In terms of nasopharyngeal cancer (Figure 3), the incidence trend was found significant in the Philippines only with a decreasing trend (AAPC: −4.52, CI: −6.43 to −2.58, *p* = 0.001). The other 39 countries did not show significant increases or decreases during the period. In male and female populations, no increasing trend was reported, and the decreasing trends were more pronounced among the female population. Three countries reported decreasing trends in females, including Iceland (AAPC: −20.55, CI: −24.97 to −15.87, *p* < 0.001), Bahrain (AAPC: −15.50, CI: −24.76 to −5.11, *p* = 0.010), and China (AAPC: −3.89, CI: −6.07 to −1.65, *p* = 0.001). However, the younger population reported more significant increasing trends compared with the older counterparts (young: 3 increases and 3 decreases; old: 1 increase and 5 decreases). Norway reported the highest rise (AAPC: 21.62, CI: 3.93 to 42.31, *p* = 0.021), followed by Slovenia (AAPC: 19.46, CI: 11.56 to 27.91, *p* < 0.001), and Ecuador (AAPC: 5.00, CI: 2.77 to 7.29, *p* < 0.001) (Table S2).

**FIGURE 4:**
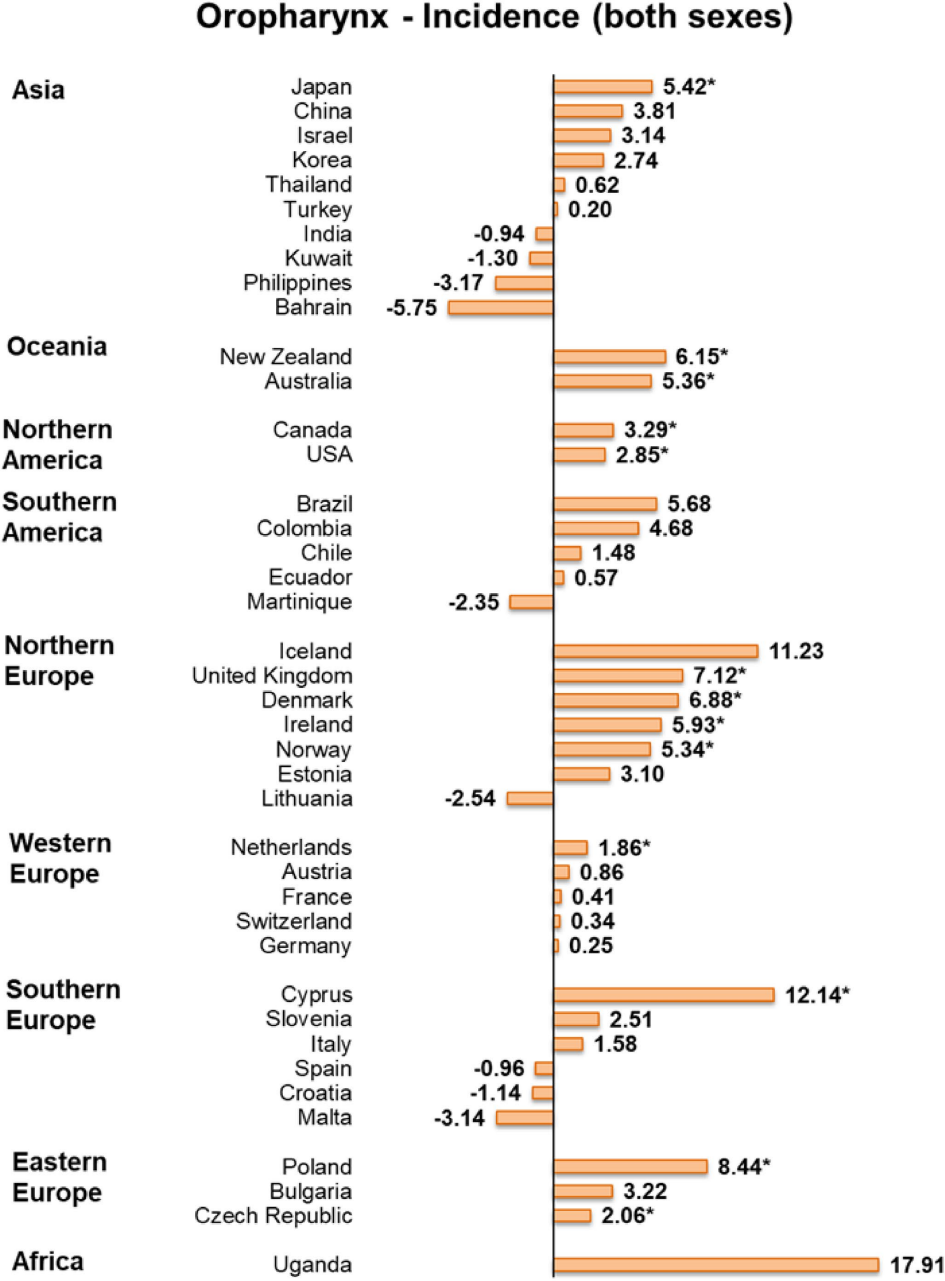
Average Annual Percentage Change (AAPC) of oropharyngeal cancer incidence.

A total of 13 countries showed increasing trends, and no country showed a decreasing trend in oropharyngeal cancer (Figure 4). The significant rises were found in Cyprus (AAPC: 12.14, CI: 5.13 to 19.62, *p* = 0.003), Poland (AAPC: 8.44, CI: 0.35 to 17.20, *p* = 0.043), and the United Kingdom (AAPC: 7.12, CI: 6.11 to 8.15, *p* < 0.001). The other 28 countries did not show significant increases or decreases during the period. Regarding the two sexes, the rising trend was more pronounced in females than males (Male: 11 increases and 1 decrease; Female: 14 increases and 0 decreases). The significant increases in females were found in Martinique (AAPC: 31.13, CI: 17.28 to 46.61, *p* = 0.001), Estonia (AAPC: 31.04, CI: 11.66 to 53.78, *p* = 0.001), and Bahrain (AAPC: 29.84, CI: 25.74 to 34.06, *p* < 0.001). Regarding the age group, 15 countries reported increasing trends, and 1 country reported a decreasing trend in the old population; the number was much higher than in the young population (6 increases and 6 decreases). The significant increases in the old were found in Cyprus (AAPC: 16.45, CI: 8.55 to 24.92, *p* = 0.001), Poland (AAPC: 9.62, CI: 0.47 to 19.61, *p* = 0.041), and Denmark (AAPC: 8.31, CI: 6.74 to 9.91, *p* < 0.001) (Table S2).

**FIGURE 5:**
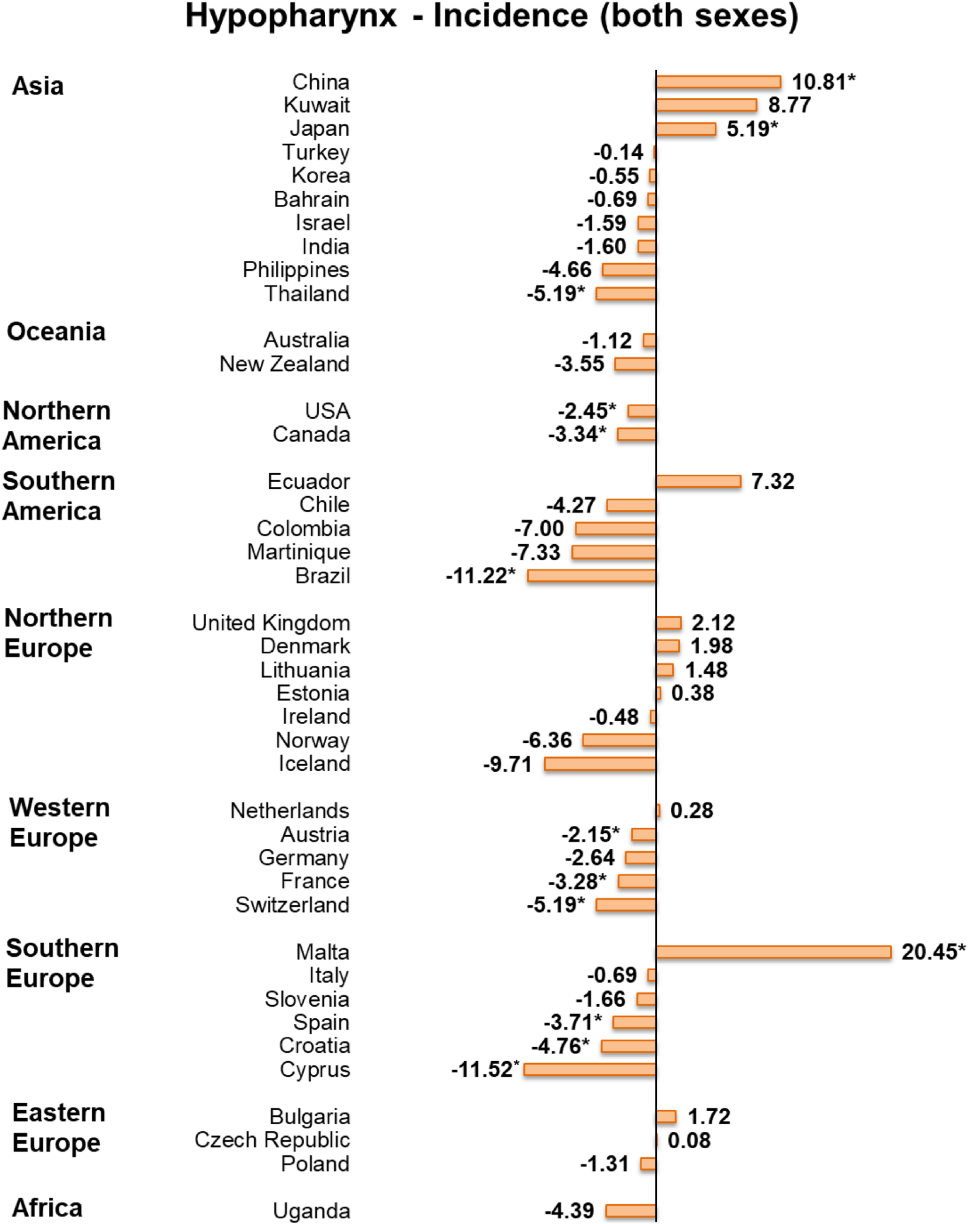
Average Annual Percentage Change (AAPC) of hypopharyngeal cancer incidence.

As for hypopharyngeal cancer, the overall incidence trend was decreasing, with three countries having increasing trends and nine countries having decreasing trends (Figure 5). Notable decreases were found in Brazil (AAPC: −11.22, CI: −18.69 to −3.06, *p* = 0.014), Switzerland (AAPC: −5.19, CI: −8.77 to −1.45, *p* = 0.013), and Thailand (AAPC: −5.19, CI: −9.33 to −0.86, *p* = 0.025). The other 29 countries did not show significant increases or decreases during the period. However, the incidence trend of the female population was generally increasing (female: 5 increases and 4 decreases; male: 3 increases and 14 decreases). Notable increases in the female population were found in Malta (AAPC: 33.26, CI: 21.74 to 45.87, *p* < 0.001), Bahrain (AAPC: 29.34, CI: 24.50 to 34.37, *p* < 0.001), and Estonia (AAPC: 19.09, CI: 6.83 to 32.76, *p* = 0.002). In addition, the incidence trends of the two age groups were decreasing (young: 2 increases and 10 decreases; old: 6 increases and 10 decreases). Notable increases in the old population were found in Malta (AAPC: 19.05, CI: 6.54 to 33.03, *p* = 0.007), China (AAPC: 13.97, CI: 9.07 to 19.09, *p* < 0.001), and Kuwait (AAPC: 12.5, CI: 4.2 to 21.46, *p* = 0.003) (Table S2).

## 3 Discussion

### 3.1 Summary of Major Findings

This study is a holistic analysis of the global disease burden of, risk factors associated with, and temporal trends of pharyngeal cancer by subtype. Several major findings were discovered from the study. (1) In terms of geographical variations, a higher ASR of pharyngeal cancer overall was observed in some regions sporadically, including Polynesia, Northern America, and Eastern Asia, while the incidence of pharyngeal cancer was lower in lower‐income countries, mostly in the African region. The highest incidence of oropharyngeal, hypopharyngeal, and nasopharyngeal cancer was found in Western Europe, South‐Central Asia, and South‐Eastern Asia, respectively. (2) The incidence of pharyngeal cancer in males was remarkably higher than in females, and such a phenomenon was consistent throughout the regions observed. The older population was also found to have higher cancer incidence compared to the younger population. (3) Pharyngeal cancer incidence was associated with higher HDI, smoking, alcohol drinking, unhealthy dietary habits, hypertension, and lipid disorders. (4) There were significant disparities among the three subtypes of pharyngeal cancer: a significant increasing trend was observed for incidence of pharyngeal cancer overall and oropharyngeal cancer, while a decreasing and a stable trend were observed for hypopharyngeal and nasopharyngeal cancers, respectively.

### 3.2 Variation in the Disease Burden

A higher disease burden of pharyngeal cancer was found in various regions sporadically distributed throughout the world. This might be attributable to the great variation in the incidence by subtype, possibly due to the difference in prevalence of some major risk factors in the respective regions, including the prevalence of tobacco smoking and HPV (oropharyngeal cancer) [18], malnutrition (hypopharyngeal cancer) [19], consumption of salt‐preserved fish and EBV (nasopharyngeal cancer) [20]. On the contrary, the lower disease burden in Africa might be due to limited manpower, which may limit diagnostic capacity [21]. A significant disparity was found between the disease burden among the two sexes, with males having an evidently higher incidence, which was likely due to the higher prevalence of tobacco use in males (36.7%; 7.8% in females) [22]. Also, the HPV vaccination coverage for males was relatively lower than females regardless of region. Additionally, disparities between high‐income and low‐income countries may also substantially shape the pharyngeal cancer burden in local regions. In low‐income countries, healthcare gaps restrict access to screening and specialized oncology care, leading to underdiagnosis [23]. These same gaps are reflected in the absence of high‐quality cancer registries, which necessitates data imputation in global data sources and may further compound the underestimation of disease burden [23]. Betel nut consumption is predominantly concentrated in low‐ and middle‐income countries, where it accounts for 90.2% of the global attributable oral cancer cases [24]. However, the proportion of pharyngeal cancer attributable to betel nut remains understudied, despite evidence suggesting an increased risk [25]. The association between pharyngeal cancer and betel nut consumption may help explain why Polynesia and Eastern Asia, despite not ranking among the highest HDI regions, demonstrated the highest incidence of pharyngeal cancer, coinciding with their high rates of betel nut consumption [24]. In contrast, Northern America also has a high incidence of pharyngeal cancer, yet is characterized by a high HDI. In this context, the elevated incidence may be more attributable to hypertension, and lipid disorders, non‐communicable diseases commonly existed in high HDI regions [26]. However, these non‐communicable diseases may increasingly contribute to pharyngeal cancer burden in low HDI countries as they undergo epidemiological transition [26]. However, further evidence is needed to establish the relationship between the factors discussed above and the regional variation of pharyngeal cancer.

### 3.3 Risk Factors Associated With Pharyngeal Cancer

In this study, smoking was found to be significantly associated with pharyngeal cancer incidence. Previous studies have shown that smoking can act as an individual risk factor for oropharyngeal cancer, and the effect was even stronger in HPV‐positive patients (odds ratio (OR) = 355.82, 95% credible interval (CrI) = 177.00–715.30) [27]. Alcohol is another associated risk factor with pharyngeal cancer in the current study, consistent with previous studies showing an increased risk of oral and pharyngeal cancer among heavy alcohol drinkers (relative risk (RR) = 5.24, 95% CI = 4.36–6.30) [28] and among never smokers consuming < 3 drinks/day compared with non‐drinkers (OR = 3.4, 95% CI = 1.1–10.1) [29]. Notably, a synergistic effect of alcohol and tobacco (OR = 5.37, 95% CI = 3.54–8.14) was found on the risk of oropharyngeal cancer [30]. This might relate to the cytotoxicity of alcohol on cells, which enhances the chance of damaged cells inhaling genotoxic substances from tobacco [31].

HPV prevalence was positively associated with pharyngeal cancer, particularly oropharyngeal cancer, with the mean pooled prevalence rising from 32.3% (95% CI = 23.7%–40.7%, *p* < 0.001) in the pre‑1995 period to 52.9% (95% CI = 42.8%–63.0%, *p* < 0.001) between 2005 and 2014 worldwide [32]. The link was further supported by a case‐control study on the serums collected from Norway, Finland, and Sweden, in which the presence of HPV 16 was associated with head and neck carcinoma, including pharyngeal cancer [33].

Unhealthy diets were found to be associated with pharyngeal cancer incidence, which was in line with previous studies [20]. For instance, higher intake of fried food was associated with pharyngeal cancer (OR = 1.10, 95% CI = 1.04–1.17) [34], while the consumption of salt‐preserved meat and fish was discovered as a risk factor in specific regions such as China, with an OR of 3.56 (95% CI = 1.89–6.72) for monthly intake among men in Shanghai [20, 35].

On a population level, our analyses relied on national and international prevalence data to quantify risk factors such as hypertension, lipid disorders, and diabetes mellitus (DM), and their association with pharyngeal cancer incidence. This ecological research demonstrated a significant association between pharyngeal cancer incidence and hypertension and lipid disorder, but not DM. These findings are consistent with prior evidence suggesting that metabolic syndrome–related morbidities were associated with increased risk of pharyngeal cancer [36, 37]. On the other hand, the lack of association between diabetes and pharyngeal cancer, as opposed to previous literature [36], might be attributable to the difference in study design, population characteristics, or diagnostic criteria. Importantly, with ecological design, this study was subject to potential ecological fallacy, and thus further studies exploring the mechanism of DM in causing pharyngeal cancer at individual levels are needed.

### 3.4 Temporal Trends

The current study shows that oropharyngeal cancer was increasing, with the increase being more evident among males, which might seem to contradict the decreasing trend in the prevalence of smoking globally after the introduction of smoking reduction programs worldwide [22]. However, a study on the time‐dependent effect of tobacco smoking in relation to head and neck cancer (HNC) actually showed that the risk of HNC, including oropharyngeal cancer, increased by smoking experience far in time from HNC diagnosis, more than 40 years [38], which might explain the lag in trends, and it is likely that the incidence trend of oropharyngeal cancer might not have peaked.

Moreover, the rising incidence of oropharyngeal cancer might be associated with the increasing prevalence of HPV infection. Although the HPV vaccination program has been introduced for more than a decade, the introduction rate remained low in some regions, especially in Africa, where an introduction rate lower than 40% was reported [39]. A systematic review of population‐based studies assessed 13 studies concerning the trends of HPV‐related and ‐unrelated HNC subsites from 1965 to 2015 [40]. The potential HPV‐related HNC showed a significant increase among countries assessed except for Slovenia [40]. HPV drives infected cells toward malignant transformation by overexpressing the E6 and E7 early oncoproteins, which confer unlimited proliferative capacity through degradation or inactivation of tumor suppressor proteins [41]. The rising prevalence of HPV‐related HNC in Western countries may be driven by increasing oral HPV infection, likely influenced by changes in sexual behavior [42]. The UK, the USA, Italy, Norway, Canada, and some other developed regions have experienced a rising incidence of HPV‐related HNC in the past decades [42, 43]. The subsequent incidence trend should be monitored to capture the effect of the intervention program on the trend of cancer.

As for nasopharyngeal cancer, little change was recorded in trend in the current study. Due to its most prominent established risk factors, the consumption of salted or smoked fish in East Asia [20, 35], it is unlikely that the dietary habit would change suddenly. On the other hand, EBV is detected in 30%–90% of nasopharyngeal carcinomas [41], with antibody titers reaching 100% in Stage IV patients, and is classified as a Group I carcinogen by the International Agency for Research on Cancer (IARC) [44]. The virus drives carcinogenesis through latent proteins such as LMP1 and EBNA, which disrupt cellular function by mimicking CD40 and tumor necrosis factor receptors, promote uncontrolled proliferation, and inhibit apoptosis [45]. While the precise mechanisms in epithelial cells remain under investigation, EBV is thought to contribute to malignant transformation in nasopharyngeal carcinomas by altering host epigenetic programming and interfering with key tumor suppressors such as p53 [46]. Evidence from the UK and USA indicated rising EBV seroprevalence in the UK and increasing EBV‐related nasopharyngeal cancer in the USA [47, 48], yet comprehensive global analyses of EBV burden and EBV‐associated nasopharyngeal cancer remain lacking.

For hypopharyngeal cancer, an overall decreasing trend was observed in the current study. Due to the increasing number of asbestos ban countries to 55 countries in 2018 [49], countries with regulated use of asbestos, it is forecasted that the trend of hypopharyngeal cancer would continue to decrease. Further studies are needed for the latency effect of asbestos on hypopharyngeal cancer.

### 3.5 Limitations

Some potential limitations should be drawn attention to in this study. First and foremost, under‐reporting and misclassification of pharyngeal cancer were possible, particularly in developing countries or regions, because of the underdevelopment of infrastructure and mechanisms of cancer registries. Hence, bias could exist in the risk factor analysis. For hypertension and lipid disorders, there may be less bias, as developing countries also report a higher prevalence. Secondly, potential overestimation or underestimation might exist, since figures for some countries were derived from cancer registries located in major cities. Additionally, direct comparisons might be inappropriate for some countries since the cancer reporting infrastructure might have changed over time. Moreover, other registry‐related issues, including heterogeneity in completeness, possible underreporting in low‐resource settings, and shifts in diagnostic practices or classification systems, may also influence the reporting of cancer cases. However, because our analyses compared countries, regions, sexes, and age groups within the same time period, the potential biases arising from registry differences are unlikely to systematically affect the findings, as such effects would tend to balance out across groups. Lastly, the ecological design of this study would raise the possibility of ecological fallacy when interpreting associations between national‐level risk factors and pharynx cancer incidence.

### 3.6 Conclusions

As a remarkable regional difference was observed for the incidence of different subtypes of pharyngeal cancer, region‐based cancer preventive strategies should be developed. These included intensive modifications to the lifestyle and dietary habits, as they were closely linked to the increased risk of pharyngeal cancer. In addition, regarding the close association between HPV and oropharyngeal cancer, governments may consider promoting HPV vaccination to curb the rising incidence. Although there has been a decline in the prevalence of smoking after the implementation of smoking reduction programs, there was an overall increase in the incidence of oropharyngeal cancer, possibly due to the latency period between the smoking experience and diagnosis of the cancer. Therefore, initiating or strengthening smoking control programs is recommended to further reduce the global cancer burden attributable to tobacco use. High‐incidence regions of pharyngeal cancer, such as Polynesia, Northern America, and Eastern Asia, may benefit from implementing targeted early detection programs in the high‐risk populations. Further studies should continue to monitor the incidence and mortality trend and explore the impact of the COVID‐19 pandemic on the epidemiology of pharyngeal cancer.

## 4 Methods

### 4.1 Data Sources

The pharyngeal cancer incidence for 185 countries in 2020 were extracted from the Global Cancer Observatory (GLOBOCAN) database. GLOBOCAN is an online database maintained through a collaboration between the International Association of Cancer Registries, the World Health Organization, and population‐based cancer registries around the world. It contains statistics on the incidence and mortality rates of 26 cancer types globally [50]. According to the availability of data in each country, GLOBOCAN applies different methods for estimation. When high‐quality and representative local data are available, GLOBOCAN directly uses these data for its calculations. On the other hand, key cancer statistics, such as incidence‐to‐mortality ratios, trend predictions, and estimates would be computed for countries lacking direct data by referring to international and national cancer registry data [4]. The GLOBOCAN database was used to access the global incidence of pharyngeal cancer and for the risk factor analysis.

The Cancer Incidence in Five Continents Plus (CI5 Plus) database was utilized to obtain the proportions of pharyngeal cancer incidence by anatomical site, as well as the most recent 10‐year cancer incidence from 2003 to 2012 across 108 countries. As a comprehensive source of historical and current cancer incidence data, CI5 Plus enabled analyses of trends and changes over time by providing at least 15 consecutive years of incidence data for 122 selected populations from 106 cancer registries [51]. Registries were included in CI5 Plus only if they met strict quality criteria set by the IARC and the International Association of Cancer Registries, ensuring completeness and diagnostic accuracy [51]. The cancer site dictionary in CI5 Plus comprises 170 diagnostic units defined at the third‐digit level of International Classification of Diseases (ICD)‐10 categories, with additional histological subtypes for 13 cancer sites, thereby enabling consistent comparisons across populations. Since GLOBOCAN and CI5 Plus covered different time frames, the analysis on incidence and trend of pharyngeal cancer were separated to prevent potential bias.

The Global Burden of Disease (GBD) database was accessed for country‐specific risk factor data, including the prevalence of smoking, alcohol use, unhealthy diet, physical inactivity, obesity, hypertension, diabetes, and lipid disorders. The GBD 2019 database is a collaborative effort by over 7000 researchers from 156 countries to assess health loss caused by diseases, injuries, and risk factors [52]. GBD estimates risk factor exposure for 204 countries using a standardized modeling framework [53]. DisMod‐MR statistical model was used to impute the risk factor exposure in countries or regions without direct or high‐quality data, ensuring consistency and comparability across countries. Additionally, HDI and GDP per capita data for each country were extracted from the United Nations (UN) and World Bank, respectively [54, 55]. The GBD database was used in risk factor analysis along with the GLOBOCAN database. In addition, the GBD database was not used in the incidence analysis as the database only provided data for nasopharynx cancer and the other two subtypes of pharynx cancer were not as complete as estimated in this paper. Although data were obtained from three reliable official sources, the datasets were independent and non‐overlapping.

### 4.2 Statistical Analysis

The univariable linear regression analysis was performed to assess the relationship between pharyngeal cancer incidence and various risk factors, including HDI, GDP per capita, as well as lifestyle and metabolic risk factors. This analysis was conducted separately by sex and age group. The resulting *β* and 95% CIs were reported, where *β* represents the degree of change in the outcome variable (ASR) per unit increase in the predictor variable. Statistical significance was defined as a *p*‐value less than 0.05. Trend analysis was carried out using the Joinpoint regression software developed by the United States National Cancer Institute's Surveillance, Epidemiology, and End Results (SEER) Program. This allowed calculation of the AAPC to characterize temporal trends in pharyngeal cancer incidence by region and country. The standard epidemiological practice of using the most recent 10 years of data was followed. The incidence data underwent logarithmic transformation, and standard errors were generated accordingly. Uncorrelated error variance (heteroscedastic) was assumed in the Joinpoint regression model. Based on the automatic selection of Joinpoint, a maximum of one joinpoint was allowed for regression. The final model was chosen using the Weighted Bayesian Information Criterion (BIC) method.

The AAPC results were used to illustrate the direction and magnitude of incidence trends over time, with a positive AAPC indicating a rising trend and a negative AAPC indicating a declining trend. The 95% CIs for the AAPC were also reported, as an overlapping range of 0 would suggest a steady, unchanging trend without a statistically significant increase or decrease. Moreover, pharyngeal cancer incidence rates were examined across different demographic groups, including by age (all ages, young 15–49, old 50–74), sex (male, female), and geographic region (Asia, Oceania, Americas, Europe, Africa). This allowed for a comprehensive assessment of the epidemiological patterns and burden of pharyngeal cancer worldwide.

## Author Contributions

**Junjie Huang:** conceptualization, supervision, writing – original draft, data curation, formal analysis. **Sze Chai Chan:** writing – original draft, data curation, formal analysis. **Wing Sze Pang:** writing – original draft. **Yat Ching Fung:** writing – original draft. **Shui Hang Chow:** writing – original draft. **Veeleah Lok:** writing – review and editing. **Lin Zhang:** writing – review and editing. **Xu Lin:** writing – review and editing. **Don Eliseo Lucero‐Prisno III:** writing – review and editing. **Wanghong Xu:** writing – review and editing. **Zhi‐Jie Zheng:** writing – review and editing. **Edmar Elcarte:** writing – review and editing. **Mellissa Withers:** writing – review and editing. **Claire Chenwen Zhong:** writing – original draft, data curation, formal analysis. **Martin C. S. Wong:** writing – review and editing, conceptualization, supervision.

## Funding

The authors have nothing to report.

## Ethics Statement

This study was approved by the Survey and Behavioral Research Ethics Committee, The Chinese University of Hong Kong (No. SBRE‐20‐332).

## Consent

The authors have nothing to report.

## Conflicts of Interest

The authors declare no conflicts of interest.

## Supporting information


**Figure S1:** Pharyngeal cancer incidence trends by anatomical sites.
**Figure S2:** Plots of Joinpoint regression for trend analysis by anatomical sites.
**Table S1:** Risk factors associations for pharyngeal cancer incidence by anatomical sites.
**Table S2:** Joinpoint regression for pharyngeal cancer incidence by anatomical sites.

## Data Availability

The data that support the findings of this study are available from the corresponding author upon reasonable request.
