# Supplementary material for: Global Incidence, Risk Factors, and Trends of Pharyngeal Cancer by Anatomical Sites: A Systematic Analysis of Cancer Registries
Source: Cancer Rep (Hoboken). 2026 Jul 15;9(7):e70590. doi: 10.1002/cnr2.70590 (PMC13371618; doi:10.1002/cnr2.70590)
Supplement: Supplementary file 1 — Figure S1: Pharyngeal cancer incidence trends by anatomical sites. Figure S2: Plots of Joinpoint regression for trend analysis by anatomical sites. Table S1: Risk factors associations for pharyngeal cancer incidence by anatomical sites. Table S2: Joinpoint regression for pharyngeal cancer incidence by anatomical sites. [file CNR2-9-e70590-s001.docx]

# Supplementary Legends

**Figure S1.** Pharyngeal cancer incidence trends by anatomical sites

**Figure S2.** Plots of Joinpoint regression for trend analysis by anatomical sites

**Table S1.** Risk factors associations for pharyngeal cancer incidence by anatomical sites

**Table S2.** Joinpoint regression for pharyngeal cancer incidence by anatomical sites

**Figure S1. Pharyngeal cancer incidence trends by anatomical sites**

Bahrain: incidence

Age-standard (World) incidence per 100 000

**Pharynx**

# Asia

China: Incidence

Age-standard (World) incidence per 100 000

India: Incidence

Age-standard (World) incidence per 100 000

6

|  |
| --- |
|  |
|  |

4

2

0

2003 2006 2009 2012

Male Female

Both Young (15-49)

Old (50-74)

Age-standard (World) incidence per 100 000

1.5

1

0.5

0

|  |
| --- |
|  |
|  |

2003 2006 2009 2012

Male Female

Both Young (15-49)

Old (50-74)

10

8

6

4

2

0

2003 2006 2009 2012

Male Female

Both Young (15-49)

Old (50-74)

Age-standard (World) incidence per 100 000

Israel: Incidence

Age-standard (World) incidence per 100 000

Japan: Incidence

Korea: Incidence

2

1.5

1

0.5

0

2003 2006 2009 2012

Male Female

Both Young (15-49)

Old (50-74)

4

3

2

1

0

2003 2006 2009 2012

Male Female

Both Young (15-49)

Old (50-74)

4

3

2

1

0

2003 2006 2009 2012

Male Female

Both Young (15-49)

Old (50-74)

Kuwait: Incidence

Age-standard (World) incidence per 100 000

Philippines: Incidence

Thailand: Incidence

3

|  |
| --- |
|  |
|  |

2

1

0

2003 2006 2009 2012

Male Female

Both Young (15-49)

Old (50-74)

3

2

|  |
| --- |
|  |
|  |

1

0

2003 2006 2009 2012

Male Female

Both Young (15-49)

Old (50-74)

4

3

2

1

0

2003 2006 2009 2012

Male Female

Both Young (15-49）

Old (50-74)

Turkey: Incidence

Age-standard (World) incidence per 100 000

Age-standard (World) incidence per 100 000

Age-standard (World) incidence per 100 000

3

|  |
| --- |
|  |
|  |

2

1

0

2003 2006 2009 2012

Male Female

Both Young (15-49)

Old (50-74)

Australia: Incidence

Age-standard (World) incidence per 100 000

# Oceania

New Zealand: Incidence

Age-standard (World) incidence per 100

000

10

5

0

2003 2006 2009 2012

Male Female

Both Young (15-49)

Old (50-74)

8

6

4

2

0

2003 2006 2009 2012

Male Female

Both Young (15-49)

Old (50-74)

Canada: Incidence

Age-standard (World) incidence per 100 000

8

6

4

2

0

2003 2006 2009 2012

Male Female

Both Young (15-49)

Old (50-74)

# Northern America

USA: Incidence

Age-standard (World) incidence per 100 000

10

5

0

2003 2006 2009 2012

Male Female

Both Young (15-49)

Old (50-74)

Brazil: Incidence

Age-standard (World) incidence per 100 000

# Southern America

Chile: Incidence

Age-standard (World) incidence per 100 000

Colombia: Incidence

Age-standard (World) incidence per 100 000

10

8

6

4

2

0

2003 2006 2009 2012

Male Female

Both Young (15-49)

Old (50-74)

2

1.5

1

0.5

0

2003 2006 2009 2012

Male Female

Both Young (15-49)

Old (50-74)

5

4

3

2

1

0

2003 2006 2009 2012

Male Female

Both Young (15-49)

Old (50-74)

Ecuador: Incidence

Age-standard (World) incidence per 100 000

3

|  |
| --- |
|  |
|  |

2

1

0

2003 2006 2009 2012

Male Female

Both Young (15-49)

Old (50-74)

Martinique: Incidence

10

Age-standard (World) incidence per 100 000

5

0

2003 2006 2009 2012

Male Female

Both Young (15-49)

Old (50-74)

Denmark: Incidence

Age-standard (World) incidence per 100 000

15

|  |
| --- |
|  |
|  |

10

5

0

2003 2006 2009 2012

Male Female

Both Young (15-49)

Old (50-74)

# Northern Europe

Estonia: Incidence

Age-standard (World) incidence per 100 000

8

6

4

2

0

2003 2006 2009 2012

Male Female

Both Young (15-49)

Old (50-74)

Iceland: Incidence

10

Age-standard (World) incidence per 100 000

5

0

2003 2006 2009 2012

Male Female

Both Young (15-49)

Old (50-74)

Ireland: Incidence

Age-standard (World) incidence per 100 000

8

6

4

2

0

2003 2006 2009 2012

Male Female

Both Young (15-49)

Old (50-74)

Lithuania: Incidence

10

Age-standard (World) incidence per 100 000

8

6

4

2

0

2003 2006 2009 2012

Male Female

Both Young (15-49)

Old (50-74)

Norway: Incidence

10

Age-standard (World) incidence per 100 000

8

6

4

2

0

2003 2006 2009 2012

Male Female

Both Young (15-49)

Old (50-74)

United Kingdom: Incidence

Age-standard (World) incidence per 100 000

10

5

0

2003 2006 2009 2012

Male Female

Both Young (15-49)

Old (50-74)

Austria: Incidence

Age-standard (World) incidence per 100 000

# Western Europe

France: Incidence

Age-standard (World) incidence per 100 000

Germany: Incidence

Age-standard (World) incidence per 100 000

10

8

6

4

2

0

2003 2006 2009 2012

Male Female

Both Young (15-49)

Old (50-74)

20

15

10

5

0

2003 2006 2009 2012

Male Female

Both Young (15-49)

Old (50-74)

15

10

|  |
| --- |
|  |
|  |

5

0

2003 2006 2009 2012

Male Female

Both Young (15-49)

Old (50-74)

Netherlands: Incidence Switzerland: Incidence

Age-standard (World) incidence per 100 000

Age-standard (World) incidence per 100 000

8

6

4

2

0

2003 2006 2009 2012

Male Female

Both Young (15-49)

Old (50-74)

15

10

|  |
| --- |
|  |
|  |

5

0

2003 2006 2009 2012

Male Female

Both Young (15-49)

Old (50-74)

Croatia: Incidence

Age-standard (World) incidence per 100 000

# Southern Europe

Cyprus: Incidence

Age-standard (World) incidence per 100

000

Italy: Incidence

Age-standard (World) incidence per 100 000

8

6

4

2

0

2003 2006 2009 2012

Male Female

Both Young (15-49)

Old (50-74)

3

2

|  |
| --- |
|  |
|  |

1

0

2003 2006 2009 2012

Male Female

Both Young (15-49)

Old (50-74)

8

6

4

2

0

2003 2006 2009 2012

Male Female

Both Young (15-49)

Old (50-74）

Age-standard (World) incidence per 100 000

Age-standard (World) incidence per 100 000

Malta: Incidence

Age-standard (World) incidence per 100 000

Slovenia: Incidence

Spain: Incidence

10

8

6

4

2

0

2003 2006 2009 2012

Male Female

Both Young (15-49)

Old (50-74)

15

10

|  |
| --- |
|  |
|  |

5

0

2003 2006 2009 2012

Male Female

Both Young (15-49)

Old (50-74)

8

6

4

2

0

2003 2006 2009 2012

Male Female

Both Young (15-49)

Old (50-74)

Bulgaria: Incidence

Czech Republic: Incidence

Poland: Incidence

6

15

6

4

10

4

2

5

2

0

0

0

2003 2006

2009

Female

2012

2003

Male Both

2006

2003

2006

2009

2012

Male

Both

Old (50-74)

2009

Female

2012

Young (15-49)

Young (15-49)

Male

Both

Old (50-74)

Female

Young (15-49)

Old (50-74)

**Eastern Europe**

Uganda: Incidence

5

4

3

2

1

0

2003

2006

Male

Both

Old (50-74)

2009 2012

Female

Young (15-49)

**Africa**

Age-standard (World) incidence per 100 000

Age-standard (World) incidence per 100 000

Age-standard (World) incidence per 100 000

Age-standard (World) incidence per 100 000

|  |
| --- |
|  |
|  |

|  |
| --- |
|  |
|  |

|  |
| --- |
|  |
|  |

Bahrain: Incidence

Age-standard (World) incidence per 100 000

**Oropharynx**

# Asia

China: Incidence

Age-standard (World) incidence per 100 000

India: Incidence

Age-standard (World) incidence per 100 000

4

3

2

1

0

2003 2006 2009 2012

Male Female

Both Young (15-49)

Old (50-74)

1.5

1

0.5

0

|  |
| --- |
|  |
|  |

2003 2006 2009 2012

Male Female

Both Young (15-49)

Old (50-74)

Age-standard (World) incidence per 100 000

10

8

6

4

2

0

2003 2006 2009 2012

Male Female

Both Young (15-49)

Old (50-74)

Age-standard (World) incidence per 100 000

Israel: Incidence

Age-standard (World) incidence per 100 000

Japan: Incidence

Korea: Incidence

2

1.5

1

0.5

0

2003 2006 2009 2012

Male Female

Both Young (15-49)

Old (50-74)

5

4

3

2

1

0

2003 2006 2009 2012

Male Female

Both Young (15-49)

Old (50-74)

4

3

2

1

0

2003 2006 2009 2012

Male Female

Both Young (15-49)

Old (50-74)

Kuwait: Incidence

Age-standard (World) incidence per 100 000

Philippines: Incidence

Thailand: Incidence

2.5

2

1.5

1

0.5

0

2003 2006 2009 2012

Male Female

Both Young (15-49)

Old (50-74)

2.5

2

1.5

1

0.5

0

2003 2006 2009 2012

Male Female

Both Young (15-49)

Old (50-74)

2.5

2

1.5

1

0.5

0

2003 2006 2009 2012

Age-standard (World) incidence per 100 000

Male Female

Both Young (15-49)

Old (50-74)

Turkey: Incidence

Age-standard (World) incidence per 100 000

Age-standard (World) incidence per 100 000

2.5

2

1.5

1

0.5

0

2003 2006 2009 2012

Male Female

Both Young (15-49)

Old (50-74)

Australia: Incidence

Age-standard (World) incidence per 100 000

# Oceania

New Zealand: Incidence

Age-standard (World) incidence per 100 000

8

7

6

5

4

3

2

1

0

2003 2006 2009 2012

Male Female

Both Young (15-49)

Old (50-74)

6

4

|  |
| --- |
|  |
|  |

2

0

2003 2006 2009 2012

Male Female

Both Young (15-49)

Old (50-74)

Canada: Incidence

Age-standard (World) incidence per 100 000

10

# Northen America

USA: Incidence

Age-standard (World) incidence per 100 000

10

5 5

0

2003 2006 2009 2012

Male Female

Both Young (15-49)

Old (50-74)

0

2003 2006 2009 2012

Male Female

Both Young (15-49)

Old (50-74)

Brazil: Incidence

Age-standard (World) incidence per 100 000

15

|  |
| --- |
|  |
|  |

10

5

0

2003 2006 2009 2012

Male Female

Both Young (15-49)

Old (50-74)

2

1.5

Age-standard (World) incidence per 100 000

1

0.5

0

# Southern America

Chile: Incidence

2003 2006 2009 2012

Male Female

Both Young (15-49)

Colombia: Incidence

4

Age-standard (World) incidence per 100 000

3

2

1

0

2003 2006 2009 2012

Male Female

Both Young (15-49)

Old (50-74)

2

Age-standard (World) incidence per 100 000

1.5

1

0.5

0

Ecuador: Incidence

2003 2006 2009 2012

Male Female

Both Young (15-49)

Old (50-74)

Martinique: Incidence

15

Age-standard (World) incidence per 100 000

|  |
| --- |
|  |
|  |

10

5

0

2003 2006 2009 2012

Male Female

Both Young (15-49）

Old (50-74)

Denmark: Incidence

Age-standard (World) incidence per 100 000

20

15

10

5

0

2003 2006 2009 2012

Male Female

Both Young (15-49)

Old (50-74)

# Northern Europe

Estonia: Incidence

Age-standard (World) incidence per 100 000

8

6

4

2

0

2003 2006 2009 2012

Male Female

Both Young (15-49)

Old (50-74)

Iceland: Incidence

8

Age-standard (World) incidence per 100 000

6

4

2

0

2003 2006 2009 2012

Male Female

Both Young (15-49)

Old (50-74)

Ireland: Incidence

Age-standard (World) incidence per 100 000

6

|  |
| --- |
|  |
|  |

4

2

0

2003 2006 2009 2012

Male Female

Both Young (15-49)

Old (50-74)

Lithuania: Incidence

15

Age-standard (World) incidence per 100 000

|  |
| --- |
|  |
|  |

10

5

0

2003 2006 2009 2012

Male Female

Both Young (15-49)

Old (50-74)

Norway: Incidence

10

Age-standard (World) incidence per 100 000

5

0

2003 2006 2009 2012

Male Female

Both Young (15-49)

Old (50-74)

United Kingdom: Incidence

Age-standard (World) incidence per 100 000

10

8

6

4

2

0

2003 2006 2009 2012

Male Female

Both Young (15-49)

Old (50-74)

Austria: Incidence

Age-standard (World) incidence per 100 000

15

|  |
| --- |
|  |
|  |

10

5

0

2003 2006 2009 2012

Male Female

Both Young (15-49)

Old (50-74)

# Western Europe

France: Incidence

Age-standard (World) incidence per 100 000

20

15

10

5

0

2003 2006 2009 2012

Male Female

Both Young (15-49)

Old (50-74)

Germany: Incidence

20

Age-standard (World) incidence per 100 000

15

10

5

0

2003 2006 2009 2012

Male Female

Both Young (15-49)

Old (50-74)

Netherlands: Incidence

Age-standard (World) incidence per 100 000

8

6

4

2

0

2003 2006 2009 2012

Male Female

Both Young (15-49)

Old (50-74)

Switzerland: Incidence

15

Age-standard (World) incidence per 100 000

|  |
| --- |
|  |
|  |

10

5

0

2003 2006 2009 2012

Male Female

Both Young (15-49）

Old (50-74)

Croatia: Incidence

Age-standard (World) incidence per 100 000

# Southern Europe

Cyprus: Incidence

Age-standard (World) incidence per 100 000

Italy: Incidence

Age-standard (World) incidence per 100 000

10

8

6

4

2

0

2003 2006 2009 2012

Male Female

Both Young (15-49)

Old (50-74)

2.5

2

1.5

1

0.5

0

2003 2006 2009 2012

Male Female

Both Young (15-49)

Old (50-74)

8

6

4

2

0

2003 2006 2009 2012

Male Female

Both Young (15-49)

Old (50-74）

Malta: Incidence

Age-standard (World) incidence per 100 000

Slovenia: Incidence

Spain: Incidence

10

8

6

4

2

0

2003 2006 2009 2012

Male Female

Both Young (15-49）

Old (50-74)

20

15

10

5

0

2003 2006 2009 2012

Male Female

Both Young (15-49)

Old (50-74)

8

6

4

2

0

2003 2006 2009 2012

Male Female

Both Young (15-49)

Old (50-74)

Bulgaria: Incidence

# Eastern Europe

Czech Republic: Incidence

Age-standard (World) incidence per 100 000

Age-standard (World) incidence per 100 000

Age-standard (World) incidence per 100 000

Poland: Incidence

Age-standard (World) incidence per 100 000

6

Age-standard (World) incidence per 100 000

|  |
| --- |
|  |
|  |

4

2

0

2003 2006 2009 2012

Male Female

Both Young (15-49)

Old (50-74)

15

10

|  |
| --- |
|  |
|  |

5

0

2003 2006 2009 2012

Male Female

Both Young (15-49)

Old (50-74)

8

6

4

2

0

2003 2006 2009 2012

Male Female

Both Young (15-49)

Old (50-74)

**Africa**

Uganda: Incidence

6

5

4

3

2

1

0

2003 2006

Male

Both

2009 2012

Female

Young (15-49)

Old (50-74)

Age-standard (World) incidence per 100 000

Bahrain: incidence

Age-standard (World) incidence per 100 000

**Nasopharynx**

# Asia

China: Incidence

Age-standard (World) incidence per 100 000

India: Incidence

Age-standard (World) incidence per 100 000

15

|  |
| --- |
|  |
|  |

10

5

0

2003 2006 2009 2012

Male Female

Both Young (15-49)

Old (50-74)

Age-standard (World) incidence per 100 000

15

10

|  |
| --- |
|  |
|  |

5

0

2003 2006 2009 2012

Male Female

Both Young (15-49)

Old (50-74)

2.5

2

1.5

1

0.5

0

2003 2006 2009 2012

Male Female

Both Young (15-49)

Old (50-74)

Israel: Incidence

Age-standard (World) incidence per 100 000

Japan: Incidence

Korea: Incidence

2.5

2

1.5

1

0.5

0

2003 2006 2009 2012

Male Female

Both Young (15-49)

Old (50-74)

1.5

1

0.5

0

|  |
| --- |
|  |
|  |

2003 2006 2009 2012

Male Female

Both Young (15-49)

Old (50-74)

2.5

2

1.5

1

0.5

0

2003 2006 2009 2012

Age-standard (World) incidence per 100 000

Male Female

Both Young (15-49)

Old (50-74)

Kuwait: Incidence

Age-standard (World) incidence per 100 000

Philippines: Incidence

Thailand: Incidence

8

6

4

2

0

2003 2006 2009 2012

Male Female

Both Young (15-49)

Old (50-74)

15

10

|  |
| --- |
|  |
|  |

5

0

2003 2006 2009 2012

Male Female

Both Young (15-49)

Old (50-74)

10

8

6

4

2

0

2003 2006 2009 2012

Male Female

Both Young (15-49）

Old (50-74)

Turkey: Incidence

Age-standard (World) incidence per 100 000

Age-standard (World) incidence per 100 000

Age-standard (World) incidence per 100 000

5

4

3

2

1

0

2003 2006 2009 2012

Male Female

Both Young (15-49)

Old (50-74)

1.5

Age-standard (World) incidence per 100 000

1

0.5

0

Australia: Incidence

|  |
| --- |
|  |
|  |

2003 2006 2009 2012

Male Female

Both Young (15-49)

Old (50-74)

2.5

2

Age-standard (World) incidence per 100 000

1.5

1

0.5

0

# Oceania

New Zealand: Incidence

2003 2006 2009 2012

Male Female

Both Young (15-49)

Old (50-74)

2

Age-standard (World) incidence per 100 000

1.5

1

0.5

0

Canada: Incidence

2003 2006 2009 2012

Male Female

Both Young (15-49)

Old (50-74)

# Northern America

USA: Incidence

Age-standard (World) incidence per 100 000

3

|  |
| --- |
|  |
|  |

2

1

0

2003 2006 2009 2012

Male Female

Both Young (15-49)

Old (50-74)

Brazil: Incidence

Age-standard (World) incidence per 100 000

# Southern America

Colombia: Incidence

Age-standard (World) incidence per 100 000

Ecuador: Incidence

Age-standard (World) incidence per 100 000

8

6

4

2

0

2003 2006 2009 2012

Male Female

Both Young (15-49)

Old (50-74)

2.5

2

1.5

1

0.5

0

2003 2006 2009 2012

Male Female

Both Young (15-49)

Old (50-74)

1

0.8

0.6

0.4

0.2

0

2003 2006 2009 2012

Male Female

Both Young (15-49)

Old (50-74)

Martinique: Incidence

Age-standard (World) incidence per 100 000

4

3

2

1

0

2003 2006 2009 2012

Male Female

Both Young (15-49)

Old (50-74)

1.5

Age-standard (World) incidence per 100 000

1

0.5

0

Denmark: Incidence

|  |
| --- |
|  |
|  |

2003 2006 2009 2012

Male Female

Both Young (15-49)

Old (50-74)

2

1.5

Age-standard (World) incidence per 100 000

1

0.5

0

# Northern Europe

Estonia: Incidence

2003 2006 2009 2012

Male Female

Both Young (15-49)

Old (50-74)

Iceland: Incidence

6

Age-standard (World) incidence per 100 000

|  |
| --- |
|  |
|  |

4

2

0

2003 2006 2009 2012

Male Female

Both Young (15-49)

Old (50-74)

Ireland: Incidence

Age-standard (World) incidence per 100 000

Lithuania: Incidence

Norway: Incidence

2

1.5

1

0.5

0

2003 2006 2009 2012

Male Female

Both Young (15-49)

Old (50-74)

2

1.5

1

0.5

0

2003 2006 2009 2012

Male Female

Both Young (15-49)

Old (50-74)

1.5

1

0.5

0

|  |
| --- |
|  |
|  |

2003 2006 2009 2012

Age-standard (World) incidence per 100 000

Male Female

Both Young (15-49)

Old (50-74)

United Kingdom: Incidence

Age-standard (World) incidence per 100 000

Age-standard (World) incidence per 100 000

1

0.8

0.6

0.4

0.2

0

2003 2006 2009 2012

Male Female

Both Young (15-49)

Old (50-74)

Austria: Incidence

Age-standard (World) incidence per 100 000

# Western Europe

France: Incidence

Age-standard (World) incidence per 100 000

Germany: Incidence

Age-standard (World) incidence per 100 000

1.5

1

0.5

0

|  |
| --- |
|  |
|  |

2003 2006 2009 2012

Male Female

Both Young (15-49)

Old (50-74)

2

1.5

1

0.5

0

2003 2006 2009 2012

Male Female

Both Young (15-49)

Old (50-74)

3

2

|  |
| --- |
|  |
|  |

1

0

2003 2006 2009 2012

Male Female

Both Young (15-49)

Old (50-74)

Netherlands: Incidence Switzerland: Incidence

Age-standard (World) incidence per 100 000

Age-standard (World) incidence per 100 000

1.5

1

0.5

0

|  |
| --- |
|  |
|  |

2003 2006 2009 2012

Male Female

Both Young (15-49)

Old (50-74)

2

1.5

1

0.5

0

2003 2006 2009 2012

Male Female

Both Young (15-49)

Old (50-74)

Croatia: Incidence

Age-standard (World) incidence per 100 000

# Southern Europe

Cyprus: Incidence

Age-standard (World) incidence per 100 000

Italy: Incidence

Age-standard (World) incidence per 100 000

2.5

2

1.5

1

0.5

0

2003 2006 2009 2012

Male Female

Both Young (15-49)

Old (50-74)

3

2

|  |
| --- |
|  |
|  |

1

0

2003 2006 2009 2012

Male Female

Both Young (15-49)

Old (50-74)

3

2

|  |
| --- |
|  |
|  |

1

0

2003 2006 2009 2012

Male Female

Both Young (15-49)

Old (50-74）

Age-standard (World) incidence per 100 000

Age-standard (World) incidence per 100 000

Malta: Incidence

Age-standard (World) incidence per 100 000

Slovenia: Incidence

Spain: Incidence

8

6

4

2

0

2003 2006 2009 2012

Male Female

Both Young (15-49)

Old (50-74)

2

1.5

1

0.5

0

2003 2006 2009 2012

Male Female

Both Young (15-49)

Old (50-74)

4

3

2

1

0

2003 2006 2009 2012

Male Female

Both Young (15-49)

Old (50-74)

Bulgaria: Incidence

Czech Republic: Incidence

Poland: Incidence

3

2

1

0

2

1.5

1

0.5

0

2.5

2

1.5

1

0.5

0

2003 2006

2009

Female

2012

2003

Male Both

2006

2009 2012

Female

2003

Male Both

2006

2009

2012

Male

Both

Old (50-74)

Young (15-49)

Young (15-49)

Female

Young (15-49)

Old (50-74)

Old (50-74)

**Eastern Europe**

Uganda: Incidence

20

15

10

5

0

2003 2006

Male

Both

2009 2012

Female

Young (15-49)

Old (50-74)

**Africa**

Age-standard (World) incidence per 100 000

Age-standard (World) incidence per 100 000

Age-standard (World) incidence per 100 000

Age-standard (World) incidence per 100 000

|  |
| --- |
|  |
|  |

|  |
| --- |
|  |
|  |
|  |

|  |
| --- |
|  |
|  |
|  |

Bahrain: Incidence

Age-standard (World) incidence per 100 000

**Hypopharynx**

# Asia

China: Incidence

Age-standard (World) incidence per 100 000

India: Incidence

Age-standard (World) incidence per 100 000

3

|  |
| --- |
|  |
|  |

2

1

0

2003 2006 2009 2012

Male Female

Both Young (15-49)

Old (50-74)

2

1.5

1

0.5

0

2003 2006 2009 2012

Male Female

Both Young (15-49)

Old (50-74)

Age-standard (World) incidence per 100 000

20

15

10

5

0

2003 2006 2009 2012

Male Female

Both Young (15-49)

Old (50-74)

Age-standard (World) incidence per 100 000

Israel: Incidence

Age-standard (World) incidence per 100 000

Japan: Incidence

Korea: Incidence

0.8

0.6

0.4

0.2

0

2003 2006 2009 2012

Male Female

Both Young (15-49)

Old (50-74)

8

6

4

2

0

2003 2006 2009 2012

Male Female

Both Young (15-49)

Old (50-74)

3

2

|  |
| --- |
|  |
|  |

1

0

2003 2006 2009 2012

Male Female

Both Young (15-49)

Old (50-74)

Kuwait: Incidence

Age-standard (World) incidence per 100 000

Philippines: Incidence

Thailand: Incidence

2.5

2

1.5

1

0.5

0

2003 2006 2009 2012

Male Female

Both Young (15-49)

Old (50-74)

1.5

1

0.5

0

|  |
| --- |
|  |
|  |

2003 2006 2009 2012

Male Female

Both Young (15-49)

Old (50-74)

4

3

2

1

0

2003 2006 2009 2012

Male Female

Both Young (15-49)

Old (50-74)

Turkey: Incidence

Age-standard (World) incidence per 100 000

Age-standard (World) incidence per 100 000

Age-standard (World) incidence per 100 000

2.5

2

1.5

1

0.5

0

2003 2006 2009 2012

Male Female

Both Young (15-49)

Old (50-74)

Australia: Incidence

Age-standard (World) incidence per 100 000

# Oceania

New Zealand: Incidence

Age-standard (World) incidence per 100 000

5

4

3

2

1

0

2003 2006 2009 2012

Male Female

Both Young (15-49)

Old (50-74)

2

1.5

1

0.5

0

2003 2006 2009 2012

Male Female

Both Young (15-49)

Old (50-74)

2

Age-standard (World) incidence per 100 000

1.5

1

0.5

0

Canada: Incidence

2003 2006 2009 2012

Male Female

Both Young (15-49)

Old (50-74)

# Northern America

USA: Incidence

Age-standard (World) incidence per 100 000

3

|  |
| --- |
|  |
|  |

2

1

0

2003 2006 2009 2012

Male Female

Both Young (15-49)

Old (50-74)

Brazil: Incidence

Age-standard (World) incidence per 100 000

# Southern America

Chile: Incidence

Age-standard (World) incidence per 100 000

Colombia: Incidence

Age-standard (World) incidence per 100 000

10

8

6

4

2

0

2003 2006 2009 2012

Male Female

Both Young (15-49)

Old (50-74)

3

2

|  |
| --- |
|  |
|  |

1

0

2003 2006 2009 2012

Male Female

Both Young (15-49)

Old (50-74)

1

0.8

0.6

0.4

0.2

0

2003 2006 2009 2012

Male Female

Both Young (15-49)

Old (50-74)

0.1

Age-standard (World) incidence per 100 000

0.05

0

Ecuador: Incidence

2003 2006 2009 2012

Male Female

Both Young (15-49)

Old (50-74)

Martinique: Incidence

8

Age-standard (World) incidence per 100 000

6

4

2

0

2003 2006 2009 2012

Male Female

Both Young (15-49）

Old (50-74)

Denmark: Incidence

Age-standard (World) incidence per 100 000

8

6

4

2

0

2003 2006 2009 2012

Male Female

Both Young (15-49)

Old (50-74)

# Northern Europe

Estonia: Incidence

Age-standard (World) incidence per 100 000

6

|  |
| --- |
|  |
|  |

4

2

0

2003 2006 2009 2012

Male Female

Both Young (15-49)

Old (50-74)

2.5

2

Age-standard (World) incidence per 100 000

1.5

1

0.5

0

Iceland: Incidence

2003 2006 2009 2012

Male Female

Both Young (15-49)

Old (50-74)

Ireland: Incidence

Age-standard (World) incidence per 100 000

5

4

3

2

1

0

2003 2006 2009 2012

Male Female

Both Young (15-49)

Old (50-74)

Lithuania: Incidence

10

Age-standard (World) incidence per 100 000

8

6

4

2

0

2003 2006 2009 2012

Male Female

Both Young (15-49)

Old (50-74)

2.5

2

Age-standard (World) incidence per 100 000

1.5

1

0.5

0

Norway: Incidence

2003 2006 2009 2012

Male Female

Both Young (15-49)

Old (50-74)

United Kingdom: Incidence

Age-standard (World) incidence per 100 000

3

|  |
| --- |
|  |
|  |

2

1

0

2003 2006 2009 2012

Male Female

Both Young (15-49)

Old (50-74)

Austria: Incidence

Age-standard (World) incidence per 100 000

# Western Europe

France: Incidence

Age-standard (World) incidence per 100 000

Germany: Incidence

Age-standard (World) incidence per 100 000

6

|  |
| --- |
|  |
|  |

4

2

0

2003 2006 2009 2012

Male Female

Both Young (15-49)

Old (50-74)

20

15

10

5

0

2003 2006 2009 2012

Male Female

Both Young (15-49)

Old (50-74)

10

8

6

4

2

0

2003 2006 2009 2012

Male Female

Both Young (15-49)

Old (50-74)

Netherlands: Incidence Switzerland: Incidence

Age-standard (World) incidence per 100 000

Age-standard (World) incidence per 100 000

5

4

3

2

1

0

2003 2006 2009 2012

Male Female

Both Young (15-49)

Old (50-74)

15

10

|  |
| --- |
|  |
|  |

5

0

2003 2006 2009 2012

Male Female

Both Young (15-49）

Old (50-74)

Croatia: Incidence

Age-standard (World) incidence per 100 000

# Southern Europe

Cyprus: Incidence

Age-standard (World) incidence per 100 000

Italy: Incidence

Age-standard (World) incidence per 100 000

10

8

6

4

2

0

2003 2006 2009 2012

Male Female

Both Young (15-49)

Old (50-74)

1.5

1

0.5

0

|  |
| --- |
|  |
|  |

2003 2006 2009 2012

Male Female

Both Young (15-49)

Old (50-74)

Age-standard (World) incidence per 100 000

4

3

2

1

0

2003 2006 2009 2012

Male Female

Both Young (15-49)

Old (50-74）

Age-standard (World) incidence per 100 000

Malta: Incidence

Age-standard (World) incidence per 100 000

Slovenia: Incidence

Spain: Incidence

6

|  |
| --- |
|  |
|  |

4

2

0

2003 2006 2009 2012

Male Female

Both Young (15-49）

Old (50-74)

15

10

|  |
| --- |
|  |
|  |

5

0

2003 2006 2009 2012

Male Female

Both Young (15-49)

Old (50-74)

10

8

6

4

2

0

2003 2006 2009 2012

Male Female

Both Young (15-49)

Old (50-74)

Bulgaria: Incidence

# Eastern Europe

Czech Republic: Incidence

Age-standard (World) incidence per 100 000

Poland: Incidence

Age-standard (World) incidence per 100 000

4

Age-standard (World) incidence per 100 000

3

2

1

0

2003 2006 2009 2012

Male Female

Both Young (15-49)

Old (50-74)

5

4

3

2

1

0

2003 2006 2009 2012

Male Female

Both Young (15-49)

Old (50-74)

4

3

2

1

0

2003 2006 2009 2012

Male Female

Both Young (15-49)

Old (50-74)

# Africa

Uganda: Incidence

Age-standard (World) incidence per 100 000

12

10

8

6

4

2

0

2003 2006 2009 2012

Male Female

Both Young (15-49)

Old (50-74)

**Figure S2.** Plots of Joinpoint regression for trend analysis by anatomical sites

1. Pharynx - Male

| **Asia** | |
| --- | --- |
| 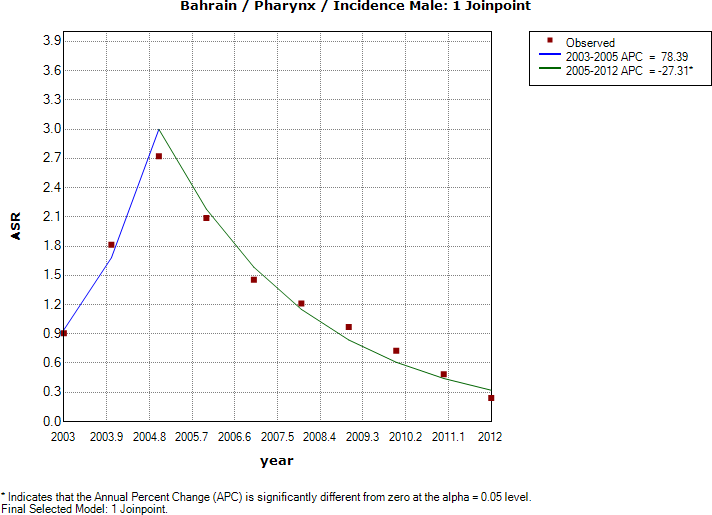 | 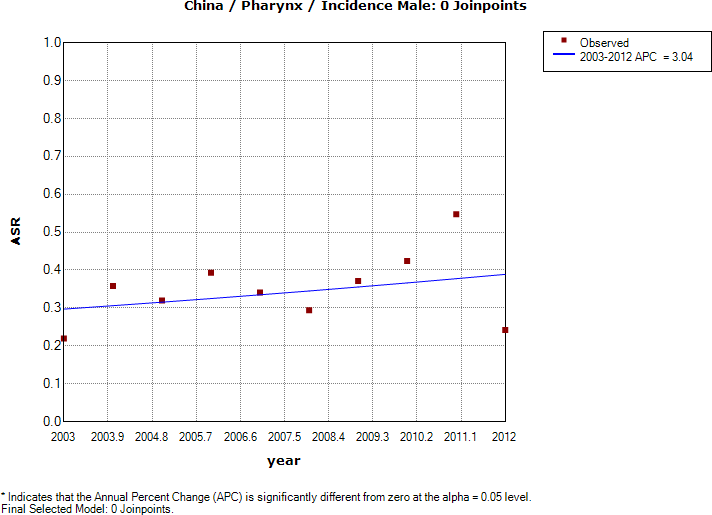 |
| 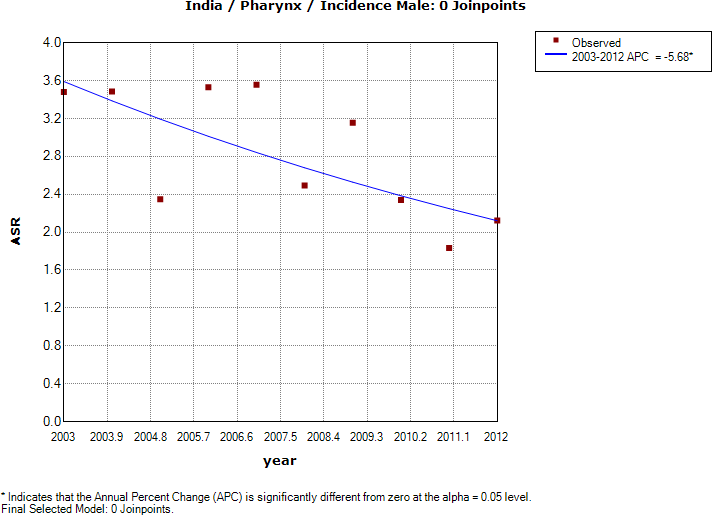 | 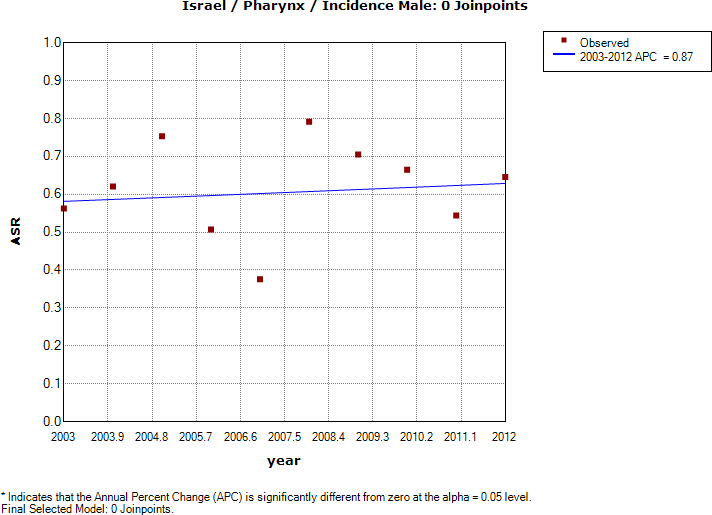 |
| 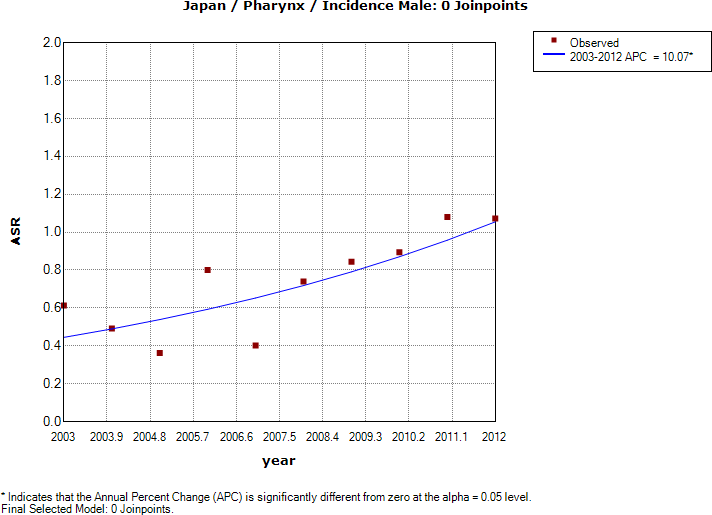 | 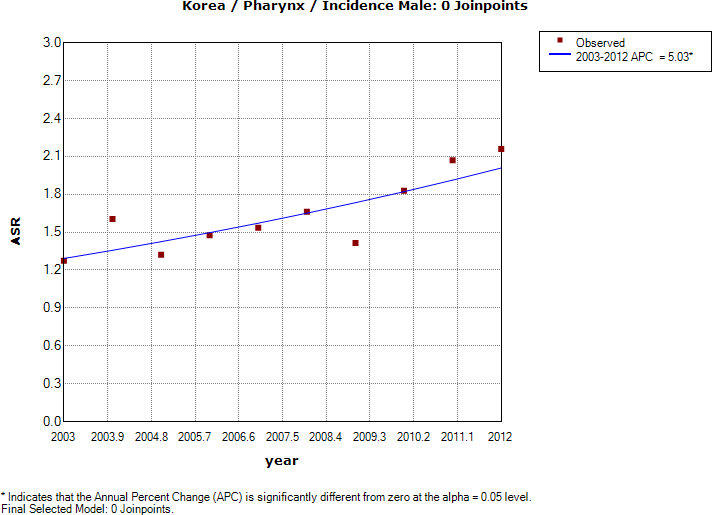 |

| 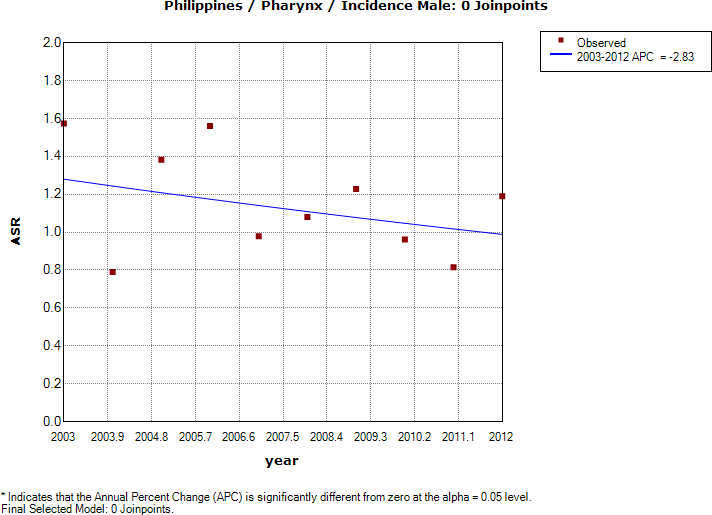 | 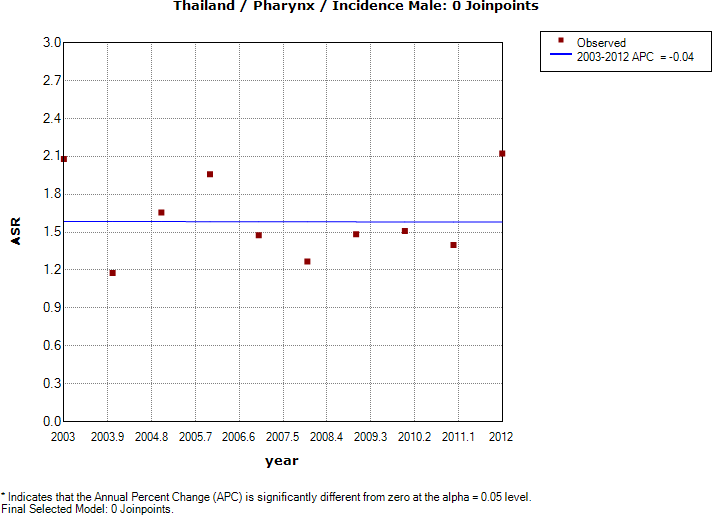 |
| --- | --- |
| 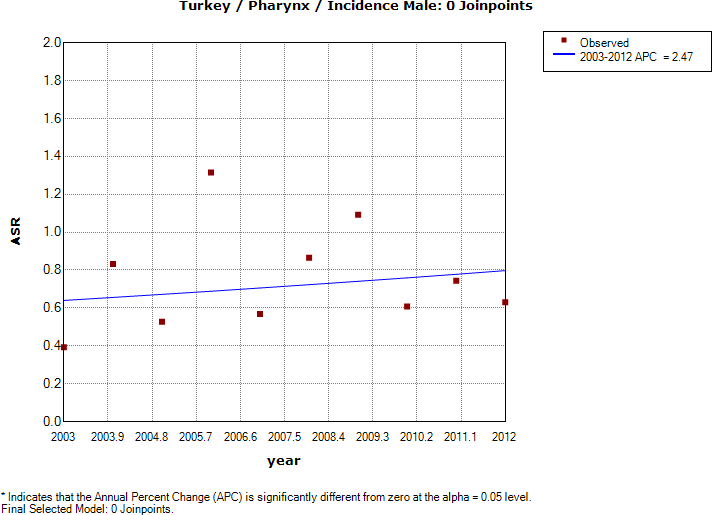 |  |
| **Oceania** | |
| 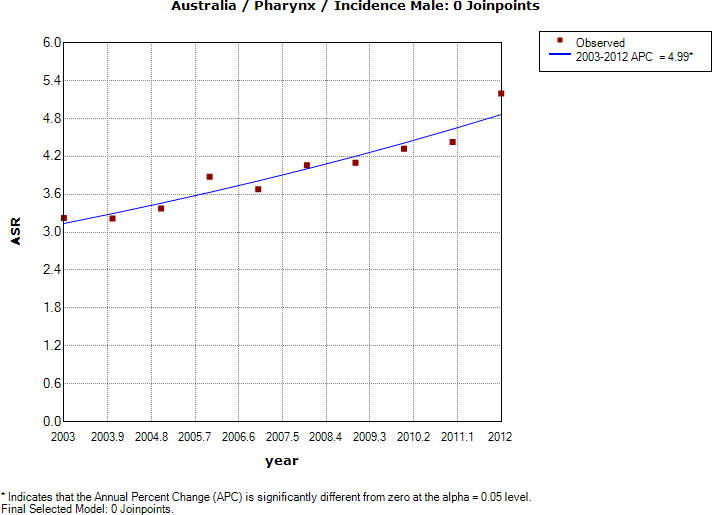 | 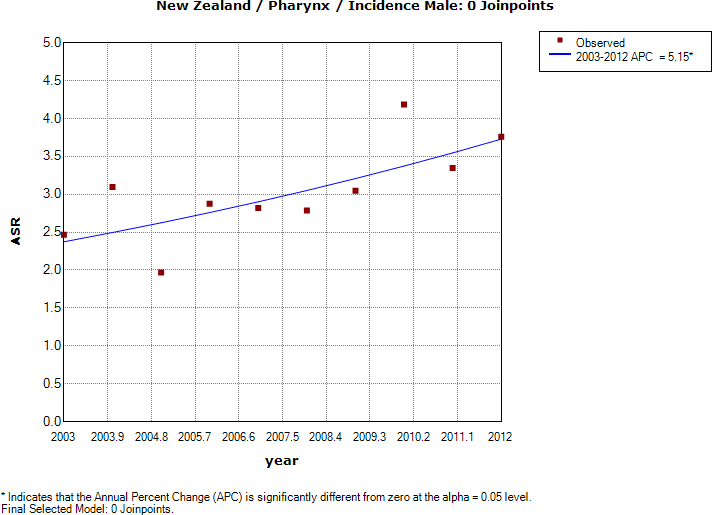 |

| **Northern America** | |
| --- | --- |
| 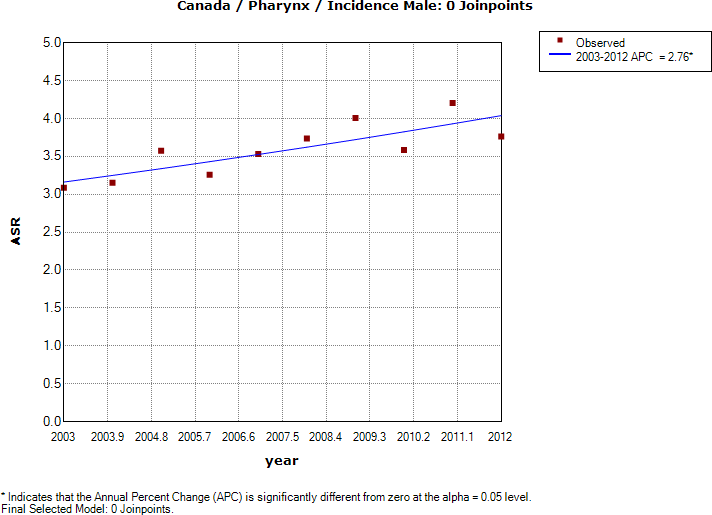 | 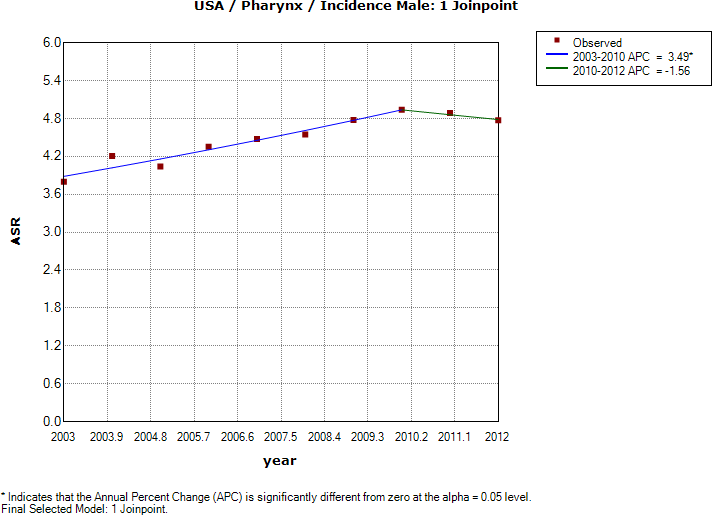 |
| **Southern America** | |
| 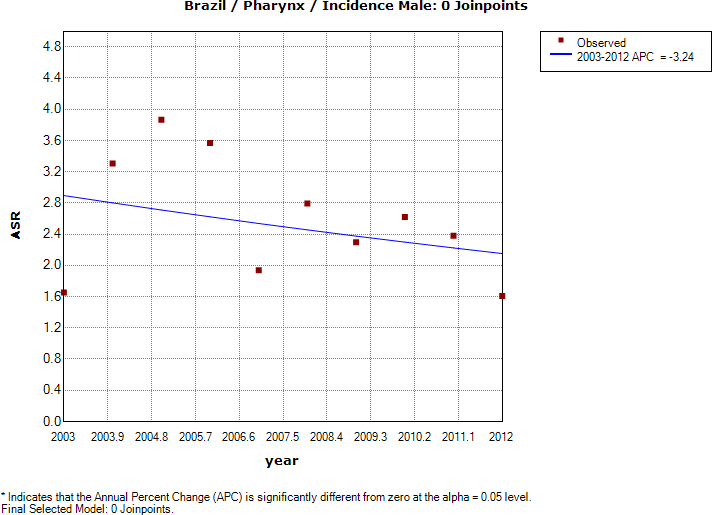 | 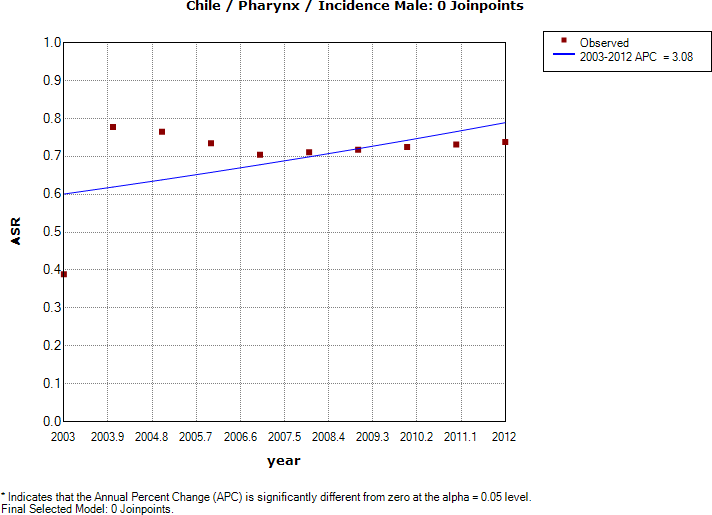 |
| 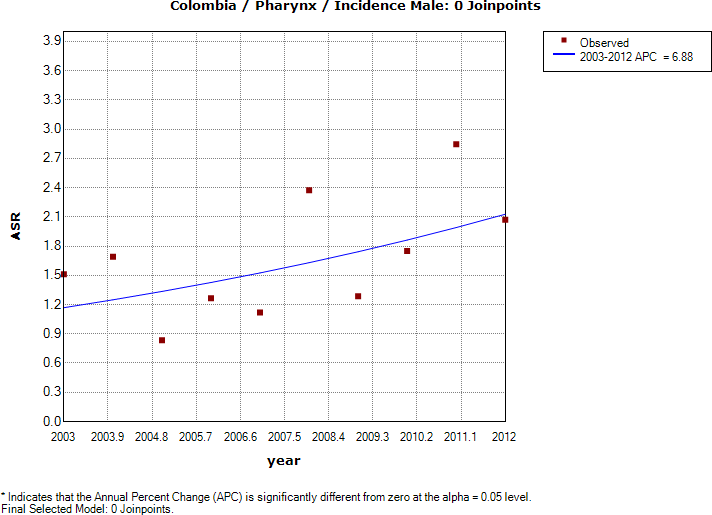 | 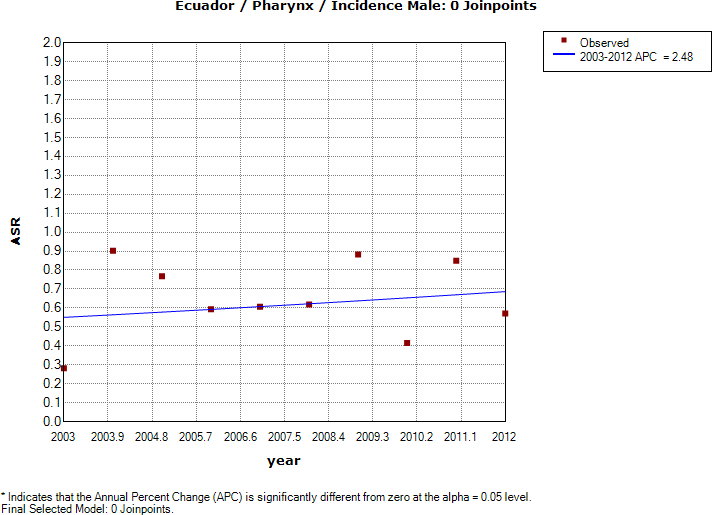 |

| 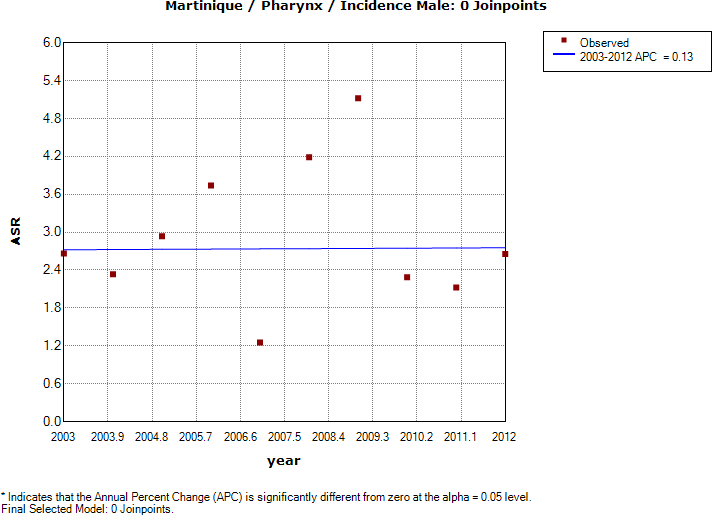 |  |
| --- | --- |
| **Northern Europe** | |
| 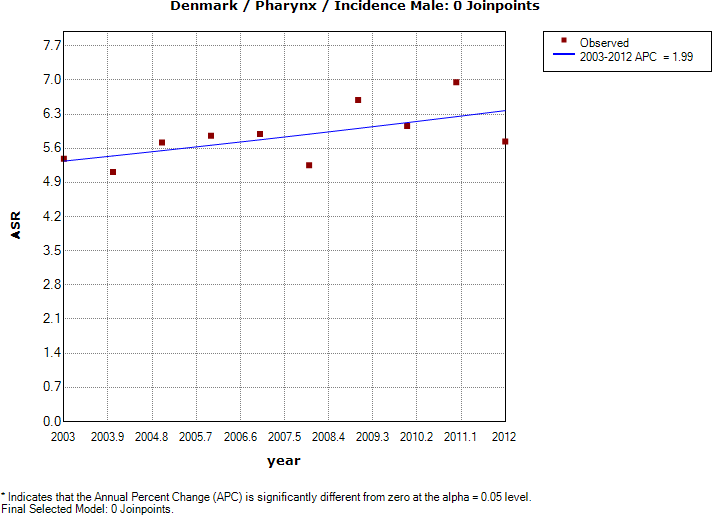 | 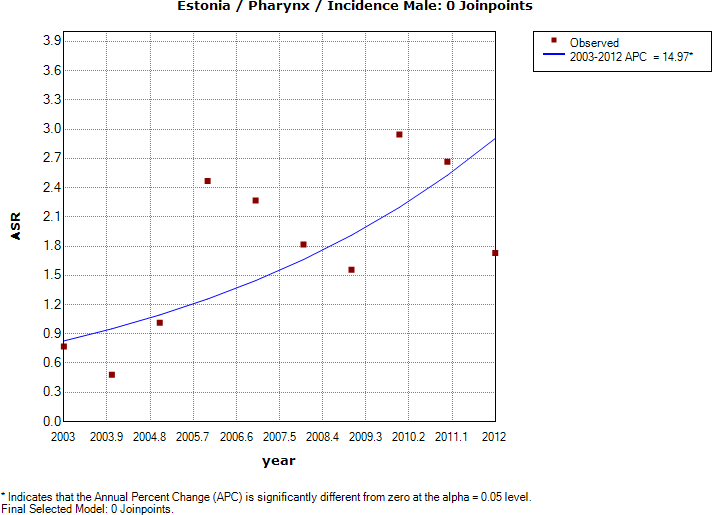 |
| 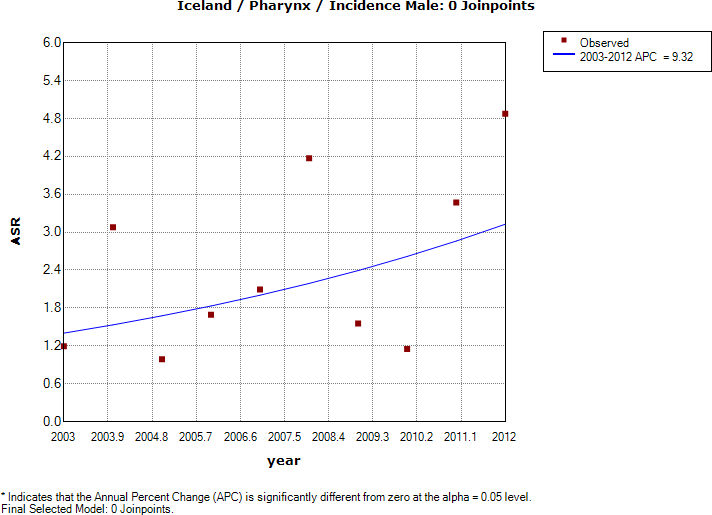 | 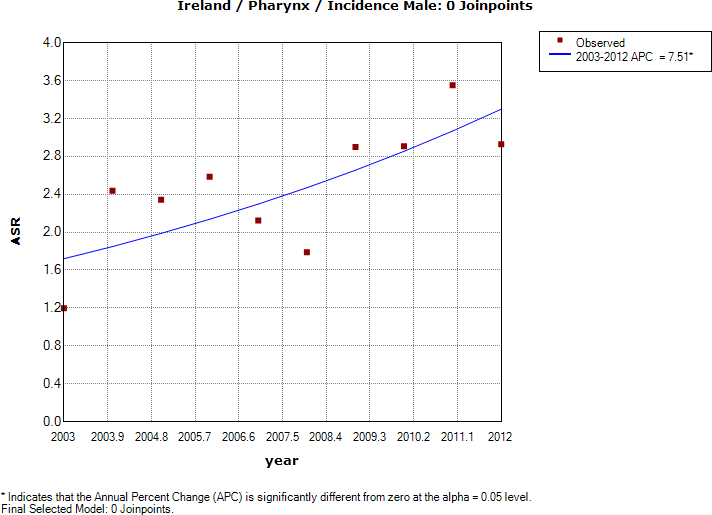 |
| 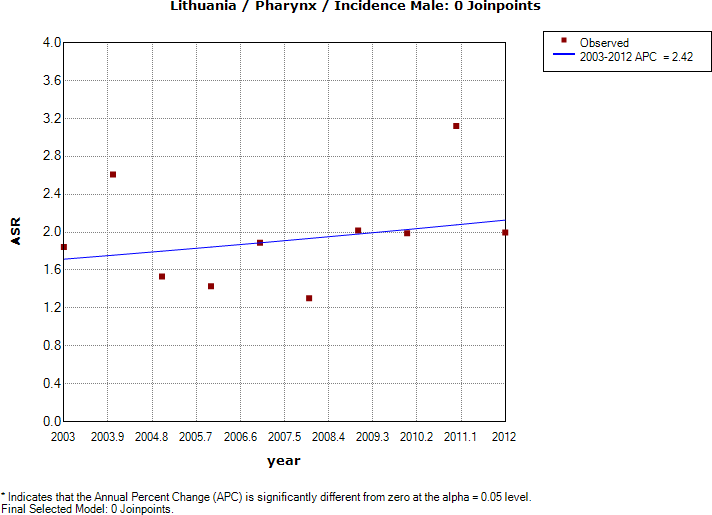 | 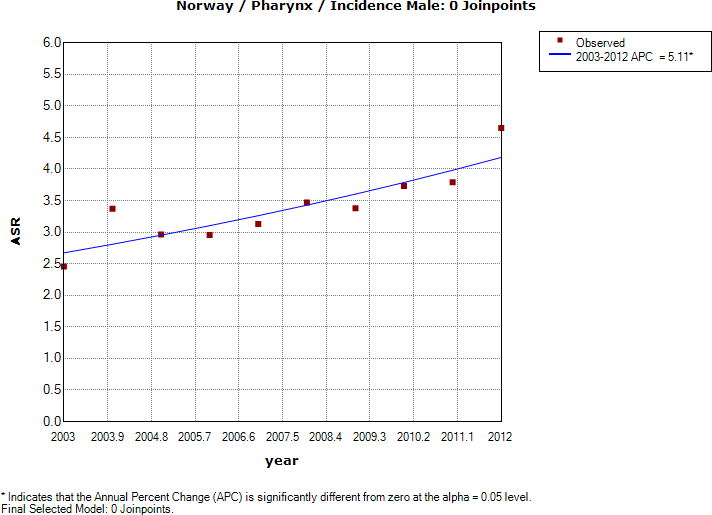 |

| 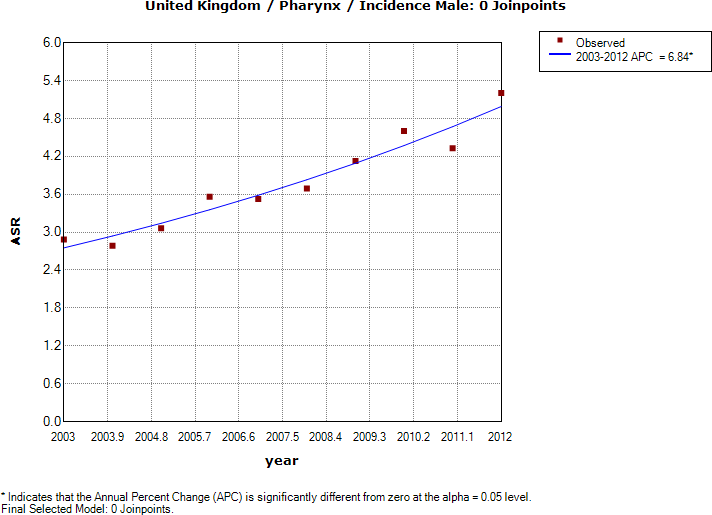 |  |
| --- | --- |
| **Western Europe** | |
| 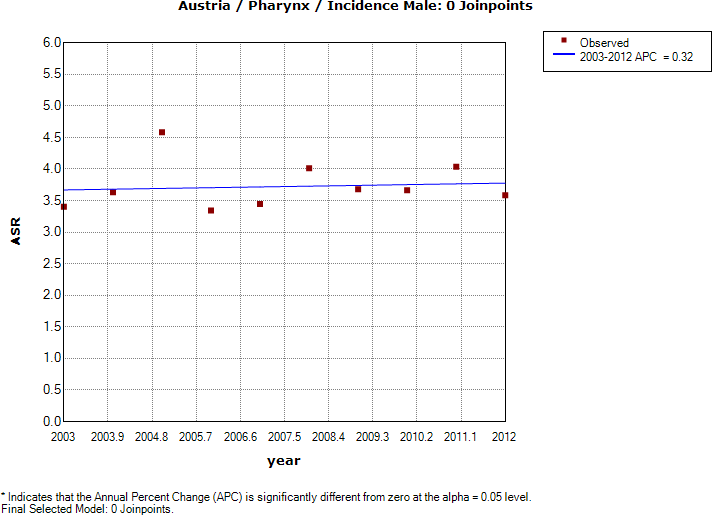 | 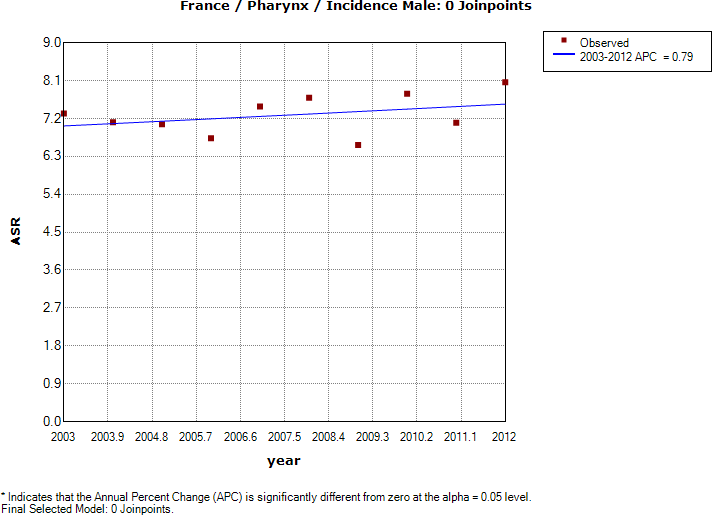 |
| 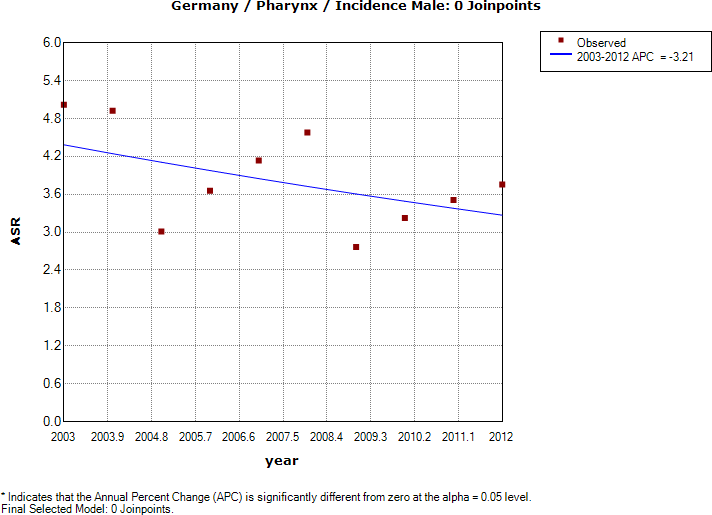 | 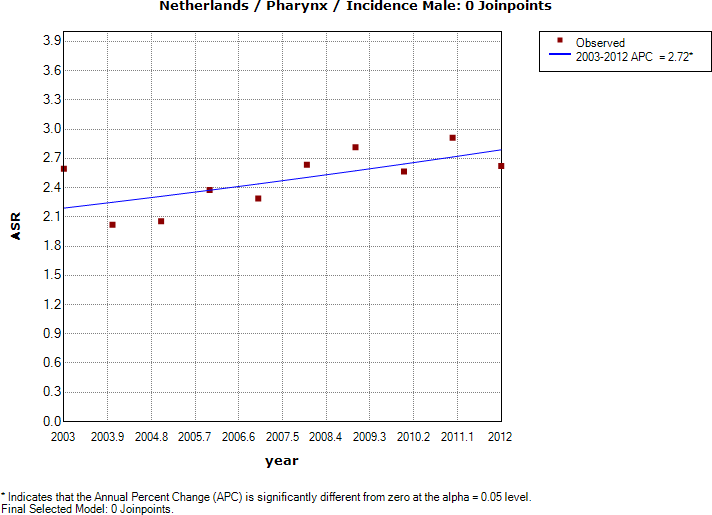 |
| 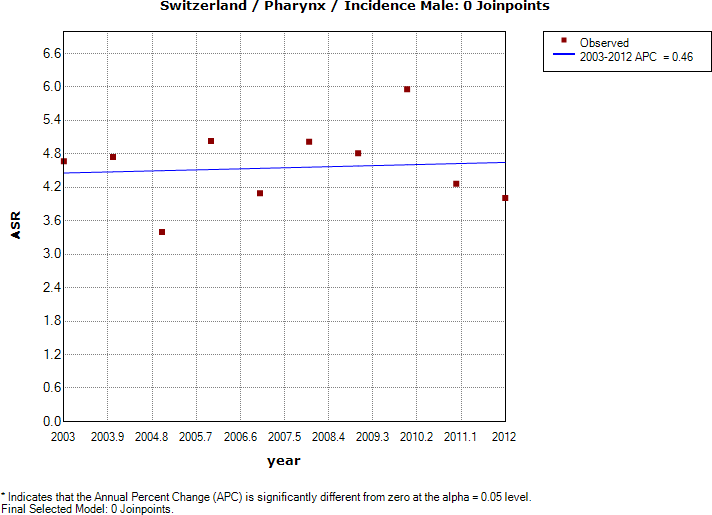 |  |

| **Southern Europe** | |
| --- | --- |
| 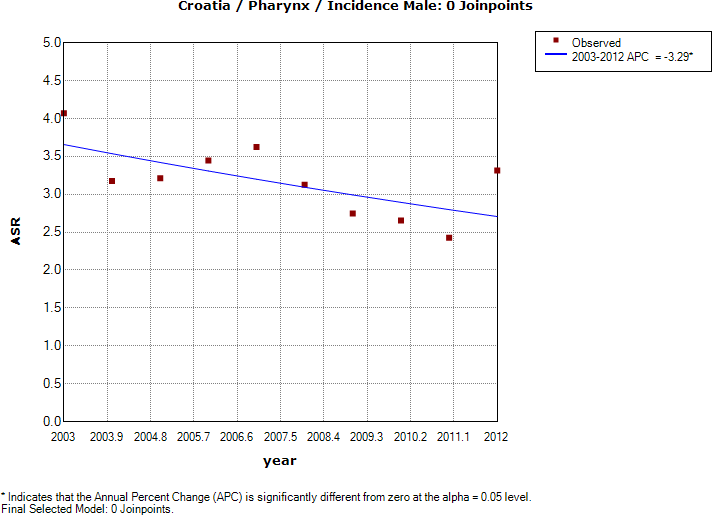 | 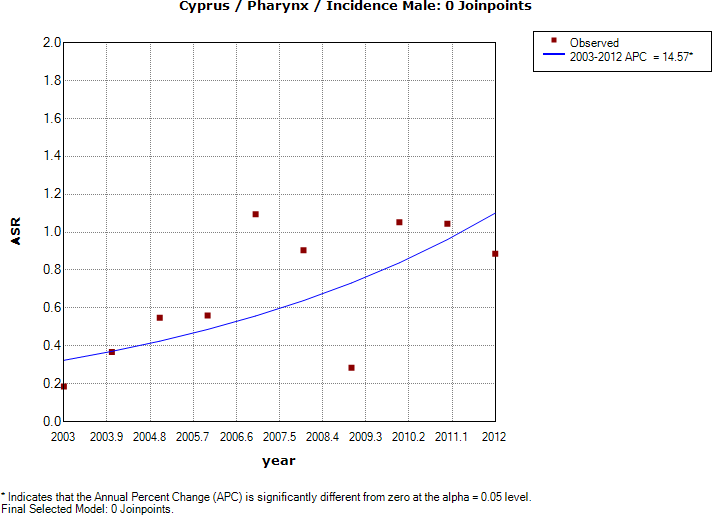 |
| 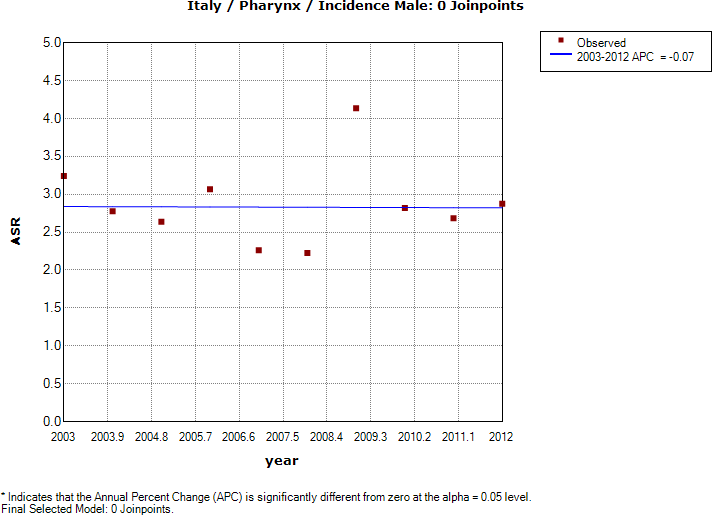 | 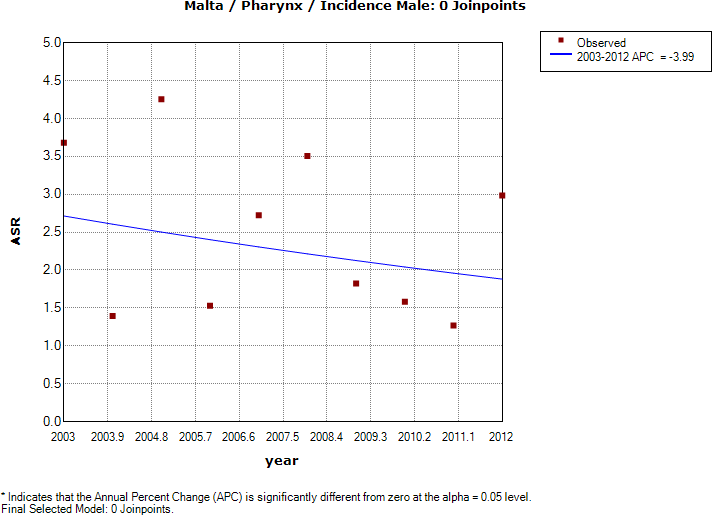 |
| 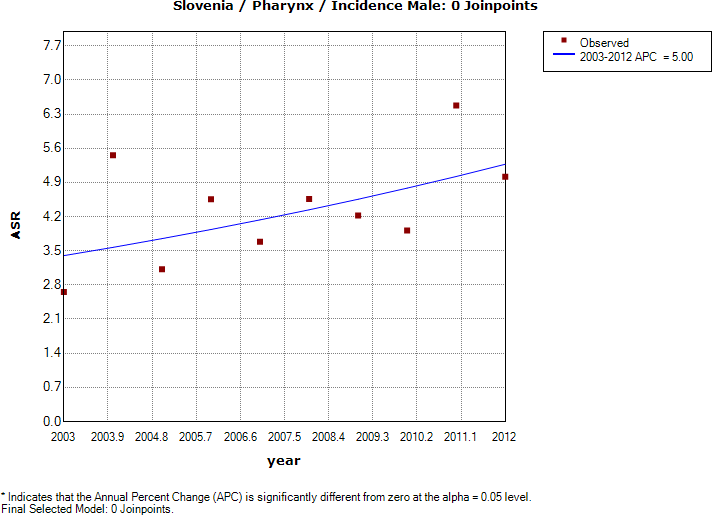 | 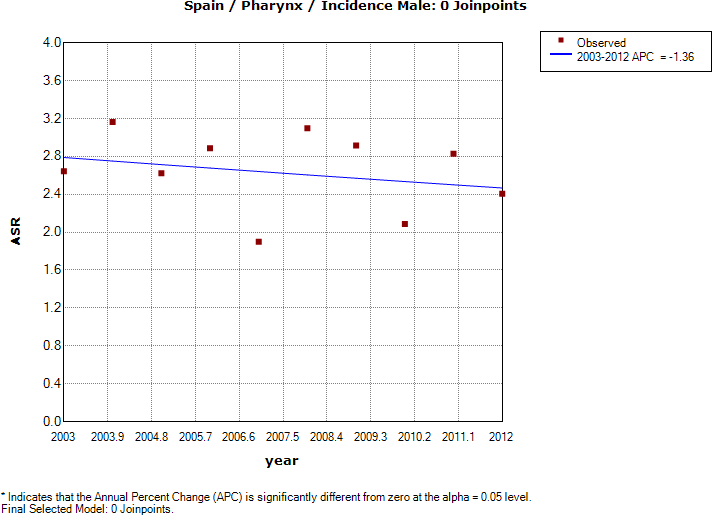 |

| **Eastern Europe** | |
| --- | --- |
| 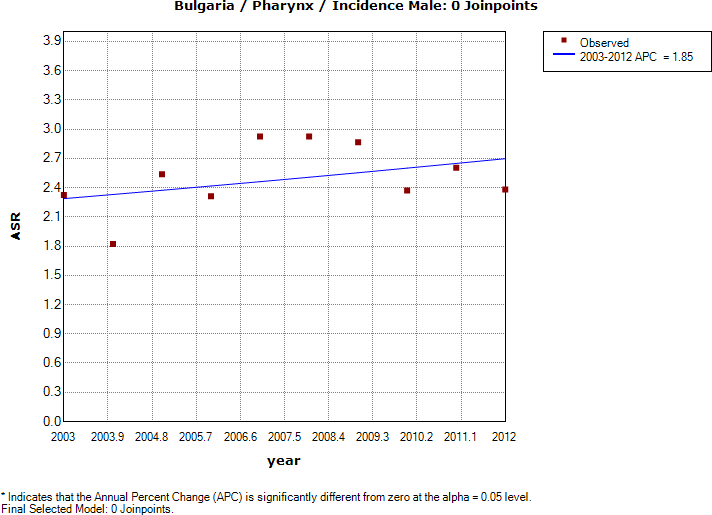 | 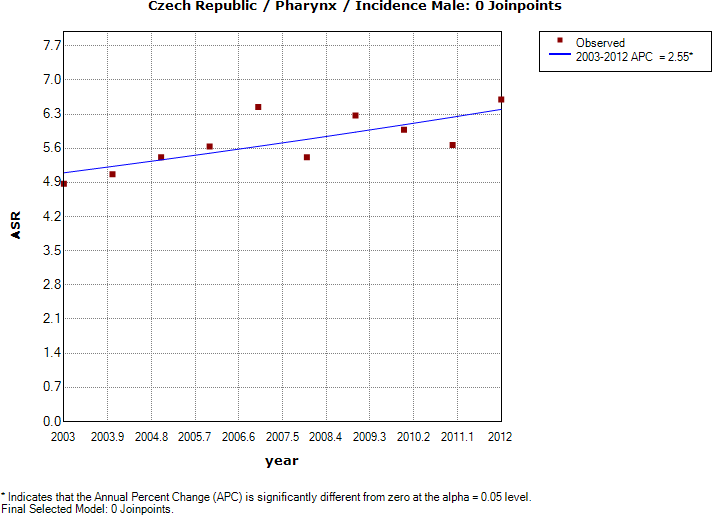 |
| 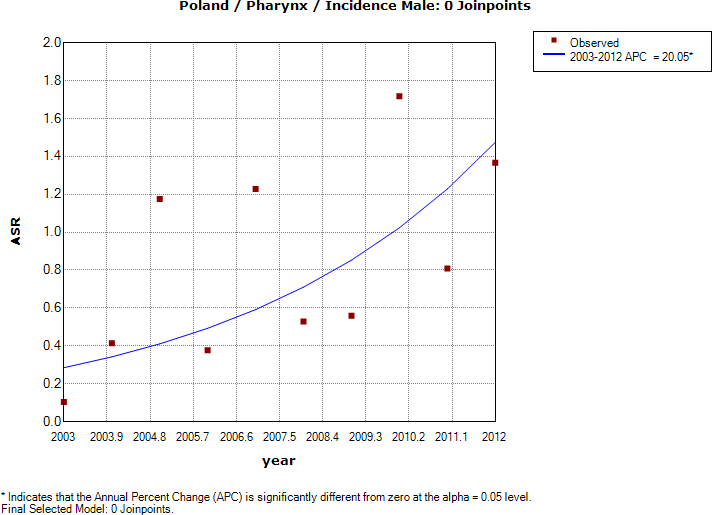 |  |
| **Africa** | |
| 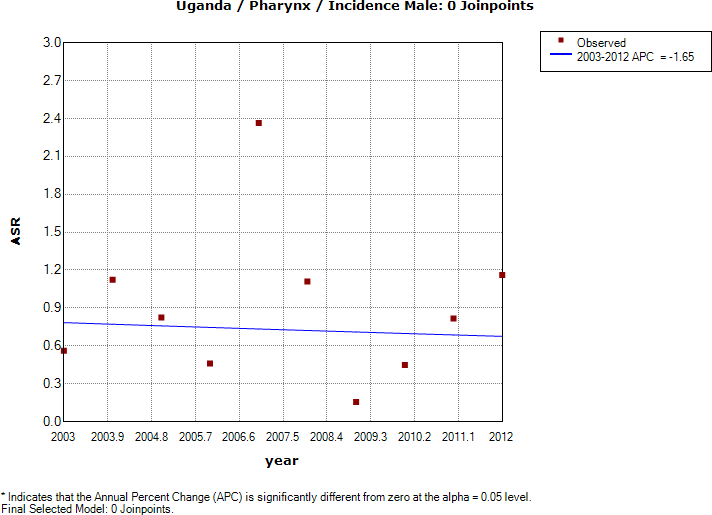 |  |

1. Pharynx - female

| **Asia** | |
| --- | --- |
| 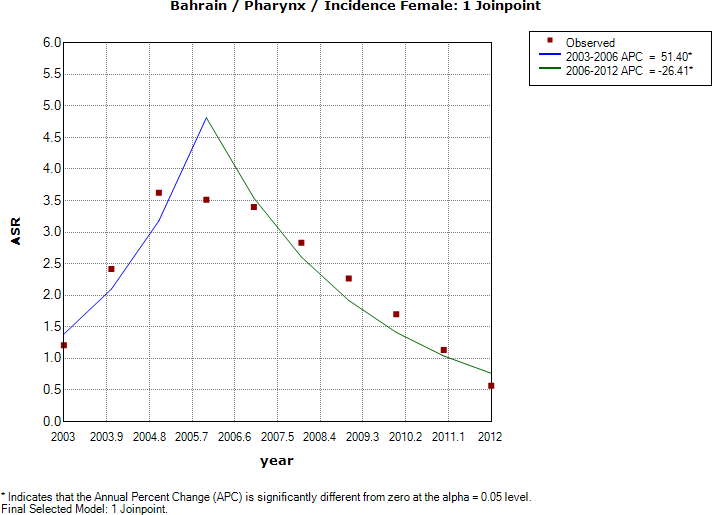 | 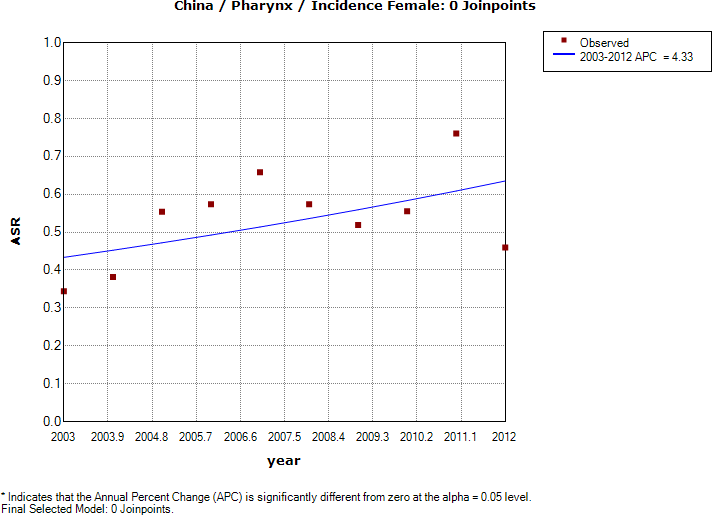 |
| 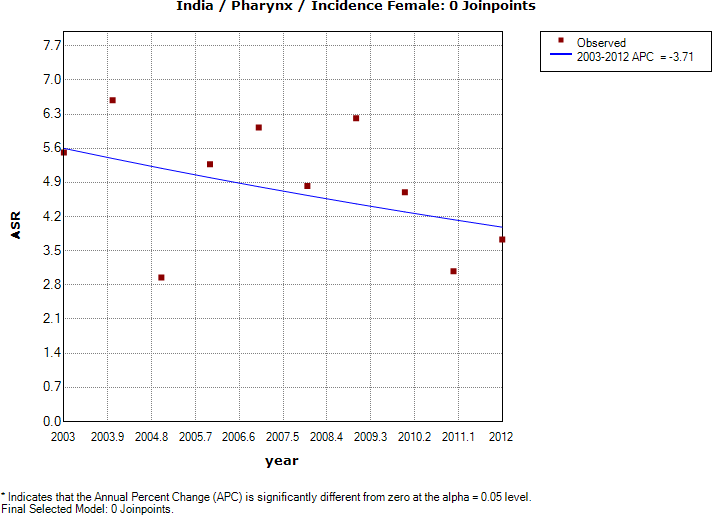 | 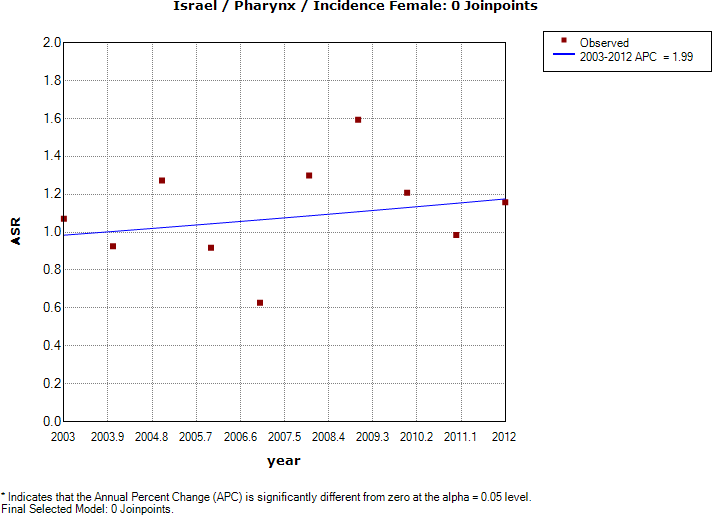 |
| 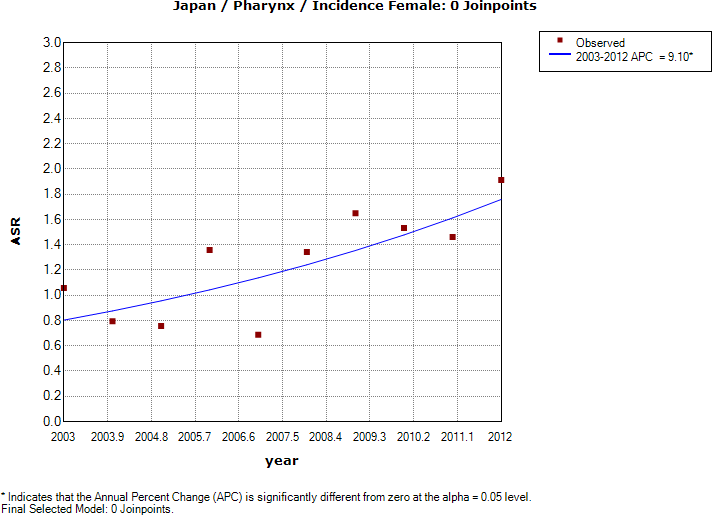 | 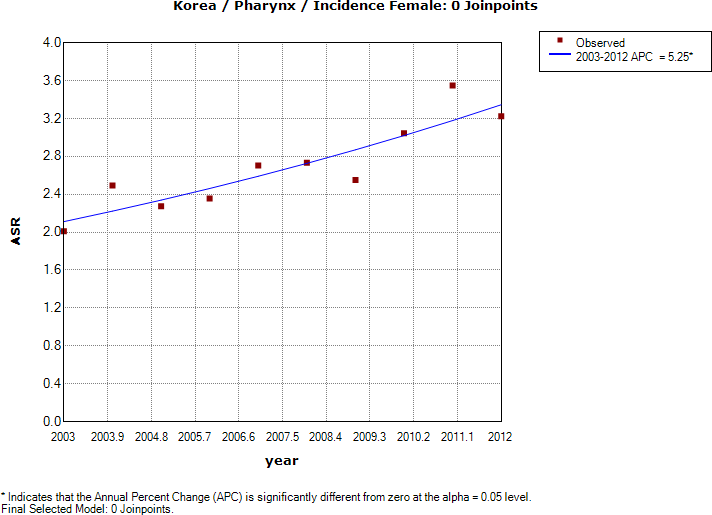 |

| 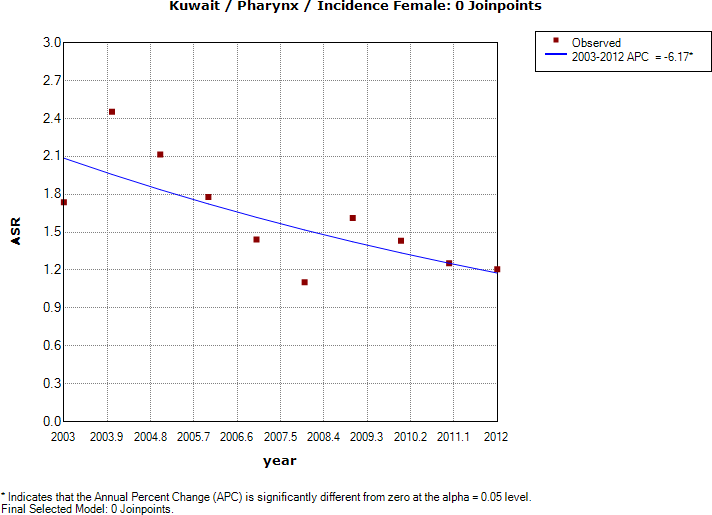 | 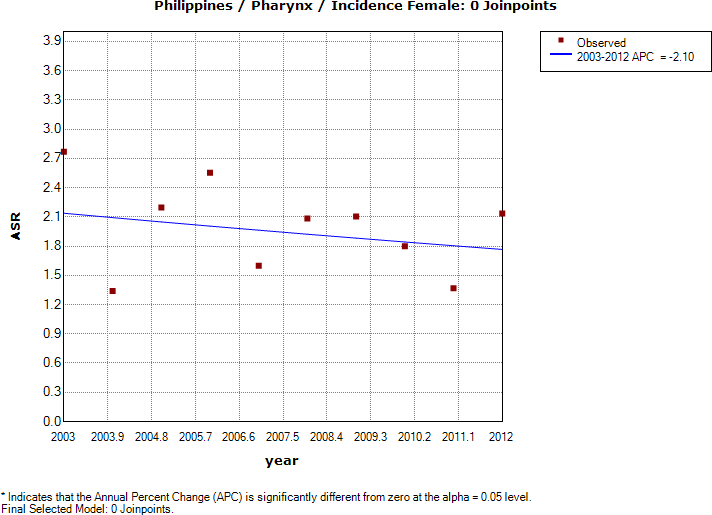 |
| --- | --- |
| 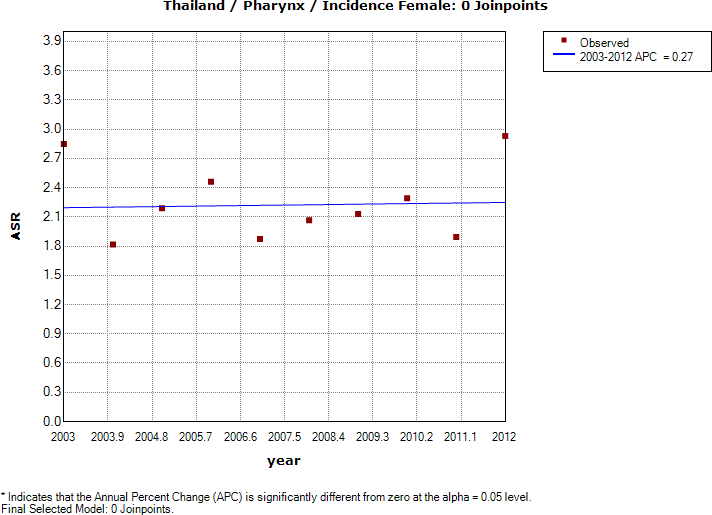 | 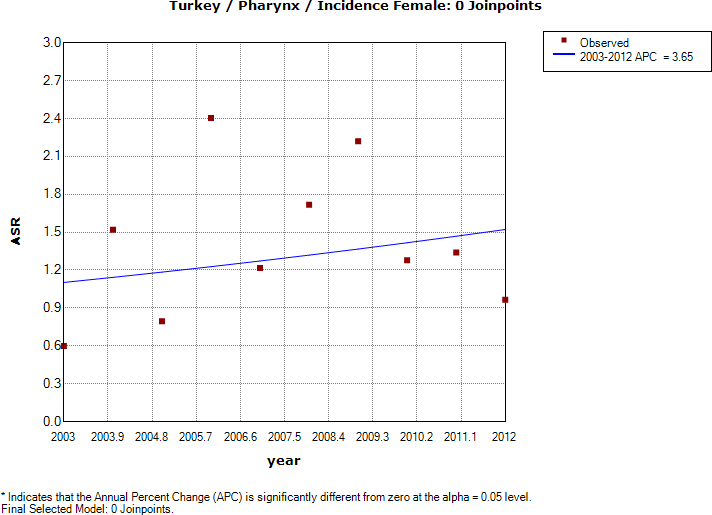 |
| **Oceania** | |
| 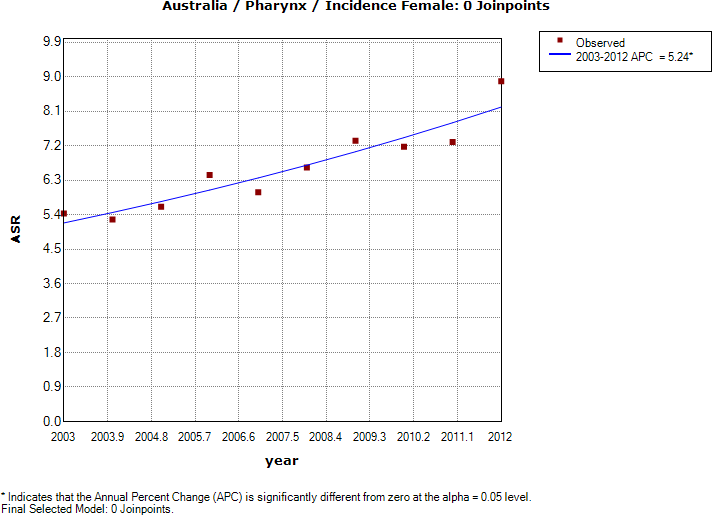 | 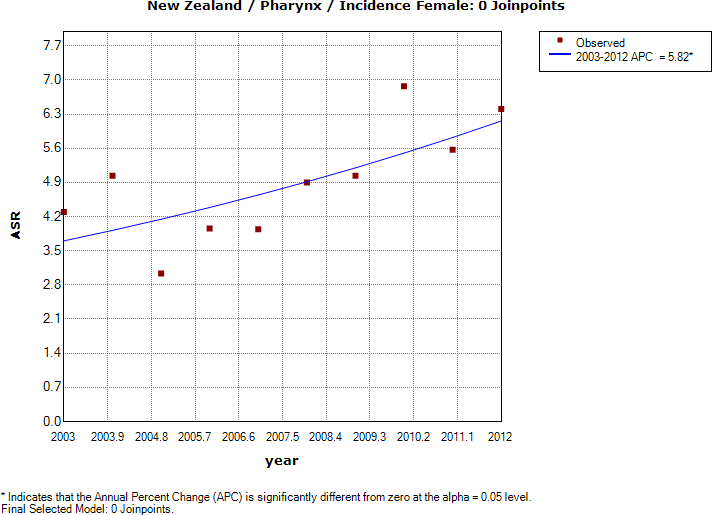 |

| **Northern America** | |
| --- | --- |
| 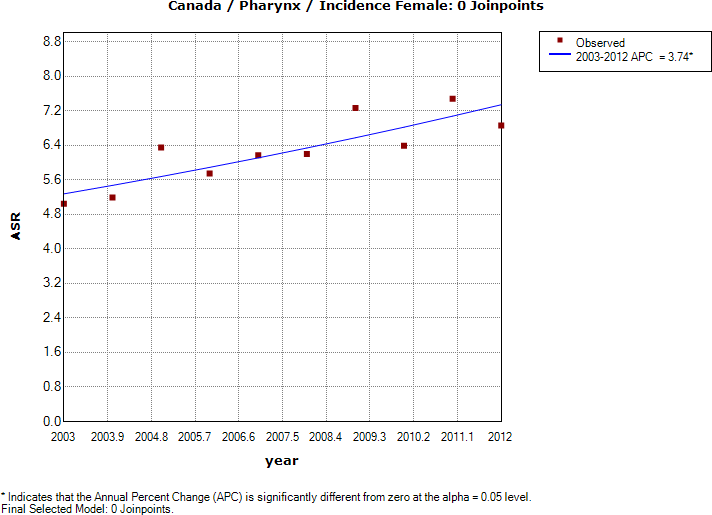 | 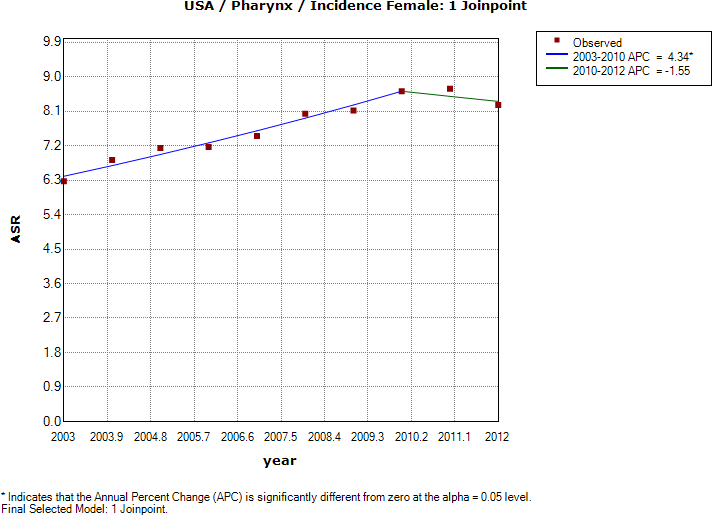 |
| **Southern America** | |
| 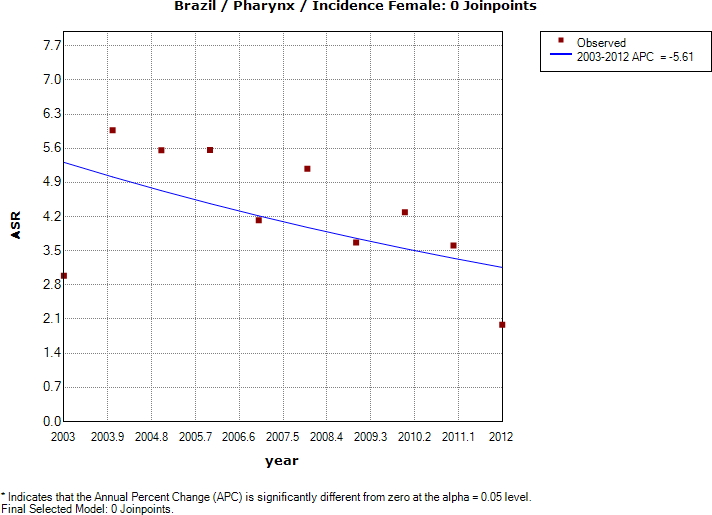 | 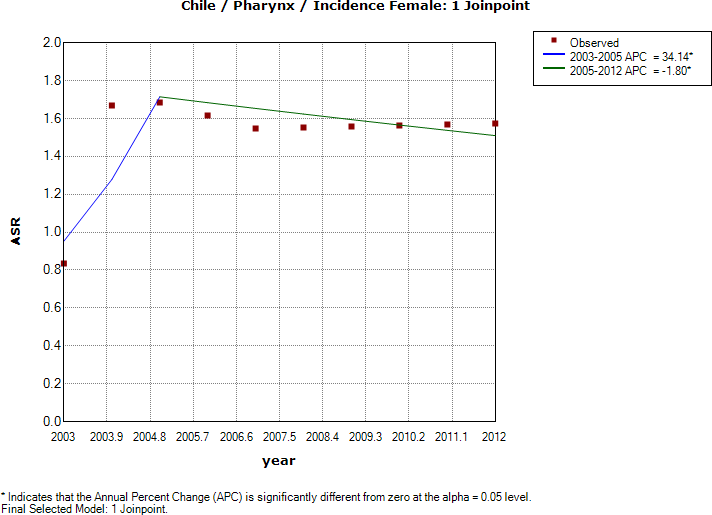 |
| 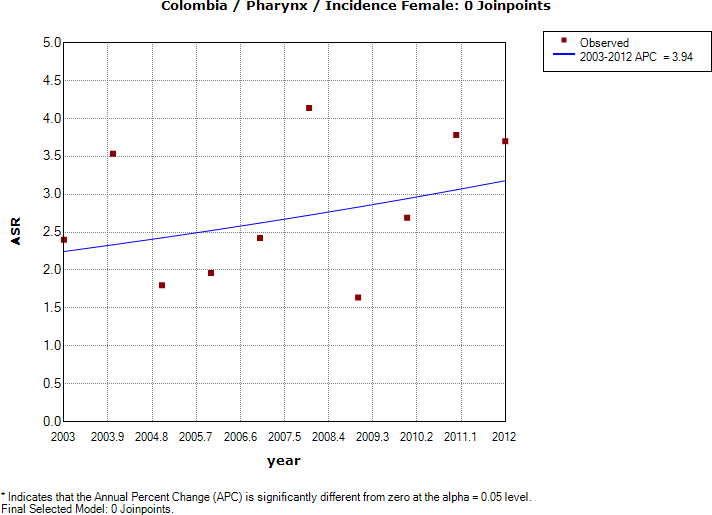 | 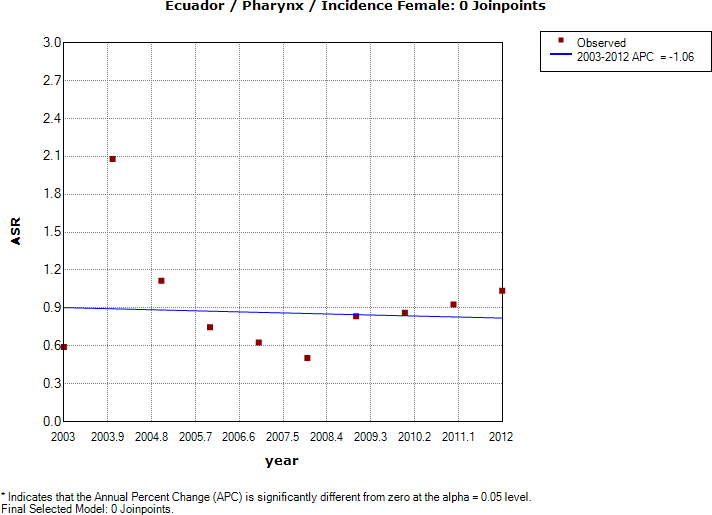 |

| 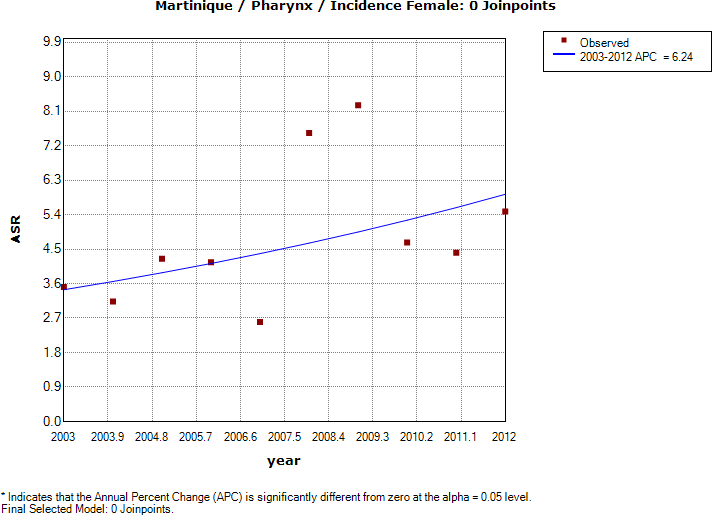 |  |
| --- | --- |
| **Northern Europe** | |
| 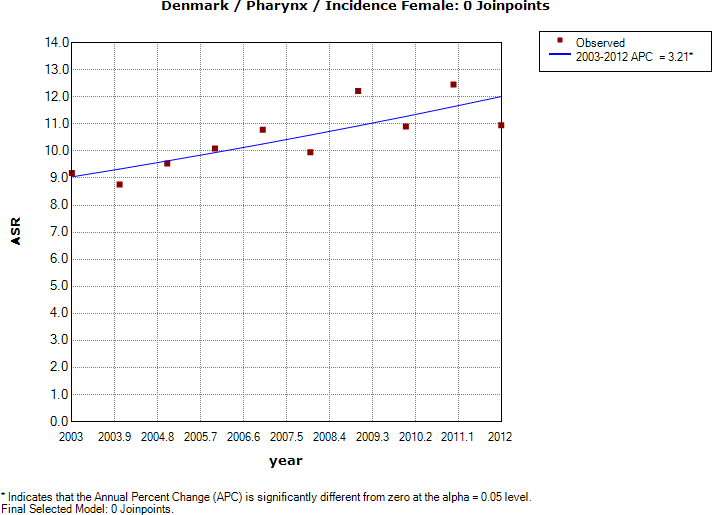 | 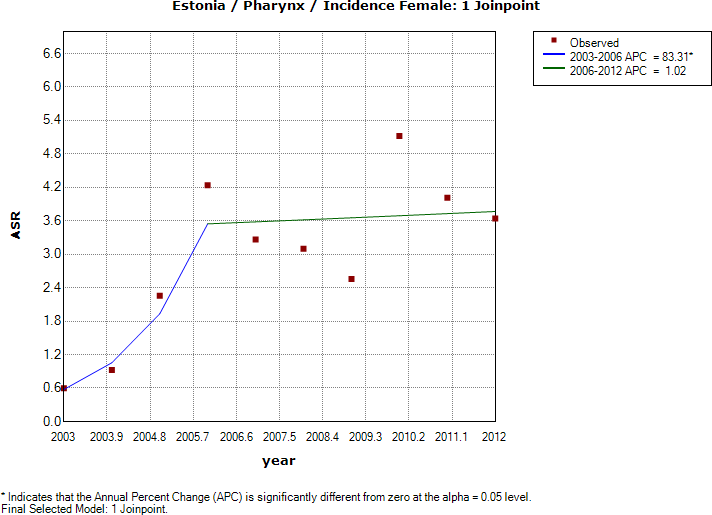 |
| 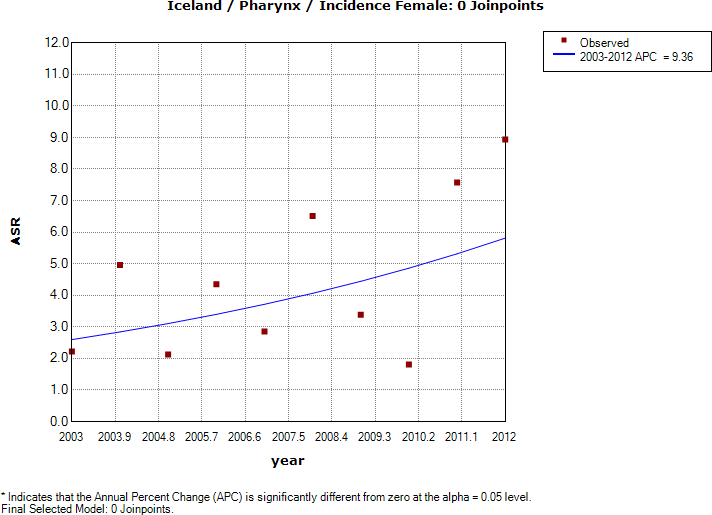 | 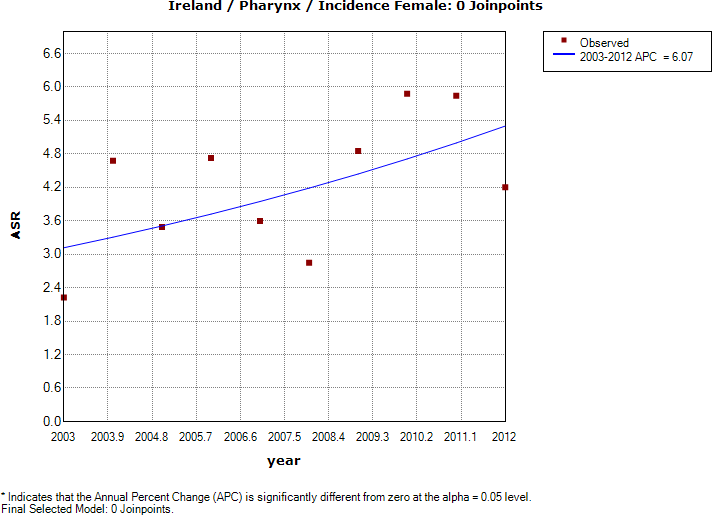 |
| 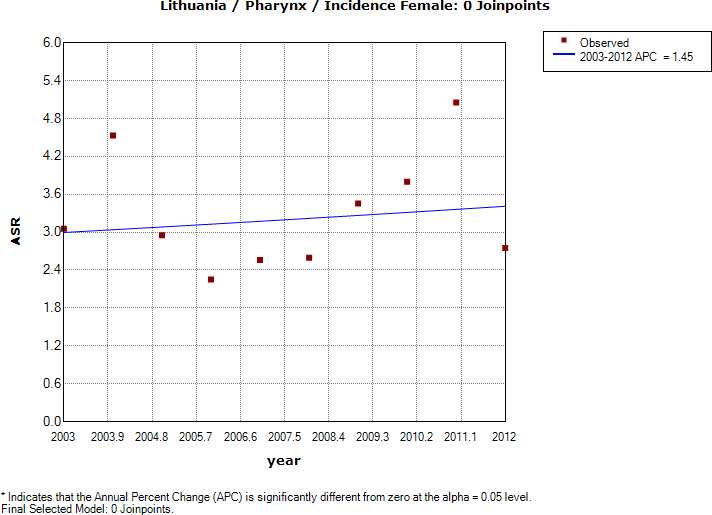 | 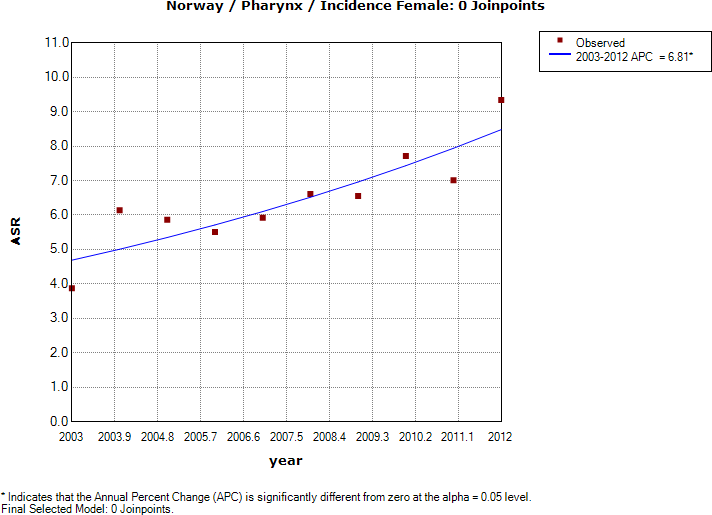 |

| 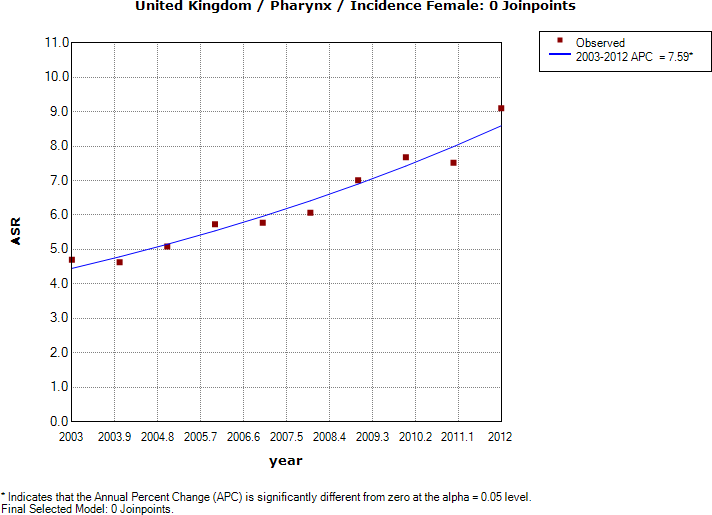 |  |
| --- | --- |
| **Western Europe** | |
| 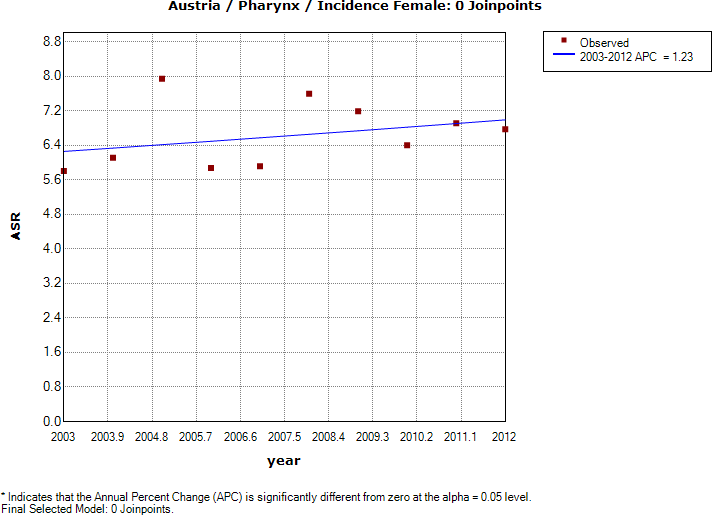 | 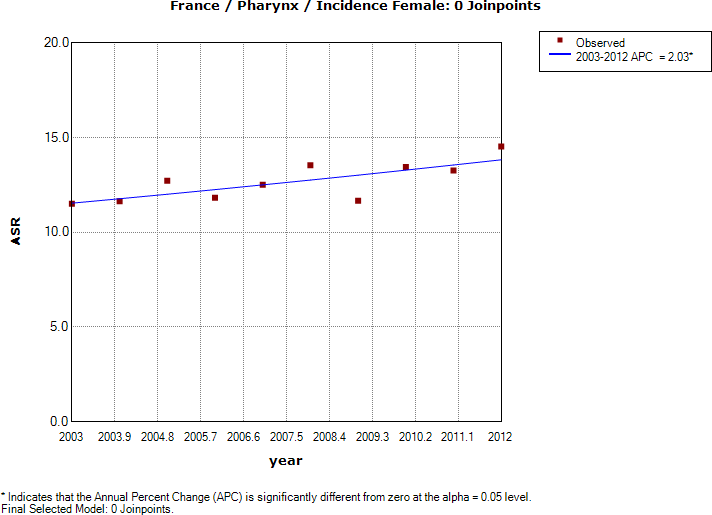 |
| 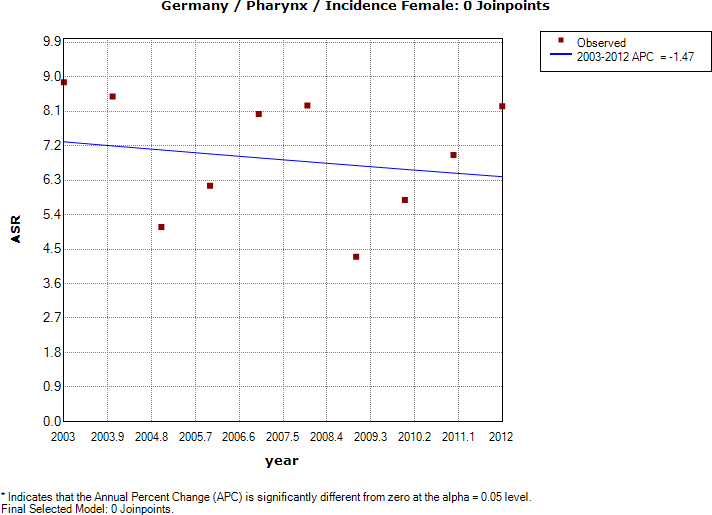 | 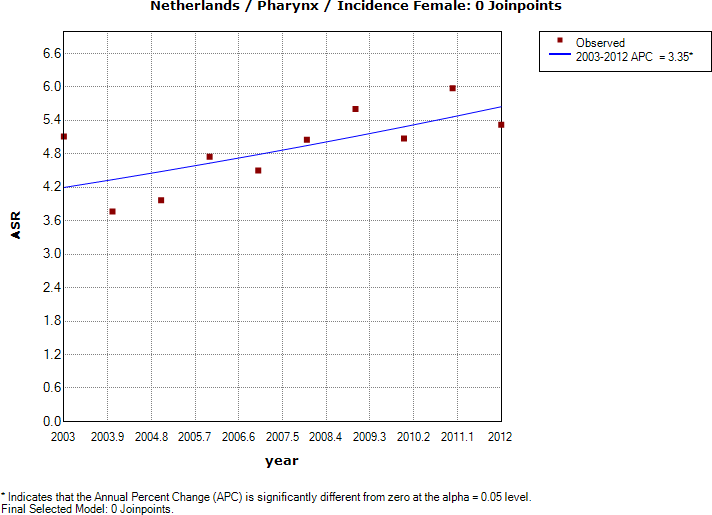 |
| 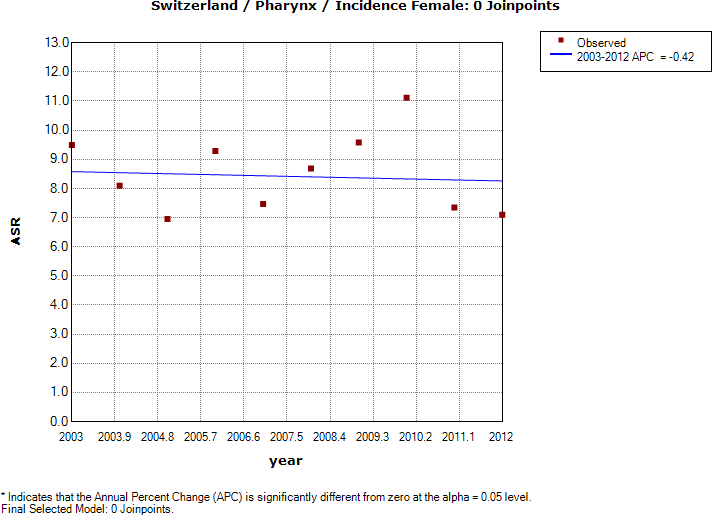 |  |

| **Southern Europe** | |
| --- | --- |
| 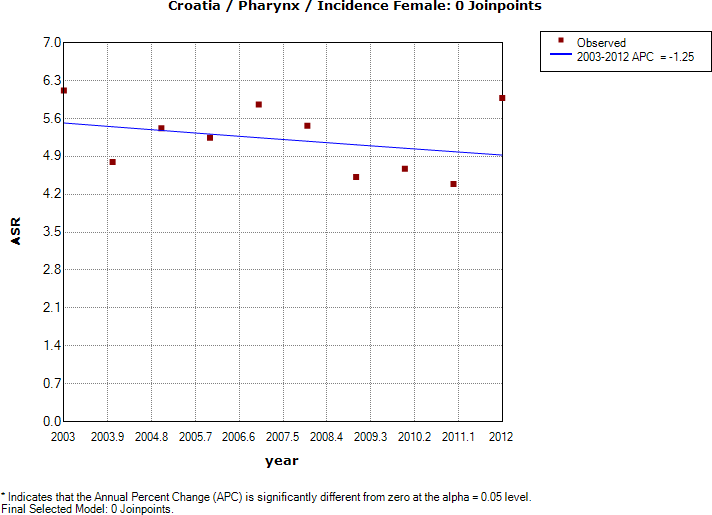 | 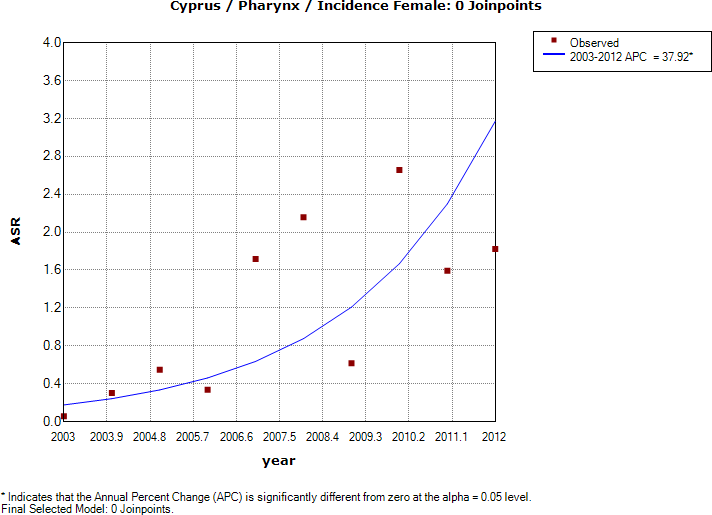 |
| 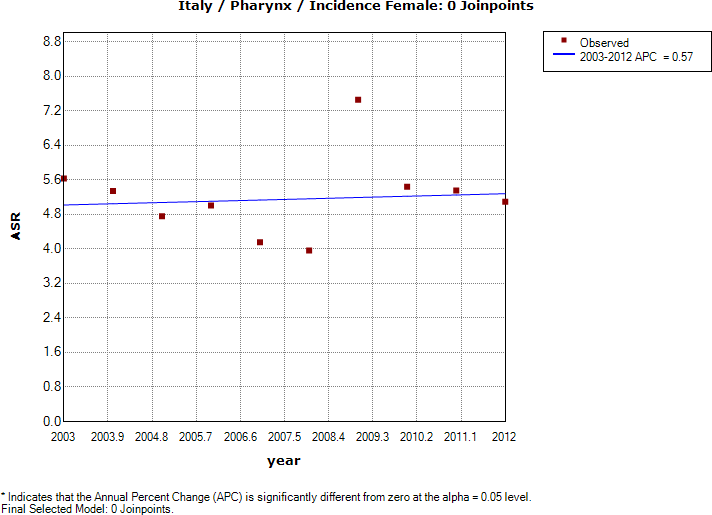 | 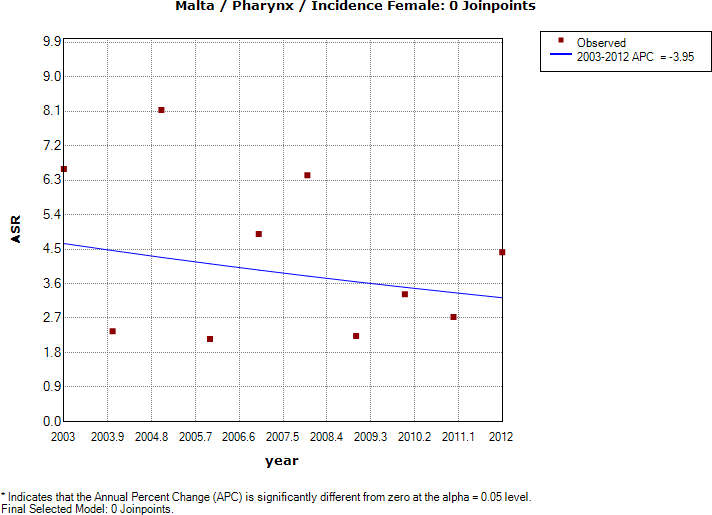 |
| 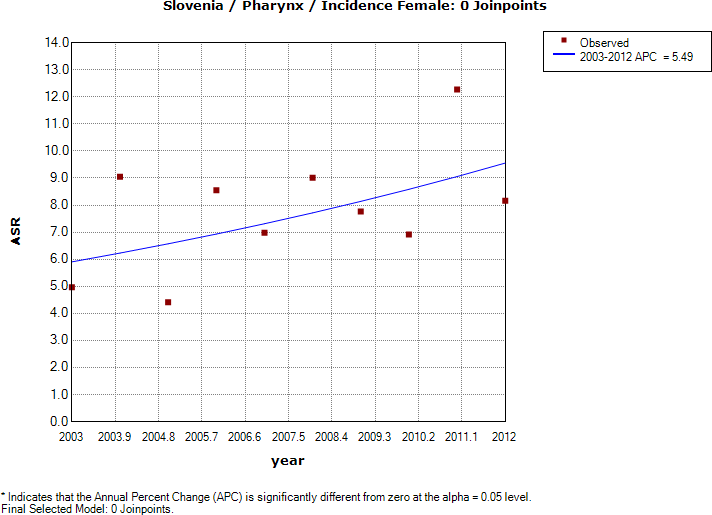 | 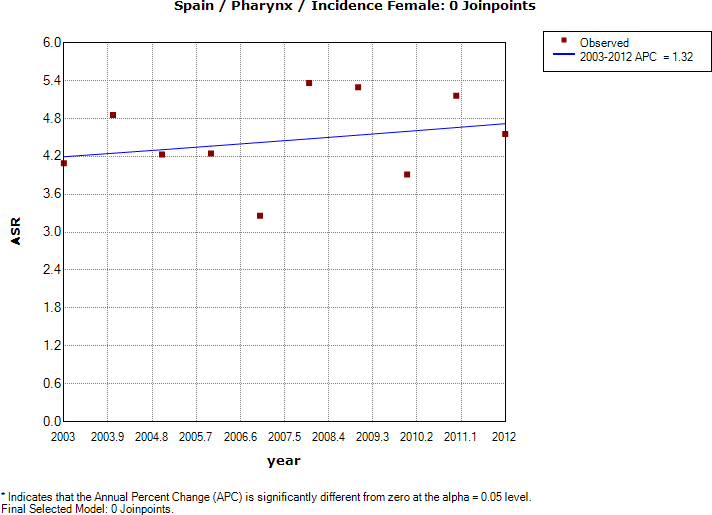 |

| **Eastern Europe** | |
| --- | --- |
| 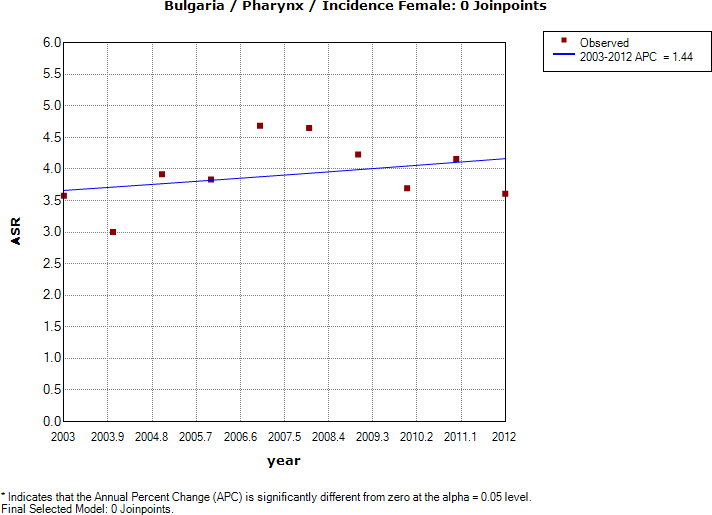 | 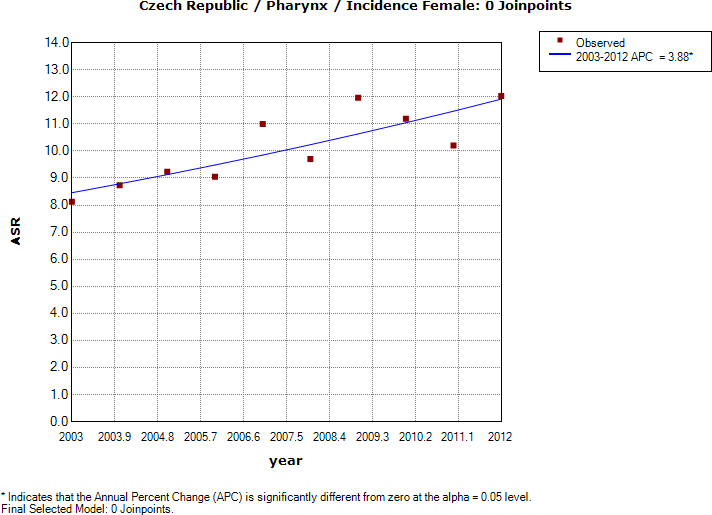 |
| 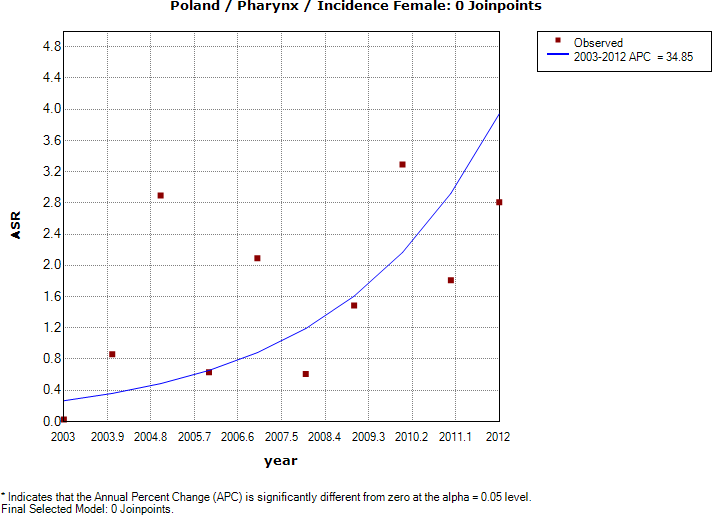 |  |
| **Africa** | |
| 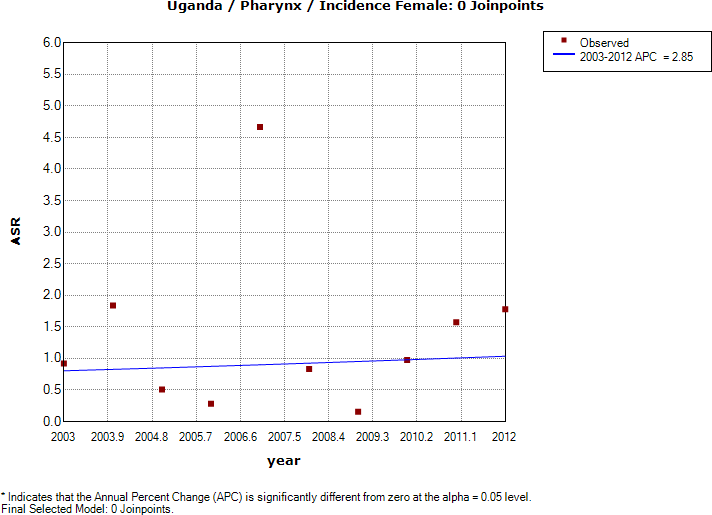 |  |

1. Pharynx - both

| **Asia** | |
| --- | --- |
| 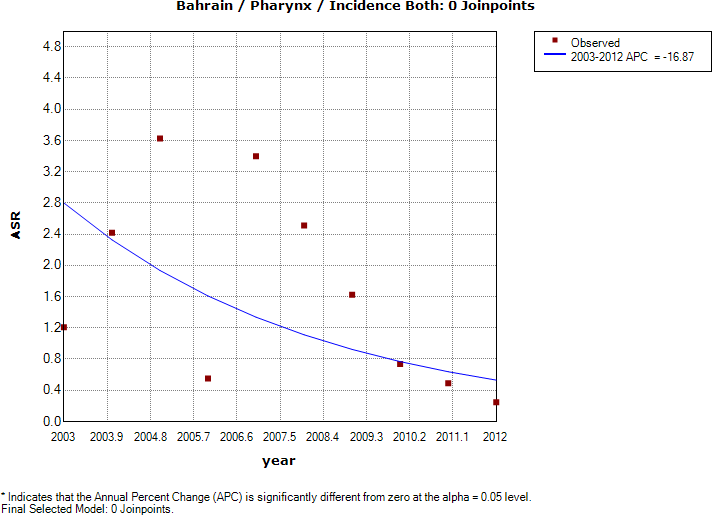 | 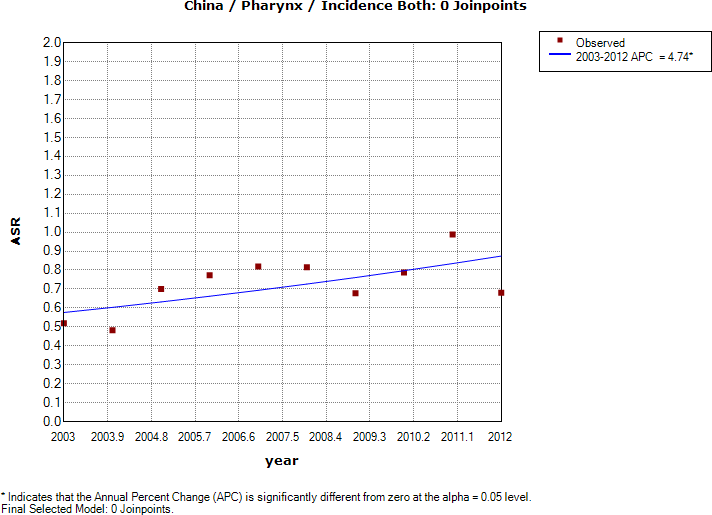 |
| 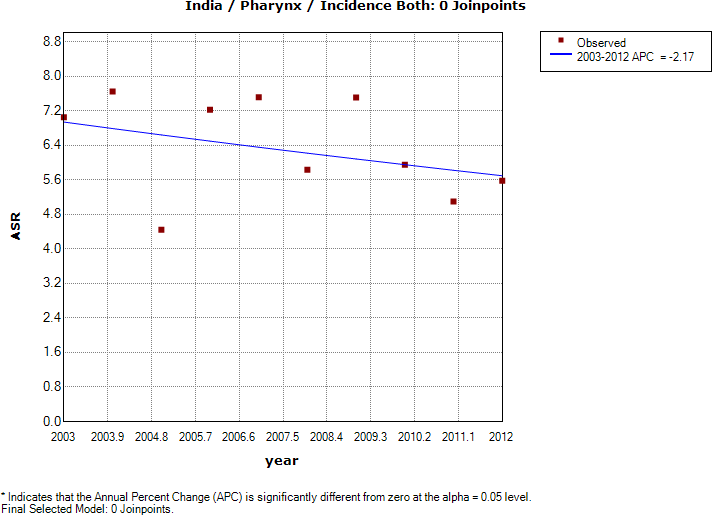 | 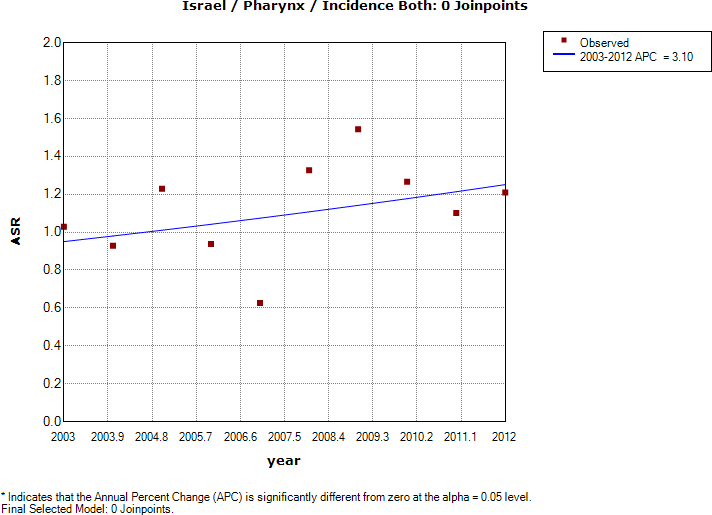 |
| 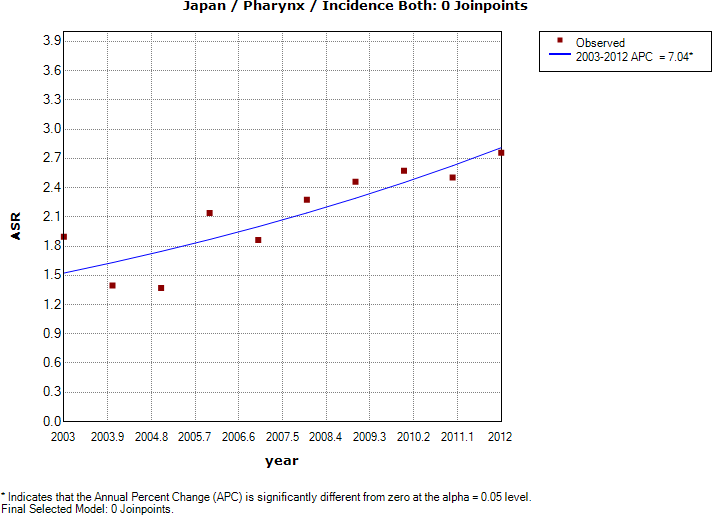 | 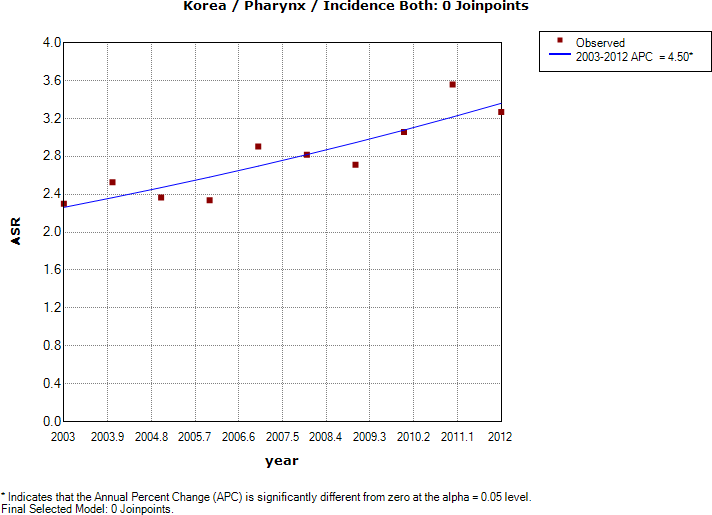 |

| 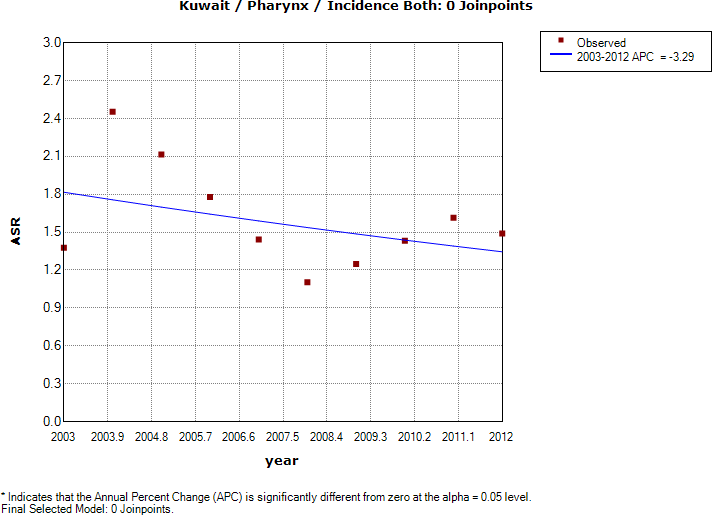 | 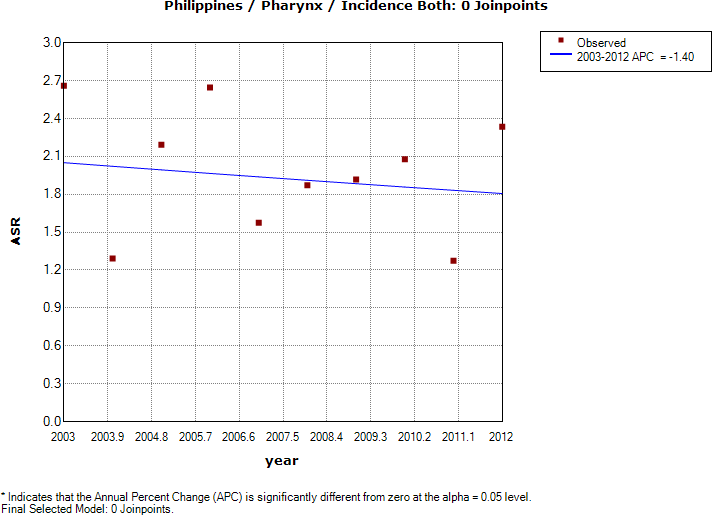 |
| --- | --- |
| 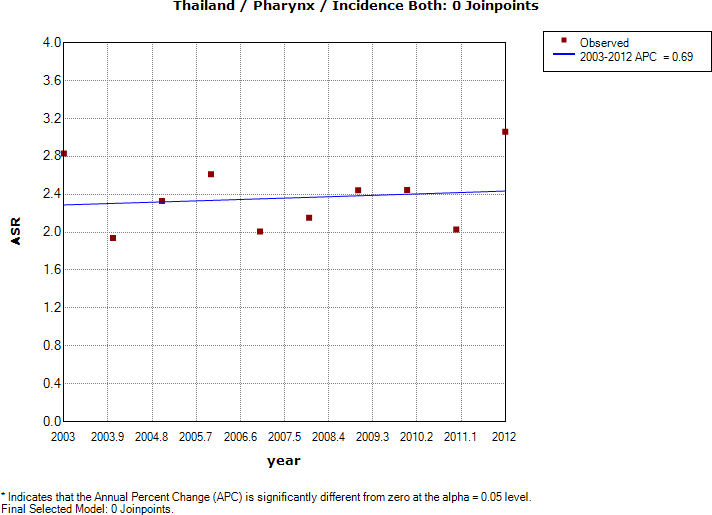 | 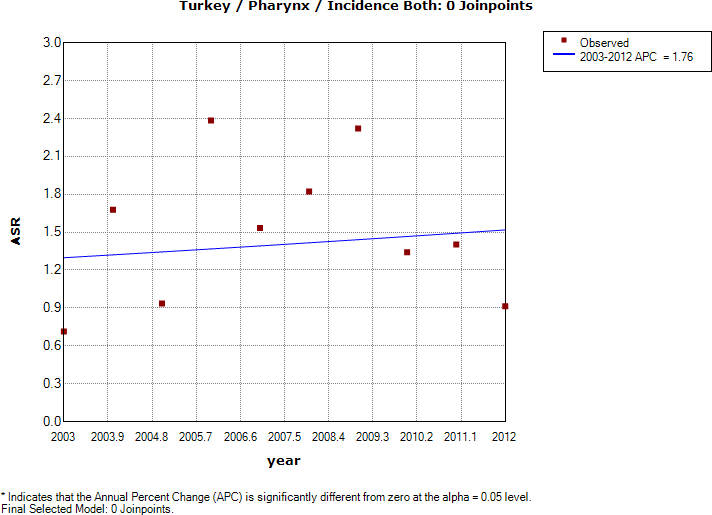 |
| **Oceania** | |
| 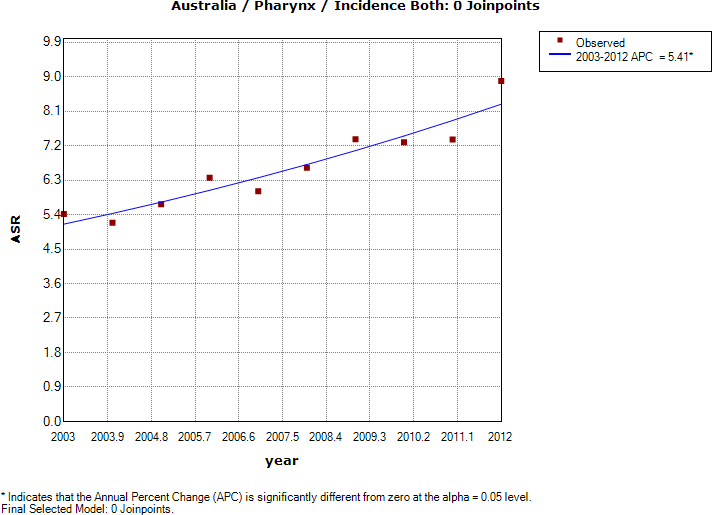 | 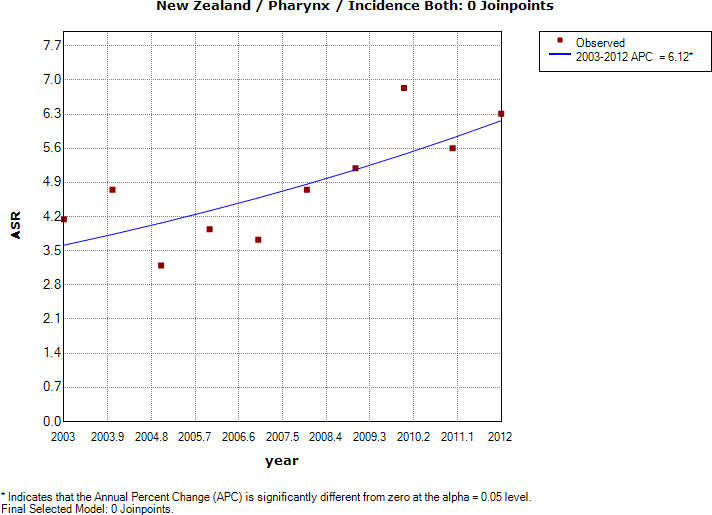 |

| **Northern America** | |
| --- | --- |
| 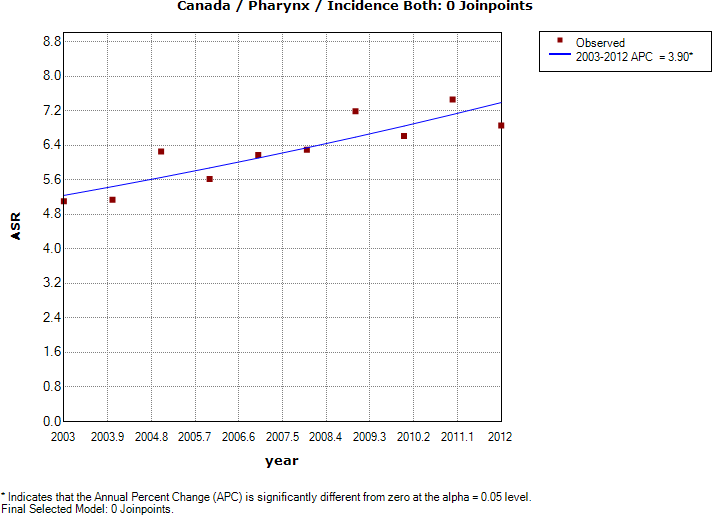 | 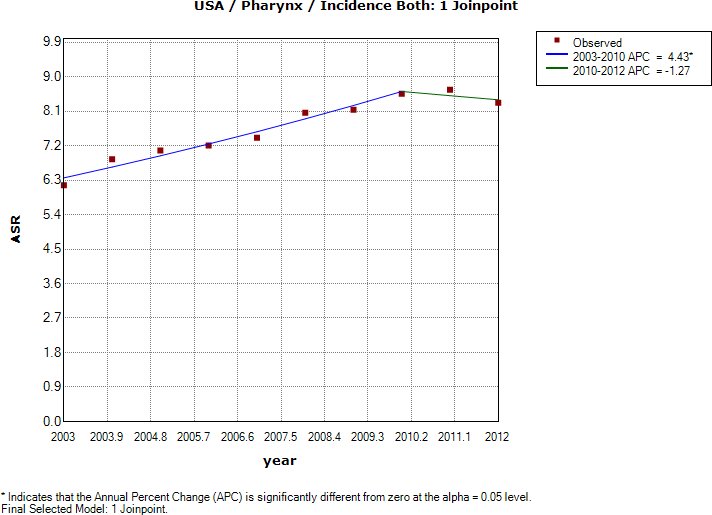 |
| **Southern America** | |
| 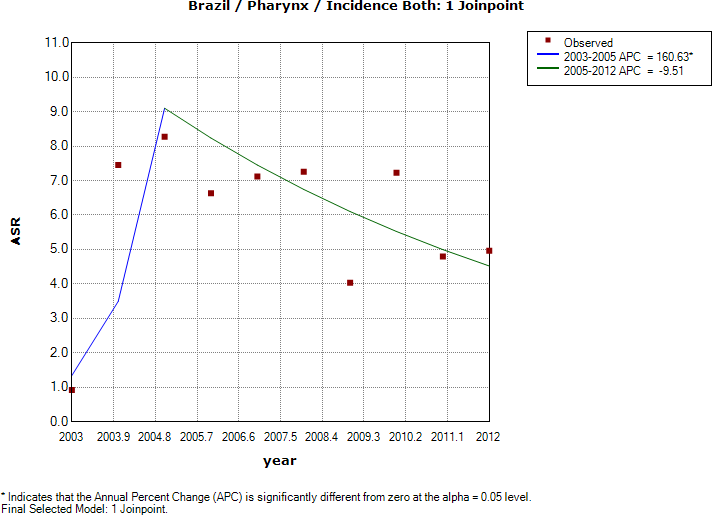 | 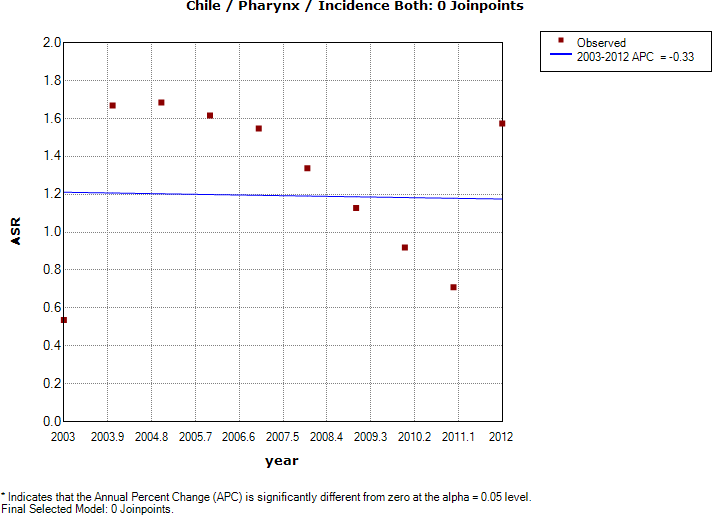 |
| 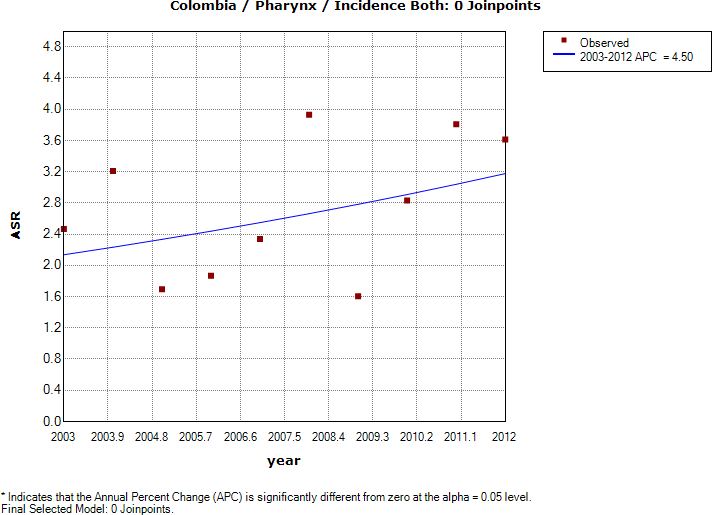 | 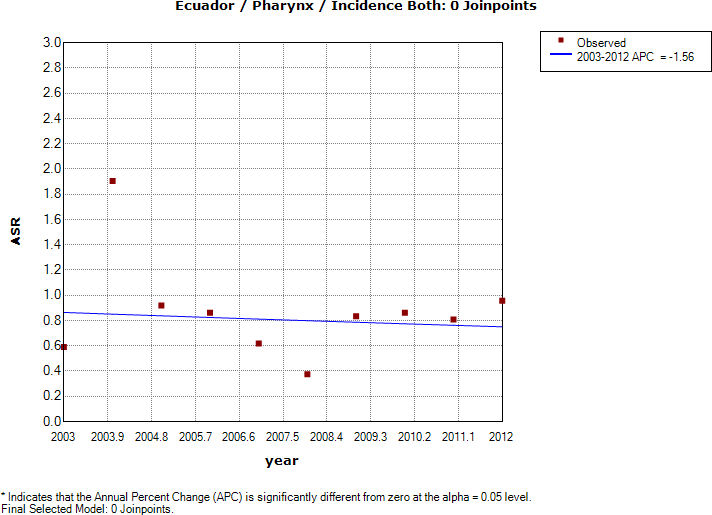 |

| 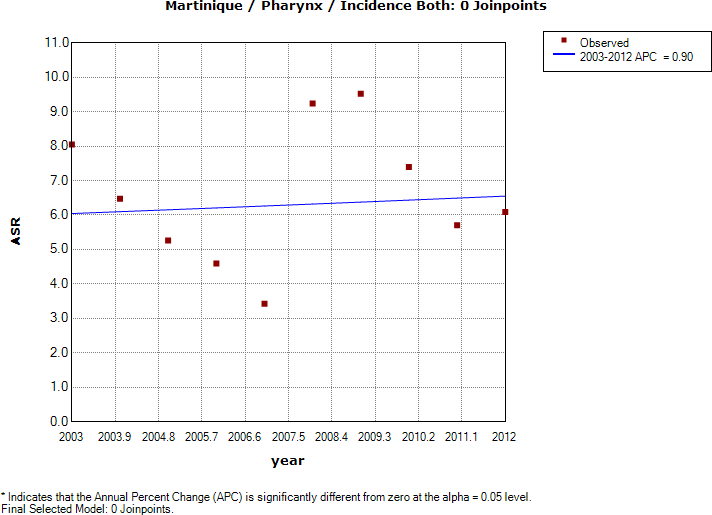 |  |
| --- | --- |
| **Northern Europe** | |
|  |  |
|  |  |
|  |  |

|  |  |
| --- | --- |
| **Western Europe** | |
|  |  |
|  |  |
|  |  |

| **Southern Europe** | |
| --- | --- |
|  |  |
|  |  |
|  |  |

| **Eastern Europe** | |
| --- | --- |
|  |  |
|  |  |
| **Africa** | |
|  |  |

1. Pharynx - young

| **Asia** | |
| --- | --- |
|  |  |
|  |  |
|  |  |

|  |  |
| --- | --- |
|  |  |
| **Oceania** | |
|  |  |

| **Northern America** | |
| --- | --- |
|  |  |
| **Southern America** | |
|  |  |
|  |  |

|  |  |
| --- | --- |
| **Northern Europe** | |
|  |  |
|  |  |
|  |  |

|  |  |
| --- | --- |
| **Western Europe** | |
|  |  |
|  |  |
|  |  |

| **Southern Europe** | |
| --- | --- |
|  |  |
|  |  |
|  |  |

| **Eastern Europe** | |
| --- | --- |
|  |  |
|  |  |
| **Africa** | |
|  |  |

1. Pharynx - old

| **Asia** | |
| --- | --- |
|  |  |
|  |  |
|  |  |

|  |  |
| --- | --- |
|  |  |
| **Oceania** | |
|  |  |

| **Northern America** | |
| --- | --- |
|  |  |
| **Southern America** | |
|  |  |
|  |  |

|  |  |
| --- | --- |
| **Northern Europe** | |
|  |  |
|  |  |
|  |  |

|  |  |
| --- | --- |
| **Western Europe** | |
|  |  |
|  |  |
|  |  |

| **Southern Europe** | |
| --- | --- |
|  |  |
|  |  |
|  |  |

| **Eastern Europe** | |
| --- | --- |
|  |  |
|  |  |
| **Africa** | |
|  |  |

1. Oropharynx - Male

| **Asia** | |
| --- | --- |
|  |  |
|  |  |
|  |  |

|  |  |
| --- | --- |
|  |  |
| **Oceania** | |
|  |  |

| **Northern America** | |
| --- | --- |
|  |  |
| **Southern America** | |
|  |  |
|  |  |

|  |  |
| --- | --- |
| **Northern Europe** | |
|  |  |
|  |  |
|  |  |

|  |  |
| --- | --- |
| **Western Europe** | |
|  |  |
|  |  |
|  |  |

| **Southern Europe** | |
| --- | --- |
|  |  |
|  |  |
|  |  |

| **Eastern Europe** | |
| --- | --- |
|  |  |
|  |  |
| **Africa** | |
|  |  |

1. Oropharynx – female

| **Asia** | |
| --- | --- |
|  |  |
|  |  |
|  |  |

|  |  |
| --- | --- |
|  |  |
| **Oceania** | |
|  |  |

| **Northern America** | |
| --- | --- |
|  |  |
| **Southern America** | |
|  |  |
|  |  |

| **Northern Europe** | |
| --- | --- |
|  |  |
|  |  |
|  |  |
|  |  |

| **Western Europe** | |
| --- | --- |
|  |  |
|  |  |
|  |  |

| **Southern Europe** | |
| --- | --- |
|  |  |
|  |  |
|  |  |

| **Eastern Europe** | |
| --- | --- |
|  |  |
|  |  |
| **Africa** | |
|  |  |

1. Oropharynx - both

| **Asia** | |
| --- | --- |
|  |  |
|  |  |
|  |  |

|  |  |
| --- | --- |
|  |  |
| **Oceania** | |
|  |  |

| **Northern America** | |
| --- | --- |
|  |  |
| **Southern America** | |
|  |  |
|  |  |

|  |  |
| --- | --- |
| **Northern Europe** | |
|  |  |
|  |  |
|  |  |

|  |  |
| --- | --- |
| **Western Europe** | |
|  |  |
|  |  |
|  |  |

| **Southern Europe** | |
| --- | --- |
|  |  |
|  |  |
|  |  |

| **Eastern Europe** | |
| --- | --- |
|  |  |
|  |  |
| **Africa** | |
|  |  |

1. Oropharynx - young

| **Asia** | |
| --- | --- |
|  |  |
|  |  |
|  |  |

|  |  |
| --- | --- |
|  |  |
| **Oceania** | |
|  |  |

| **Northern America** | |
| --- | --- |
|  |  |
| **Southern America** | |
|  |  |
|  |  |

| **Northern Europe** | |
| --- | --- |
|  |  |
|  |  |
|  |  |
|  |  |

| **Western Europe** | |
| --- | --- |
|  |  |
|  |  |
|  |  |

| **Southern Europe** | |
| --- | --- |
|  |  |
|  |  |
|  |  |

| **Eastern Europe** | |
| --- | --- |
|  |  |
|  |  |
| **Africa** | |
|  |  |

1. Oropharynx - old

| **Asia** | |
| --- | --- |
|  |  |
|  |  |
|  |  |

|  |  |
| --- | --- |
|  |  |
| **Oceania** | |
|  |  |

| **Northern America** | |
| --- | --- |
|  |  |
| **Southern America** | |
|  |  |
|  |  |

|  |  |
| --- | --- |
| **Northern Europe** | |
|  |  |
|  |  |
|  |  |

|  |  |
| --- | --- |
| **Western Europe** | |
|  |  |
|  |  |
|  |  |

| **Southern Europe** | |
| --- | --- |
|  |  |
|  |  |
|  |  |

| **Eastern Europe** | |
| --- | --- |
|  |  |
|  |  |
| **Africa** | |
|  |  |

1. Nasopharynx - Male

| **Asia** | |
| --- | --- |
|  |  |
|  |  |
|  |  |

|  |  |
| --- | --- |
|  |  |
| **Oceania** | |
|  |  |

| **Northern America** | |
| --- | --- |
|  |  |
| **Southern America** | |
|  |  |
|  |  |

| **Northern Europe** | |
| --- | --- |
|  |  |
|  |  |
|  |  |
|  |  |

| **Western Europe** | |
| --- | --- |
|  |  |
|  |  |
|  |  |

| **Southern Europe** | |
| --- | --- |
|  |  |
|  |  |
|  |  |

| **Eastern Europe** | |
| --- | --- |
|  |  |
|  |  |
| **Africa** | |
|  |  |

1. Nasopharynx - female

| **Asia** | |
| --- | --- |
|  |  |
|  |  |
|  |  |

|  |  |
| --- | --- |
|  |  |
| **Oceania** | |
|  |  |

| **Northern America** | |
| --- | --- |
|  |  |
| **Southern America** | |
|  |  |
|  |  |

| **Northern Europe** | |
| --- | --- |
|  |  |
|  |  |
|  |  |
|  |  |

| **Western Europe** | |
| --- | --- |
|  |  |
|  |  |
|  |  |

| **Southern Europe** | |
| --- | --- |
|  |  |
|  |  |
|  |  |

| **Eastern Europe** | |
| --- | --- |
|  |  |
|  |  |
| **Africa** | |
|  |  |

1. Nasopharynx - both

| **Asia** | |
| --- | --- |
|  |  |
|  |  |
|  |  |

|  |  |
| --- | --- |
|  |  |
| **Oceania** | |
|  |  |

| **Northern America** | |
| --- | --- |
|  |  |
| **Southern America** | |
|  |  |
|  |  |

| **Northern Europe** | |
| --- | --- |
|  |  |
|  |  |
|  |  |
|  |  |

| **Western Europe** | |
| --- | --- |
|  |  |
|  |  |
|  |  |

| **Southern Europe** | |
| --- | --- |
|  |  |
|  |  |
|  |  |

| **Eastern Europe** | |
| --- | --- |
|  |  |
|  |  |
| **Africa** | |
|  |  |

1. Nasopharynx - young

| **Asia** | |
| --- | --- |
|  |  |
|  |  |
|  |  |

|  |  |
| --- | --- |
|  |  |
| **Oceania** | |
|  |  |

| **Northern America** | |
| --- | --- |
|  |  |
| **Southern America** | |
|  |  |
|  |  |

| **Northern Europe** | |
| --- | --- |
|  |  |
|  |  |
|  |  |
|  |  |

| **Western Europe** | |
| --- | --- |
|  |  |
|  |  |
|  |  |

| **Southern Europe** | |
| --- | --- |
|  |  |
|  |  |
|  |  |

| **Eastern Europe** | |
| --- | --- |
|  |  |
|  |  |
| **Africa** | |
|  |  |

1. Nasopharynx - old

| **Asia** | |
| --- | --- |
|  |  |
|  |  |
|  |  |

|  |  |
| --- | --- |
|  |  |
| **Oceania** | |
|  |  |

| **Northern America** | |
| --- | --- |
|  |  |
| **Southern America** | |
|  |  |
|  |  |

| **Northern Europe** | |
| --- | --- |
|  |  |
|  |  |
|  |  |
|  |  |

| **Western Europe** | |
| --- | --- |
|  |  |
|  |  |
|  |  |

| **Southern Europe** | |
| --- | --- |
|  |  |
|  |  |
|  |  |

| **Eastern Europe** | |
| --- | --- |
|  |  |
|  |  |
| **Africa** | |
|  |  |

1. Hypopharynx - Male

| **Asia** | |
| --- | --- |
|  |  |
|  |  |
|  |  |

|  |  |
| --- | --- |
|  |  |
| **Oceania** | |
|  |  |

| **Northern America** | |
| --- | --- |
|  |  |
| **Southern America** | |
|  |  |
|  |  |

| **Northern Europe** | |
| --- | --- |
|  |  |
|  |  |
|  |  |
|  |  |

| **Western Europe** | |
| --- | --- |
|  |  |
|  |  |
|  |  |

| **Southern Europe** | |
| --- | --- |
|  |  |
|  |  |
|  |  |

| **Eastern Europe** | |
| --- | --- |
|  |  |
|  |  |
| **Africa** | |
|  |  |

1. Hypopharynx - female

| **Asia** | |
| --- | --- |
|  |  |
|  |  |
|  |  |

|  |  |
| --- | --- |
|  |  |
| **Oceania** | |
|  |  |

| **Northern America** | |
| --- | --- |
|  |  |
| **Southern America** | |
|  |  |
|  |  |

|  |  |
| --- | --- |
| **Northern Europe** | |
|  |  |
|  |  |
|  |  |

| **Western Europe** | |
| --- | --- |
|  |  |
|  |  |
|  |  |

| **Southern Europe** | |
| --- | --- |
|  |  |
|  |  |
|  |  |

| **Eastern Europe** | |
| --- | --- |
|  |  |
|  |  |
| **Africa** | |
|  |  |

1. Hypopharynx - both

| **Asia** | |
| --- | --- |
|  |  |
|  |  |
|  |  |

|  |  |
| --- | --- |
|  |  |
| **Oceania** | |
|  |  |

| **Northern America** | |
| --- | --- |
|  |  |
| **Southern America** | |
|  |  |
|  |  |

|  |  |
| --- | --- |
| **Northern Europe** | |
|  |  |
|  |  |
|  |  |

|  |  |
| --- | --- |
| **Western Europe** | |
|  |  |
|  |  |
|  |  |

| **Southern Europe** | |
| --- | --- |
|  |  |
|  |  |
|  |  |

| **Eastern Europe** | |
| --- | --- |
|  |  |
|  |  |
| **Africa** | |
|  |  |

1. Hypopharynx - young

| **Asia** | |
| --- | --- |
|  |  |
|  |  |
|  |  |

|  |  |
| --- | --- |
|  |  |
| **Oceania** | |
|  |  |

| **Northern America** | |
| --- | --- |
|  |  |
| **Southern America** | |
|  |  |
|  |  |

| **Northern Europe** | |
| --- | --- |
|  |  |
|  |  |
|  |  |
|  |  |

| **Western Europe** | |
| --- | --- |
|  |  |
|  |  |
|  |  |

| **Southern Europe** | |
| --- | --- |
|  |  |
|  |  |
|  |  |

| **Eastern Europe** | |
| --- | --- |
|  |  |
|  |  |
| **Africa** | |
|  |  |

1. Hypopharynx - old

| **Asia** | |
| --- | --- |
|  |  |
|  |  |
|  |  |

|  |  |
| --- | --- |
|  |  |
| **Oceania** | |
|  |  |

| **Northern America** | |
| --- | --- |
|  |  |
| **Southern America** | |
|  |  |
|  |  |

| **Northern Europe** | |
| --- | --- |
|  |  |
|  |  |
|  |  |
|  |  |

| **Western Europe** | |
| --- | --- |
|  |  |
|  |  |
|  |  |

| **Southern Europe** | |
| --- | --- |
|  |  |
|  |  |
|  |  |

| **Eastern Europe** | |
| --- | --- |
|  |  |
|  |  |
| **Africa** | |
|  |  |

**Table S1.** Risk factors associations for pharyngeal cancer incidence by anatomical sites

1. **pharynx**

| **Overall** | | | | | |
| --- | --- | --- | --- | --- | --- |
| **Outcome** | **Risk factor** |  |  |  |  |
|  |  | ***β*** | ***95% CI*** |  | ***P*** |
|  | HDI | 0.270 | 0.084 | 0.457 | 0.005* |
|  | GDP per capita | 0.110 | -0.035 | 0.254 | 0.135 |
|  | Smoking | 0.115 | 0.066 | 0.164 | <0.001* |
|  | Alcohol drinking | 0.100 | 0.047 | 0.154 | <0.001* |
| **All Sexes and ages** | Dietary | 0.054 | 0.029 | 0.079 | <0.001* |
|  | Physical inactivity | -0.038 | -0.130 | 0.055 | 0.423 |
|  | Obesity | -0.019 | -0.045 | 0.008 | 0.166 |
|  | Hypertension | 0.046 | 0.012 | 0.079 | 0.008* |
|  | Diabetes | 0.002 | -0.053 | 0.056 | 0.946 |
|  | Lipid | 0.044 | 0.021 | 0.068 | <0.001* |
|  |  |  |  |  |  |
|  | HDI | 0.515 | 0.200 | 0.830 | 0.002* |
|  | GDP per capita | 0.203 | -0.043 | 0.449 | 0.105 |
|  | Smoking | 0.164 | 0.105 | 0.222 | <0.001* |
|  | Alcohol drinking | 0.174 | 0.111 | 0.237 | <0.001* |
| **Male** | Dietary | 0.076 | 0.043 | 0.109 | <0.001* |
|  | Physical inactivity | -0.128 | -0.285 | 0.028 | 0.108 |
|  | Obesity | -0.004 | -0.049 | 0.041 | 0.859 |
|  | Hypertension | 0.108 | 0.056 | 0.160 | <0.001* |
|  | Diabetes | 0.025 | -0.063 | 0.113 | 0.575 |
|  | Lipid | 0.082 | 0.043 | 0.121 | <0.001* |
|  |  |  |  |  |  |
|  | HDI | 0.048 | -0.044 | 0.141 | 0.304 |
|  | GDP per capita | 0.021 | -0.051 | 0.092 | 0.570 |
|  | Smoking | 0.005 | -0.022 | 0.032 | 0.708 |
|  | Alcohol drinking | -0.005 | -0.042 | 0.033 | 0.808 |
| **Female** | Dietary | 0.022 | 0.008 | 0.037 | 0.003* |

|  | Physical inactivity | -0.012 | -0.053 | 0.030 | 0.576 |
| --- | --- | --- | --- | --- | --- |
|  | Obesity | -0.022 | -0.033 | -0.010 | <0.001* |
|  | Hypertension | -0.010 | -0.025 | 0.006 | 0.231 |
|  | Diabetes | -0.016 | -0.043 | 0.011 | 0.230 |
|  | Lipid | 0.004 | -0.008 | 0.017 | 0.477 |
|  |  |  |  |  |  |
|  | HDI | 0.017 | -0.102 | 0.135 | 0.780 |
|  | GDP per capita | -0.040 | -0.137 | 0.056 | 0.411 |
|  | Smoking | 0.030 | -0.005 | 0.065 | 0.094 |
|  | Alcohol drinking | -0.009 | -0.042 | 0.024 | 0.573 |
| **Young** | Dietary | 0.018 | 0.002 | 0.034 | 0.027* |
|  | Physical inactivity | -0.039 | -0.098 | 0.019 | 0.187 |
|  | Obesity | -0.021 | -0.037 | -0.005 | 0.011* |
|  | Hypertension | -0.006 | -0.036 | 0.025 | 0.717 |
|  | Diabetes | -0.027 | -0.115 | 0.062 | 0.549 |
|  | Lipid | 0.009 | -0.008 | 0.026 | 0.301 |
|  |  |  |  |  |  |
|  | HDI | 1.408 | 0.691 | 2.126 | <0.001* |
|  | GDP per capita | 0.744 | 0.188 | 1.300 | 0.009* |
|  | Smoking | 0.449 | 0.287 | 0.610 | <0.001* |
|  | Alcohol drinking | 0.527 | 0.315 | 0.739 | <0.001* |
| **Old** | Dietary | 0.215 | 0.131 | 0.299 | <0.001* |
|  | Physical inactivity | -0.163 | -0.508 | 0.183 | 0.355 |
|  | Obesity | -0.048 | -0.130 | 0.035 | 0.256 |
|  | Hypertension | 0.081 | -0.026 | 0.188 | 0.138 |
|  | Diabetes | -0.118 | -0.234 | -0.002 | 0.045* |
|  | Lipid | 0.202 | 0.110 | 0.294 | <0.001* |

The analysis was conducted using univariable linear regression model at a country level.

*β*, beta coefficient. The beta coefficient can be interpreted as the change in incidence or mortality associated with one percent increase of a certain risk factor.

CI, confidence interval; ASR, age-standardized rate; HDI, human development index; GDP, gross domestic products.

* *p* values less than 0.05.

1. **Nasopharynx**

| **Overall** | | | | | |
| --- | --- | --- | --- | --- | --- |
| **Outcome** | **Risk factor** |  |  |  |  |
|  |  | ***β*** | ***95% CI*** |  | ***P*** |
|  | HDI | -0.023 | -0.167 | 0.121 | 0.757 |
|  | GDP per capita | -0.077 | -0.188 | 0.034 | 0.172 |
|  | Smoking | -0.006 | -0.045 | 0.033 | 0.746 |
|  | Alcohol drinking | -0.074 | -0.114 | -0.034 | <0.001* |
| **All Sexes and ages** | Dietary | 0.017 | -0.003 | 0.036 | 0.088 |
|  | Physical inactivity | -0.026 | -0.096 | 0.043 | 0.455 |
|  | Obesity | -0.030 | -0.049 | -0.011 | 0.003* |
|  | Hypertension | -0.027 | -0.052 | -0.002 | 0.036* |
|  | Diabetes | -0.018 | -0.060 | 0.023 | 0.389 |
|  | Lipid | -0.008 | -0.027 | 0.010 | 0.387 |
|  |  |  |  |  |  |
|  | HDI | -0.021 | -0.229 | 0.188 | 0.843 |
|  | GDP per capita | -0.106 | -0.267 | 0.056 | 0.198 |
|  | Smoking | 0.028 | -0.013 | 0.068 | 0.182 |
|  | Alcohol drinking | -0.065 | -0.107 | -0.023 | 0.003* |
| **Male** | Dietary | 0.009 | -0.013 | 0.031 | 0.410 |
|  | Physical inactivity | -0.024 | -0.124 | 0.076 | 0.640 |
|  | Obesity | -0.035 | -0.063 | -0.007 | 0.014* |
|  | Hypertension | -0.032 | -0.066 | 0.003 | 0.071 |
|  | Diabetes | -0.019 | -0.076 | 0.038 | 0.515 |
|  | Lipid | -0.012 | -0.038 | 0.015 | 0.387 |
|  |  |  |  |  |  |
|  | HDI | -0.032 | -0.117 | 0.054 | 0.466 |
|  | GDP per capita | -0.061 | -0.132 | 0.010 | 0.090 |
|  | Smoking | -0.044 | -0.067 | -0.020 | <0.001* |
|  | Alcohol drinking | -0.071 | -0.104 | -0.038 | <0.001* |

| **Female** | Dietary | 0.019 | 0.005 | 0.032 | 0.007* |
| --- | --- | --- | --- | --- | --- |
|  | Physical inactivity | -0.025 | -0.063 | 0.013 | 0.201 |
|  | Obesity | -0.021 | -0.032 | -0.010 | <0.001* |
|  | Hypertension | -0.021 | -0.036 | -0.007 | 0.004* |
|  | Diabetes | -0.013 | -0.040 | 0.014 | 0.332 |
|  | Lipid | -0.008 | -0.019 | 0.003 | 0.143 |
|  |  |  |  |  |  |
|  | HDI | -0.048 | -0.158 | 0.061 | 0.388 |
|  | GDP per capita | -0.091 | -0.181 | -0.001 | 0.046* |
|  | Smoking | -0.013 | -0.046 | 0.021 | 0.456 |
|  | Alcohol drinking | -0.059 | -0.088 | -0.030 | <0.001* |
| **Young** | Dietary | 0.002 | -0.013 | 0.017 | 0.776 |
|  | Physical inactivity | -0.021 | -0.074 | 0.033 | 0.449 |
|  | Obesity | -0.020 | -0.035 | -0.006 | 0.007* |
|  | Hypertension | -0.025 | -0.054 | 0.003 | 0.079 |
|  | Diabetes | -0.004 | -0.087 | 0.078 | 0.915 |
|  | Lipid | -0.006 | -0.022 | 0.010 | 0.462 |
|  |  |  |  |  |  |
|  | HDI | -0.083 | -0.573 | 0.408 | 0.739 |
|  | GDP per capita | -0.212 | -0.584 | 0.160 | 0.262 |
|  | Smoking | -0.023 | -0.138 | 0.092 | 0.688 |
|  | Alcohol drinking | -0.245 | -0.384 | -0.106 | 0.001* |
| **Old** | Dietary | 0.108 | 0.054 | 0.163 | <0.001* |
|  | Physical inactivity | -0.049 | -0.272 | 0.174 | 0.665 |
|  | Obesity | -0.088 | -0.140 | -0.037 | 0.001* |
|  | Hypertension | -0.023 | -0.092 | 0.047 | 0.524 |
|  | Diabetes | 0.011 | -0.065 | 0.087 | 0.773 |
|  | Lipid | -0.032 | -0.096 | 0.033 | 0.332 |

The analysis was conducted using univariable linear regression model at a country level.

*β*, beta coefficient. The beta coefficient can be interpreted as the change in incidence or mortality associated with one percent increase of a certain risk factor.CI, confidence interval; HDI, human development index.

GDP, gross domestic products. * *p* values less than 0.05.

**c) Oropharynx**

| **Overall** | | | | | |
| --- | --- | --- | --- | --- | --- |
| **Outcome** | **Risk factor** |  |  |  |  |
|  |  | ***β*** | ***95% CI*** | | ***P*** |
|  | HDI | 0.226 | 0.133 | 0.318 | <0.001* |
|  | GDP per capita | 0.174 | 0.106 | 0.242 | <0.001* |
|  | Smoking | 0.074 | 0.050 | 0.098 | <0.001* |
|  | Alcohol drinking | 0.118 | 0.095 | 0.140 | <0.001* |
| **All Sexes and ages** | Dietary | 0.019 | 0.006 | 0.032 | 0.004* |
|  | Physical inactivity | 0.007 | -0.041 | 0.054 | 0.783 |
|  | Obesity | 0.011 | -0.002 | 0.025 | 0.098 |
|  | Hypertension | 0.045 | 0.029 | 0.061 | <0.001* |
|  | Diabetes | 0.023 | -0.005 | 0.051 | 0.112 |
|  | Lipid | 0.037 | 0.025 | 0.048 | <0.001* |
|  |  |  |  |  |  |
|  | HDI | 0.384 | 0.219 | 0.548 | <0.001* |
|  | GDP per capita | 0.282 | 0.159 | 0.404 | <0.001* |
|  | Smoking | 0.066 | 0.034 | 0.098 | <0.001* |
|  | Alcohol drinking | 0.146 | 0.117 | 0.175 | <0.001* |
| **Male** | Dietary | 0.034 | 0.016 | 0.052 | <0.001* |
|  | Physical inactivity | -0.053 | -0.137 | 0.030 | 0.205 |
|  | Obesity | 0.025 | 0.001 | 0.049 | 0.039* |
|  | Hypertension | 0.080 | 0.053 | 0.106 | <0.001* |
|  | Diabetes | 0.048 | 0.001 | 0.096 | 0.045* |
|  | Lipid | 0.060 | 0.040 | 0.080 | <0.001* |
|  |  |  |  |  |  |
|  | HDI | 0.093 | 0.051 | 0.135 | <0.001* |
|  | GDP per capita | 0.081 | 0.050 | 0.111 | <0.001* |
|  | Smoking | 0.041 | 0.031 | 0.052 | <0.001* |
|  | Alcohol drinking | 0.060 | 0.045 | 0.074 | <0.001* |
| **Female** | Dietary | 0.001 | -0.006 | 0.008 | 0.725 |
|  | Physical inactivity | 0.024 | 0.004 | 0.044 | 0.021* |
|  | Obesity | 0.003 | -0.003 | 0.009 | 0.295 |
|  | Hypertension | 0.010 | 0.003 | 0.018 | 0.006* |

|  | Diabetes | 0.008 | -0.006 | 0.021 | 0.263 |
| --- | --- | --- | --- | --- | --- |
|  | Lipid | 0.013 | 0.008 | 0.019 | <0.001* |
|  |  |  |  |  |  |
|  | HDI | 0.068 | 0.024 | 0.112 | 0.003* |
|  | GDP per capita | 0.058 | 0.023 | 0.094 | 0.002* |
|  | Smoking | 0.030 | 0.017 | 0.042 | <0.001* |
|  | Alcohol drinking | 0.038 | 0.026 | 0.049 | <0.001* |
| **Young** | Dietary | 0.009 | 0.003 | 0.015 | 0.005* |
|  | Physical inactivity | -0.001 | -0.027 | 0.024 | 0.932 |
|  | Obesity | 0.008 | 0.001 | 0.015 | 0.030* |
|  | Hypertension | 0.013 | 0.001 | 0.025 | 0.035* |
|  | Diabetes | 0.009 | -0.030 | 0.047 | 0.662 |
|  | Lipid | 0.014 | 0.008 | 0.020 | <0.001* |
|  |  |  |  |  |  |
|  | HDI | 1.046 | 0.650 | 1.443 | <0.001* |
|  | GDP per capita | 0.823 | 0.535 | 1.110 | <0.001* |
|  | Smoking | 0.270 | 0.181 | 0.359 | <0.001* |
|  | Alcohol drinking | 0.517 | 0.418 | 0.617 | <0.001* |
| **Old** | Dietary | 0.055 | 0.004 | 0.105 | 0.036* |
|  | Physical inactivity | -0.027 | -0.221 | 0.166 | 0.781 |
|  | Obesity | 0.029 | -0.018 | 0.076 | 0.222 |
|  | Hypertension | 0.056 | -0.004 | 0.115 | 0.069 |
|  | Diabetes | -0.067 | -0.134 | -0.001 | 0.048* |
|  | Lipid | 0.148 | 0.098 | 0.197 | <0.001* |

The analysis was conducted using univariable linear regression model at a country level.

β, beta coefficient. The beta coefficient can be interpreted as the change in incidence or mortality associated with one percent increase of a certain risk factor.

CI, confidence interval; HDI, human development index; GDP, gross domestic products.

* p values less than 0.05.

**d). Hypopharynx**

| **Overall** | | | | | |
| --- | --- | --- | --- | --- | --- |
| **Outcome** | **Risk factor** |  |  |  |  |
|  |  | ***β*** | ***95% CI*** | | ***P*** |
|  | HDI | 0.056 | -0.022 | 0.134 | 0.158 |
|  | GDP per capita | 0.013 | -0.047 | 0.073 | 0.672 |
|  | Smoking | 0.042 | 0.022 | 0.062 | <0.001* |
|  | Alcohol drinking | 0.051 | 0.031 | 0.072 | <0.001* |
| **All Sexes and ages** | Dietary | 0.018 | 0.008 | 0.028 | <0.001* |
|  | Physical inactivity | -0.024 | -0.060 | 0.013 | 0.208 |
|  | Obesity | -0.002 | -0.013 | 0.009 | 0.717 |
|  | Hypertension | 0.026 | 0.013 | 0.039 | <0.001* |
|  | Diabetes | 0.006 | -0.018 | 0.030 | 0.616 |
|  | Lipid | 0.015 | 0.005 | 0.025 | 0.003* |
|  |  |  |  |  |  |
|  | HDI | 0.132 | -0.016 | 0.281 | 0.081 |
|  | GDP per capita | 0.024 | -0.091 | 0.139 | 0.678 |
|  | Smoking | 0.064 | 0.037 | 0.091 | <0.001* |
|  | Alcohol drinking | 0.089 | 0.062 | 0.116 | <0.001* |
| **Male** | Dietary | 0.031 | 0.016 | 0.046 | <0.001* |
|  | Physical inactivity | -0.069 | -0.138 | 0.001 | 0.053 |
|  | Obesity | 0.003 | -0.018 | 0.024 | 0.793 |
|  | Hypertension | 0.059 | 0.036 | 0.082 | <0.001* |
|  | Diabetes | 0.010 | -0.033 | 0.053 | 0.640 |
|  | Lipid | 0.032 | 0.014 | 0.050 | 0.001* |
|  |  |  |  |  |  |
|  | HDI | -0.012 | -0.040 | 0.016 | 0.401 |
|  | GDP per capita | -0.007 | -0.028 | 0.014 | 0.500 |
|  | Smoking | 0.001 | -0.007 | 0.008 | 0.895 |
|  | Alcohol drinking | -0.005 | -0.015 | 0.006 | 0.413 |
| **Female** | Dietary | 0.001 | -0.003 | 0.006 | 0.512 |
|  | Physical inactivity | -0.002 | -0.015 | 0.010 | 0.703 |
|  | Obesity | -0.002 | -0.006 | 0.002 | 0.368 |

|  | Hypertension | -0.001 | -0.006 | 0.004 | 0.656 |
| --- | --- | --- | --- | --- | --- |
|  | Diabetes | -0.001 | -0.010 | 0.008 | 0.887 |
|  | Lipid | -0.001 | -0.005 | 0.003 | 0.629 |
|  |  |  |  |  |  |
|  | HDI | -0.007 | -0.044 | 0.029 | 0.692 |
|  | GDP per capita | -0.015 | -0.049 | 0.018 | 0.371 |
|  | Smoking | 0.012 | 0.0002 | 0.023 | 0.046* |
|  | Alcohol drinking | 0.008 | -0.002 | 0.018 | 0.099 |
| **Young** | Dietary | 0.007 | 0.001 | 0.012 | 0.012* |
|  | Physical inactivity | -0.015 | -0.034 | 0.005 | 0.134 |
|  | Obesity | -0.003 | -0.009 | 0.002 | 0.220 |
|  | Hypertension | 0.008 | -0.002 | 0.018 | 0.117 |
|  | Diabetes | 0.003 | -0.030 | 0.036 | 0.844 |
|  | Lipid | 0.002 | -0.003 | 0.008 | 0.390 |
|  |  |  |  |  |  |
|  | HDI | 0.335 | -0.011 | 0.682 | 0.058 |
|  | GDP per capita | 0.123 | -0.140 | 0.386 | 0.357 |
|  | Smoking | 0.170 | 0.096 | 0.244 | <0.001* |
|  | Alcohol drinking | 0.239 | 0.144 | 0.335 | <0.001* |
| **Old** | Dietary | 0.070 | 0.030 | 0.110 | 0.001* |
|  | Physical inactivity | -0.118 | -0.273 | 0.038 | 0.136 |
|  | Obesity | 0.005 | -0.034 | 0.043 | 0.810 |
|  | Hypertension | 0.060 | 0.011 | 0.110 | 0.017* |
|  | Diabetes | -0.071 | -0.130 | -0.012 | 0.018* |
|  | Lipid | 0.065 | 0.021 | 0.109 | 0.004* |

The analysis was conducted using univariable linear regression model at a country level.

*β*, beta coefficient. The beta coefficient can be interpreted as the change in incidence or mortality associatedwith one percent increase of a certain risk factor.

CI, confidence interval; HDI, human development index.GDP, gross domestic products.

* *p* values less than 0.05.

**Table S2.** Joinpoint regression for pharyngeal cancer incidence by anatomical sites

1. pharynx– both

| **Region** | **AAPC** | | **Lower CI** | | **Upper CI** | | | **p-value** | **Significant** | |  |
| --- | --- | --- | --- | --- | --- | --- | --- | --- | --- | --- | --- |
| ***Asia*** |  | |  | |  | | |  |  | |  |
| Bahrain | -16.87 | | -31.65 | | 1.12 | | | 0.061 |  | |  |
| China | 4.74 | | 0.23 | | 9.47 | | | 0.042 | * | |  |
| India | -2.17 | | -6.72 | | 2.61 | | | 0.319 |  | |  |
| Israel | 3.10 | | -3.18 | | 9.79 | | | 0.295 |  | |  |
| Japan | 7.04 | | 3.18 | | 11.04 | | | 0.003 | * | |  |
| Korea | 4.50 | | 2.64 | | 6.38 | | | <0.001 | * | |  |
| Kuwait | -3.29 | | -8.78 | | 2.54 | | | 0.224 |  | |  |
| Philippines | -1.40 | | -8.13 | | 5.82 | | | 0.657 |  | |  |
| Thailand | 0.69 | | -3.33 | | 4.88 | | | 0.706 |  | |  |
| Turkey | 1.76 | | -8.59 | | 13.28 | | | 0.718 |  | |  |
| ***Oceania*** |  | |  | |  | | |  |  | |  |
| Australia | 5.41 | | 4.01 | | 6.84 | | | <0.001 | * | |  |
| New Zealand | 6.12 | | 1.68 | | 10.74 | | | 0.012 | * | |  |
| ***Northern America*** |  | |  | |  | | |  |  | |  |
| Canada | 3.90 | | 2.22 | | 5.60 | | | 0.001 | * | |  |
| USA | 3.13 | | 1.35 | | 4.95 | | | 0.001 | * | |  |
| ***Southern America*** |  | |  | |  | | |  |  | |  |
| Brazil | 14.47 | | -1.71 | | 33.31 | | | 0.082 |  | |  |
| Chile | -0.33 | | -10.58 | | 11.10 | | | 0.945 |  | |  |
| Colombia | 4.50 | | -3.83 | | 13.55 | | | 0.256 |  | |  |
| Ecuador | -1.56 | | -11.90 | | 10.01 | | | 0.753 |  | |  |
| Martinique | 0.90 | | -7.40 | | 9.94 | | | 0.816 |  | |  |
| ***Northern Europe*** |  | |  | |  | | |  |  | |  |
| Denmark | 4.80 | | 3.36 | | 6.26 | | | <0.001 | * | |  |
| Estonia | 7.17 | | 2.24 | | 12.34 | | | 0.010 | * | |  |
| Iceland | | | 11.74 | | -0.14 | | 25.05 | 0.052 | | |  |
| Ireland | | | 6.09 | | -0.37 | | 12.97 | 0.062 | | |  |
| Lithuania | | | -1.39 | | -6.10 | | 3.56 | 0.529 | | |  |
| Norway | | | 6.23 | | 3.26 | | 9.29 | 0.001 | | | * |
| United Kingdom | | | 7.60 | | 6.46 | | 8.75 | <0.001 | | | * |
| ***Western Europe*** | | |  | |  | |  |  | | |  |
| Austria | | | 0.92 | | -1.42 | | 3.31 | 0.394 | | |  |
| France | | | 1.11 | | -0.62 | | 2.87 | 0.179 | | |  |
| Germany | | | -0.77 | | -4.88 | | 3.52 | 0.685 | | |  |
| Netherlands | | | 2.75 | | 0.67 | | 4.87 | 0.016 | | | * |
| Switzerland | | | -0.20 | | -4.23 | | 4.01 | 0.915 | | |  |
| ***Southern Europe*** | | |  | |  | |  |  | | |  |
| Croatia | | | -1.07 | | -3.61 | | 1.54 | 0.369 | | |  |
| Cyprus | | | 24.42 | | 10.88 | | 39.61 | 0.002 | | | * |
| Italy | | | 0.91 | | -3.65 | | 5.69 | 0.663 | | |  |
| Malta | | | -3.74 | | -15.13 | | 9.19 | 0.506 | | |  |
| Slovenia | | | 3.76 | | -0.55 | | 8.26 | 0.080 | | |  |
| Spain | | | 0.07 | | -3.34 | | 3.61 | 0.962 | | |  |
| ***Eastern Europe*** | | |  | |  | |  |  | | |  |
| Bulgaria | | | 1.97 | | -4.17 | | 8.50 | 0.538 | | |  |
| Czech Republic | | | 3.22 | | 1.37 | | 5.11 | 0.004 | | | * |
| Poland | | | 11.39 | | -0.13 | | 24.24 | 0.052 | | |  |
| ***Africa*** | | |  | |  | |  |  | | |  |
| Uganda | | | -4.47 | | -17.55 | | 10.67 | 0.494 | | |  |

AAPC, annual percentage change; CI, confidence interval; * p values less than 0.05.

1. pharynx- male

| **Region** | **AAPC** | **Lower CI** | **Upper CI** | **p-value** | **Significant** |
| --- | --- | --- | --- | --- | --- |
| ***Asia*** |  |  |  |  |  |
| Bahrain | -11.26 | -21.15 | -0.13 | 0.047 | * |
| China | 3.04 | -3.70 | 10.24 | 0.337 |  |
| India | -5.68 | -9.97 | -1.19 | 0.020 | * |
| Israel | 0.87 | -4.86 | 6.96 | 0.741 |  |
| Japan | 10.07 | 2.63 | 18.05 | 0.013 | * |
| Korea | 5.03 | 2.27 | 7.86 | 0.003 | * |
| Kuwait | -4.91 | -11.01 | 1.61 | 0.118 |  |
| Philippines | -2.83 | -8.69 | 3.41 | 0.318 |  |
| Thailand | -0.04 | -5.37 | 5.60 | 0.988 |  |
| Turkey | 2.47 | -6.78 | 12.64 | 0.568 |  |
| ***Oceania*** |  |  |  |  |  |
| Australia | 4.99 | 3.84 | 6.16 | <0.001 | * |
| New Zealand | 5.15 | 1.07 | 9.39 | 0.019 | * |
| ***Northern America*** |  |  |  |  |  |
| Canada | 2.76 | 1.20 | 4.34 | 0.003 | * |
| USA | 2.34 | 0.47 | 4.25 | 0.014 | * |
| ***Southern America*** |  |  |  |  |  |
| Brazil | -3.24 | -10.53 | 4.63 | 0.360 |  |
| Chile | 3.08 | -1.83 | 8.23 | 0.190 |  |
| Colombia | 6.88 | -1.64 | 16.14 | 0.102 |  |
| Ecuador | 2.48 | -6.92 | 12.84 | 0.573 |  |
| Martinique | 0.13 | -9.99 | 11.38 | 0.979 |  |
| ***Northern Europe*** |  |  |  |  |  |
| Denmark | 1.99 | -0.05 | 4.07 | 0.055 |  |

| Estonia | 14.97 | 2.83 | 28.54 | 0.020 | * |
| --- | --- | --- | --- | --- | --- |
| Iceland | 9.32 | -4.64 | 25.33 | 0.171 |  |
| Ireland | 7.51 | 1.39 | 14.00 | 0.022 | * |
| Lithuania | 2.42 | -4.33 | 9.66 | 0.442 |  |
| Norway | 5.11 | 2.77 | 7.50 | 0.001 | * |
| United Kingdom | 6.84 | 5.45 | 8.25 | <0.001 | * |
| ***Western Europe*** |  |  |  |  |  |
| Austria | 0.32 | -2.23 | 2.94 | 0.780 |  |
| France | 0.79 | -0.82 | 2.44 | 0.292 |  |
| Germany | -3.21 | -7.77 | 1.58 | 0.158 |  |
| Netherlands | 2.72 | 0.17 | 5.33 | 0.039 | * |
| Switzerland | 0.46 | -3.61 | 4.71 | 0.803 |  |
| ***Southern Europe*** |  |  |  |  |  |
| Croatia | -3.29 | -6.26 | -0.23 | 0.038 | * |
| Cyprus | 14.57 | 1.02 | 29.95 | 0.037 | * |
| Italy | -0.07 | -4.74 | 4.84 | 0.975 |  |
| Malta | -3.99 | -14.60 | 7.93 | 0.445 |  |
| Slovenia | 5.00 | -0.97 | 11.32 | 0.091 |  |
| Spain | -1.36 | -5.57 | 3.05 | 0.492 |  |
| ***Eastern Europe*** |  |  |  |  |  |
| Bulgaria | 1.85 | -1.73 | 5.56 | 0.272 |  |
| Czech Republic | 2.55 | 0.74 | 4.40 | 0.011 | * |
| Poland | 20.05 | 1.51 | 41.97 | 0.036 | * |
| ***Africa*** |  |  |  |  |  |
| Uganda | -1.65 | -19.32 | 19.89 | 0.851 |  |

AAPC, annual percentage change; CI, confidence interval; * p values less than 0.05.

1. pharynx– female

| **Region** | **AAPC** | **Lower CI** | **Upper CI** | **p-value** | **Significant** |
| --- | --- | --- | --- | --- | --- |
| ***Asia*** |  |  |  |  |  |
| Bahrain | -6.40 | -15.12 | 3.21 | 0.185 |  |
| China | 4.33 | -1.13 | 10.10 | 0.107 |  |
| India | -3.71 | -10.29 | 3.35 | 0.253 |  |
| Israel | 1.99 | -4.57 | 9.01 | 0.513 |  |
| Japan | 9.10 | 2.21 | 16.45 | 0.015 | * |
| Korea | 5.25 | 3.20 | 7.33 | <0.001 | * |
| Kuwait | -6.17 | -10.21 | -1.94 | 0.010 | * |
| Philippines | -2.10 | -8.13 | 4.33 | 0.464 |  |
| Thailand | 0.27 | -4.14 | 4.88 | 0.894 |  |
| Turkey | 3.65 | -7.48 | 16.12 | 0.487 |  |
| ***Oceania*** |  |  |  |  |  |
| Australia | 5.24 | 3.75 | 6.74 | <0.001 | * |
| New Zealand | 5.82 | 0.96 | 10.91 | 0.024 | * |
| ***Northern America*** |  |  |  |  |  |
| Canada | 3.74 | 1.88 | 5.63 | 0.002 | * |
| USA | 3.00 | 1.48 | 4.55 | <0.001 | * |
| ***Southern America*** |  |  |  |  |  |
| Brazil | -5.61 | -12.74 | 2.10 | 0.128 |  |
| Chile | 5.25 | 4.15 | 6.35 | <0.001 | * |
| Colombia | 3.94 | -4.48 | 13.11 | 0.322 |  |
| Ecuador | -1.06 | -11.12 | 10.14 | 0.824 |  |
| Martinique | 6.24 | -2.32 | 15.54 | 0.135 |  |
| ***Northern Europe*** |  |  |  |  |  |
| Denmark | 3.21 | 1.42 | 5.02 | 0.003 | * |
| Estonia | 23.21 | 8.53 | 39.88 | 0.001 | * |

| Iceland | 9.36 | -4.25 | 24.91 | 0.159 |  |
| --- | --- | --- | --- | --- | --- |
| Ireland | 6.07 | -0.95 | 13.59 | 0.083 |  |
| Lithuania | 1.45 | -5.35 | 8.74 | 0.645 |  |
| Norway | 6.81 | 3.55 | 10.17 | 0.001 | * |
| United Kingdom | 7.59 | 6.30 | 8.89 | <0.001 | * |
| ***Western Europe*** |  |  |  |  |  |
| Austria | 1.23 | -1.61 | 4.16 | 0.350 |  |
| France | 2.03 | 0.64 | 3.44 | 0.010 | * |
| Germany | -1.47 | -7.72 | 5.21 | 0.617 |  |
| Netherlands | 3.35 | 0.45 | 6.32 | 0.028 | * |
| Switzerland | -0.42 | -4.51 | 3.84 | 0.822 |  |
| ***Southern Europe*** |  |  |  |  |  |
| Croatia | -1.25 | -4.25 | 1.84 | 0.373 |  |
| Cyprus | 37.92 | 13.64 | 67.38 | 0.005 | * |
| Italy | 0.57 | -4.02 | 5.37 | 0.787 |  |
| Malta | -3.95 | -15.59 | 9.29 | 0.492 |  |
| Slovenia | 5.49 | -1.39 | 12.86 | 0.105 |  |
| Spain | 1.32 | -2.73 | 5.53 | 0.480 |  |
| ***Eastern Europe*** |  |  |  |  |  |
| Bulgaria | 1.44 | -1.95 | 4.95 | 0.360 |  |
| Czech Republic | 3.88 | 1.88 | 5.92 | 0.002 | * |
| Poland | 34.85 | 0.00 | 81.84 | 0.050 |  |
| ***Africa*** |  |  |  |  |  |
| Uganda | 2.85 | -21.07 | 34.00 | 0.813 |  |

AAPC, annual percentage change; CI, confidence interval; * p values less than 0.05.

1. pharynx– young

| **Region** | **AAPC** | **Lower CI** | **Upper CI** | **p-value** | **Significant** |
| --- | --- | --- | --- | --- | --- |
| ***Asia*** |  |  |  |  |  |
| Bahrain | -13.14 | -27.04 | 3.40 | 0.099 |  |
| China | 4.15 | -0.63 | 9.16 | 0.081 |  |
| India | -1.69 | -5.94 | 2.74 | 0.398 |  |
| Israel | 3.48 | -2.68 | 10.03 | 0.234 |  |
| Japan | 6.29 | 3.01 | 9.68 | 0.002 | * |
| Korea | 4.22 | 2.28 | 6.20 | 0.001 | * |
| Kuwait | -3.54 | -9.69 | 3.02 | 0.242 |  |
| Philippines | -1.84 | -8.73 | 5.56 | 0.571 |  |
| Thailand | 1.31 | -2.67 | 5.45 | 0.475 |  |
| Turkey | 1.54 | -8.56 | 12.75 | 0.745 |  |
| ***Oceania*** |  |  |  |  |  |
| Australia | 5.54 | 4.15 | 6.94 | <0.001 | * |
| New Zealand | 6.40 | 1.80 | 11.20 | 0.012 | * |
| ***Northern America*** |  |  |  |  |  |
| Canada | 3.98 | 2.29 | 5.71 | 0.001 | * |
| USA | 3.24 | 1.38 | 5.14 | 0.001 | * |
| ***Southern America*** |  |  |  |  |  |
| Brazil | 14.02 | -6.71 | 39.36 | 0.200 |  |
| Chile | -0.12 | -8.76 | 9.34 | 0.977 |  |
| Colombia | 4.04 | -4.18 | 12.97 | 0.299 |  |
| Ecuador | -2.21 | -13.60 | 10.69 | 0.689 |  |
| Martinique | 1.22 | -6.83 | 9.98 | 0.744 |  |
| ***Northern Europe*** |  |  |  |  |  |
| Denmark | 5.71 | 4.40 | 7.04 | <0.001 | * |
| Estonia | 6.40 | 1.82 | 11.18 | 0.012 | * |

| Iceland | 11.85 | -0.84 | 26.15 | 0.064 |  |
| --- | --- | --- | --- | --- | --- |
| Ireland | 5.73 | -0.50 | 12.36 | 0.067 |  |
| Lithuania | -1.67 | -6.08 | 2.95 | 0.422 |  |
| Norway | 6.40 | 3.27 | 9.62 | 0.001 | * |
| United Kingdom | 7.61 | 6.52 | 8.71 | <0.001 | * |
| ***Western Europe*** |  |  |  |  |  |
| Austria | 1.13 | -1.13 | 3.44 | 0.285 |  |
| France | 1.15 | -0.67 | 3.01 | 0.183 |  |
| Germany | 0.04 | -3.98 | 4.24 | 0.980 |  |
| Netherlands | 2.58 | 0.81 | 4.38 | 0.010 | * |
| Switzerland | -0.34 | -4.25 | 3.73 | 0.851 |  |
| ***Southern Europe*** |  |  |  |  |  |
| Croatia | -0.87 | -3.31 | 1.64 | 0.444 |  |
| Cyprus | 26.18 | 13.92 | 39.75 | 0.001 | * |
| Italy | 1.21 | -3.30 | 5.94 | 0.559 |  |
| Malta | -3.07 | -15.23 | 10.84 | 0.607 |  |
| Slovenia | 3.62 | -0.30 | 7.69 | 0.066 |  |
| Spain | 0.67 | -2.61 | 4.07 | 0.653 |  |
| ***Eastern Europe*** |  |  |  |  |  |
| Bulgaria | 2.19 | -4.08 | 8.87 | 0.502 |  |
| Czech Republic | 3.16 | 1.25 | 5.11 | 0.005 | * |
| Poland | 9.83 | -0.26 | 20.95 | 0.055 |  |
| ***Africa*** |  |  |  |  |  |
| Uganda | 10.64 | -17.45 | 48.28 | 0.449 |  |

AAPC, annual percentage change; CI, confidence interval; * p values less than 0.05.

1. pharynx– old

| **Region** | **AAPC** | **Lower CI** | **Upper CI** | **p-value** | **Significant** |
| --- | --- | --- | --- | --- | --- |
| ***Asia*** |  |  |  |  |  |
| Bahrain | -4.28 | -14.62 | 7.32 | 0.404 |  |
| China | 4.23 | -1.51 | 10.29 | 0.130 |  |
| India | -0.45 | -4.44 | 3.72 | 0.808 |  |
| Israel | 4.24 | -1.61 | 10.43 | 0.136 |  |
| Japan | 5.58 | 2.53 | 8.73 | 0.003 | * |
| Korea | 3.63 | 1.33 | 5.98 | 0.006 | * |
| Kuwait | -2.75 | -9.55 | 4.55 | 0.400 |  |
| Philippines | -1.89 | -8.97 | 5.73 | 0.572 |  |
| Thailand | 1.43 | -2.43 | 5.44 | 0.423 |  |
| Turkey | -1.39 | -17.74 | 18.23 | 0.880 |  |
| ***Oceania*** |  |  |  |  |  |
| Australia | 5.76 | 4.21 | 7.34 | <0.001 | * |
| New Zealand | 6.94 | 1.65 | 12.50 | 0.016 | * |
| ***Northern America*** |  |  |  |  |  |
| Canada | 4.36 | 2.57 | 6.18 | <0.001 | * |
| USA | 3.96 | 2.87 | 5.07 | <0.001 | * |
| ***Southern America*** |  |  |  |  |  |
| Brazil | 5.40 | -9.67 | 22.99 | 0.454 |  |
| Chile | 0.42 | -4.31 | 5.38 | 0.847 |  |
| Colombia | 2.58 | -5.53 | 11.38 | 0.496 |  |
| Ecuador | -4.39 | -14.45 | 6.85 | 0.379 |  |
| Martinique | 1.25 | -8.05 | 11.49 | 0.773 |  |
| ***Northern Europe*** |  |  |  |  |  |
| Denmark | 7.16 | 5.76 | 8.59 | <0.001 | * |
| Estonia | 4.81 | 0.91 | 8.86 | 0.021 | * |

| Iceland | 12.59 | -0.40 | 27.28 | 0.056 |  |
| --- | --- | --- | --- | --- | --- |
| Ireland | 5.09 | -1.17 | 11.75 | 0.099 |  |
| Lithuania | -2.60 | -6.62 | 1.60 | 0.188 |  |
| Norway | 6.76 | 3.35 | 10.28 | 0.002 | * |
| United Kingdom | 7.85 | 6.85 | 8.85 | <0.001 | * |
| ***Western Europe*** |  |  |  |  |  |
| Austria | 1.32 | -0.85 | 3.53 | 0.200 |  |
| France | 1.14 | -0.92 | 3.25 | 0.239 |  |
| Germany | 0.88 | -2.83 | 4.73 | 0.604 |  |
| Netherlands | 2.45 | 0.98 | 3.94 | 0.005 | * |
| Switzerland | -0.41 | -4.48 | 3.83 | 0.825 |  |
| ***Southern Europe*** |  |  |  |  |  |
| Croatia | -0.15 | -2.50 | 2.26 | 0.891 |  |
| Cyprus | 26.93 | 13.52 | 41.93 | 0.001 | * |
| Italy | 1.93 | -2.56 | 6.63 | 0.356 |  |
| Malta | -3.38 | -16.00 | 11.13 | 0.586 |  |
| Slovenia | 3.06 | -0.38 | 6.61 | 0.075 |  |
| Spain | 1.11 | -1.97 | 4.29 | 0.434 |  |
| ***Eastern Europe*** |  |  |  |  |  |
| Bulgaria | 2.36 | -4.34 | 9.53 | 0.500 |  |
| Czech Republic | 3.22 | 1.19 | 5.30 | 0.006 | * |
| Poland | 8.77 | -1.02 | 19.52 | 0.074 |  |
| ***Africa*** |  |  |  |  |  |
| Uganda | 20.32 | -5.18 | 52.68 | 0.128 |  |

AAPC, annual percentage change; CI, confidence interval; * p values less than 0.05.

1. oropharynx– both

| **Region** | **AAPC** | **Lower CI** | **Upper CI** | **p-value** | **Significant** |
| --- | --- | --- | --- | --- | --- |
| ***Asia*** |  |  |  |  |  |
| Bahrain | -5.75 | -15.30 | 4.87 | 0.237 |  |
| China | 3.81 | -0.70 | 8.53 | 0.088 |  |
| India | -0.94 | -3.83 | 2.05 | 0.486 |  |
| Israel | 3.14 | -0.91 | 7.35 | 0.113 |  |
| Japan | 5.42 | 2.05 | 8.90 | 0.006 | * |
| Korea | 2.74 | -0.77 | 6.38 | 0.128 |  |
| Kuwait | -1.30 | -9.57 | 7.72 | 0.739 |  |
| Philippines | -3.17 | -9.30 | 3.37 | 0.288 |  |
| Thailand | 0.62 | -3.53 | 4.94 | 0.744 |  |
| Turkey | 0.20 | -7.32 | 8.32 | 0.955 |  |
| ***Oceania*** |  |  |  |  |  |
| Australia | 5.36 | 4.30 | 6.44 | <0.001 | * |
| New Zealand | 6.15 | 1.95 | 10.52 | 0.009 | * |
| ***Northern America*** |  |  |  |  |  |
| Canada | 3.29 | 1.51 | 5.10 | 0.003 | * |
| USA | 2.85 | 1.54 | 4.18 | <0.001 | * |
| ***Southern America*** |  |  |  |  |  |
| Brazil | 5.68 | -8.75 | 22.39 | 0.411 |  |
| Chile | 1.48 | -1.15 | 4.18 | 0.233 |  |
| Colombia | 4.68 | -1.03 | 10.72 | 0.097 |  |
| Ecuador | 0.57 | -7.97 | 9.89 | 0.887 |  |
| Martinique | -2.35 | -10.89 | 6.99 | 0.564 |  |
| ***Northern Europe*** |  |  |  |  |  |
| Denmark | 6.88 | 5.39 | 8.38 | <0.001 | * |
| Estonia | 3.10 | -1.03 | 7.40 | 0.123 |  |

| Iceland | 11.23 | -1.95 | 26.19 | 0.088 |  |
| --- | --- | --- | --- | --- | --- |
| Ireland | 5.93 | 1.27 | 10.80 | 0.018 | * |
| Lithuania | -2.54 | -6.09 | 1.15 | 0.149 |  |
| Norway | 5.34 | 3.09 | 7.64 | 0.001 | * |
| United Kingdom | 7.12 | 6.11 | 8.15 | <0.001 | * |
| ***Western Europe*** |  |  |  |  |  |
| Austria | 0.86 | -0.91 | 2.66 | 0.297 |  |
| France | 0.41 | -1.85 | 2.73 | 0.687 |  |
| Germany | 0.25 | -2.41 | 2.98 | 0.836 |  |
| Netherlands | 1.86 | 0.65 | 3.08 | 0.007 | * |
| Switzerland | 0.34 | -3.51 | 4.35 | 0.845 |  |
| ***Southern Europe*** |  |  |  |  |  |
| Croatia | -1.14 | -3.48 | 1.26 | 0.302 |  |
| Cyprus | 12.14 | 5.13 | 19.62 | 0.003 | * |
| Italy | 1.58 | -2.41 | 5.73 | 0.394 |  |
| Malta | -3.14 | -15.09 | 10.50 | 0.592 |  |
| Slovenia | 2.51 | -0.12 | 5.21 | 0.059 |  |
| Spain | -0.96 | -4.05 | 2.23 | 0.502 |  |
| ***Eastern Europe*** |  |  |  |  |  |
| Bulgaria | 3.22 | -4.49 | 11.55 | 0.424 |  |
| Czech Republic | 2.06 | 0.33 | 3.81 | 0.025 | * |
| Poland | 8.44 | 0.35 | 17.20 | 0.043 | * |
| ***Africa*** |  |  |  |  |  |
| Uganda | 17.91 | -1.20 | 40.72 | 0.068 |  |

AAPC, annual percentage change; CI, confidence interval; * p values less than 0.05.

1. oropharynx– male

| **Region** | **AAPC** | **Lower CI** | **Upper CI** | **p-value** | **Significant** |
| --- | --- | --- | --- | --- | --- |
| ***Asia*** |  |  |  |  |  |
| Bahrain | -7.36 | -17.53 | 4.05 | 0.167 |  |
| China | 4.08 | 0.46 | 7.82 | 0.031 | * |
| India | -1.14 | -4.54 | 2.38 | 0.472 |  |
| Israel | 4.45 | -1.10 | 10.31 | 0.103 |  |
| Japan | 5.94 | 2.06 | 9.96 | 0.007 | * |
| Korea | 3.02 | 0.48 | 5.63 | 0.025 | * |
| Kuwait | 1.79 | -4.88 | 8.93 | 0.562 |  |
| Philippines | -0.92 | -10.02 | 9.09 | 0.829 |  |
| Thailand | 0.63 | -3.41 | 4.83 | 0.735 |  |
| Turkey | -3.30 | -16.17 | 11.55 | 0.646 |  |
| ***Oceania*** |  |  |  |  |  |
| Australia | 5.74 | 4.61 | 6.89 | <0.001 | * |
| New Zealand | 6.46 | 2.89 | 10.14 | 0.003 | * |
| ***Northern America*** |  |  |  |  |  |
| Canada | 3.71 | 2.13 | 5.32 | 0.001 | * |
| USA | 3.43 | 2.29 | 4.59 | <0.001 | * |
| ***Southern America*** |  |  |  |  |  |
| Brazil | 5.11 | -7.97 | 20.05 | 0.412 |  |
| Chile | 2.07 | -1.09 | 5.32 | 0.172 |  |
| Colombia | 7.15 | -2.18 | 17.36 | 0.119 |  |
| Ecuador | 0.51 | -10.06 | 12.33 | 0.919 |  |
| Martinique | -4.17 | -13.46 | 6.12 | 0.364 |  |
| ***Northern Europe*** |  |  |  |  |  |
| Denmark | 7.08 | 5.19 | 9.00 | <0.001 | * |
| Estonia | 1.98 | -2.83 | 7.03 | 0.376 |  |

| Iceland | 17.16 | 5.74 | 29.81 | 0.007 | * |
| --- | --- | --- | --- | --- | --- |
| Ireland | 6.81 | 1.64 | 12.25 | 0.016 | * |
| Lithuania | -3.07 | -6.55 | 0.54 | 0.085 |  |
| Norway | 5.13 | -0.18 | 10.73 | 0.059 |  |
| United Kingdom | 7.11 | 6.09 | 8.14 | <0.001 | * |
| ***Western Europe*** |  |  |  |  |  |
| Austria | 0.07 | -1.88 | 2.05 | 0.940 |  |
| France | -0.75 | -3.06 | 1.62 | 0.483 |  |
| Germany | -0.46 | -2.53 | 1.66 | 0.626 |  |
| Netherlands | 1.41 | -0.18 | 3.02 | 0.076 |  |
| Switzerland | 0.47 | -3.97 | 5.12 | 0.817 |  |
| ***Southern Europe*** |  |  |  |  |  |
| Croatia | -1.61 | -4.39 | 1.25 | 0.227 |  |
| Cyprus | 10.86 | -0.95 | 24.08 | 0.068 |  |
| Italy | 1.57 | -3.24 | 6.63 | 0.480 |  |
| Malta | -3.88 | -13.11 | 6.33 | 0.393 |  |
| Slovenia | 1.77 | -1.07 | 4.69 | 0.191 |  |
| Spain | -2.30 | -5.51 | 1.02 | 0.147 |  |
| ***Eastern Europe*** |  |  |  |  |  |
| Bulgaria | 1.99 | -3.13 | 7.39 | 0.403 |  |
| Czech Republic | 1.22 | -0.45 | 2.91 | 0.131 |  |
| Poland | 7.50 | -0.58 | 16.23 | 0.065 |  |
| ***Africa*** |  |  |  |  |  |
| Uganda | -9.75 | -18.23 | -0.40 | 0.043 | * |

AAPC, annual percentage change; CI, confidence interval; * p values less than 0.05.

1. oropharynx– female

| **Region** | **AAPC** | **Lower CI** | **Upper CI** | **p-value** | **Significant** |
| --- | --- | --- | --- | --- | --- |
| ***Asia*** |  |  |  |  |  |
| Bahrain | 29.84 | 25.74 | 34.06 | <0.001 | * |
| China | -1.28 | -15.55 | 15.41 | 0.854 |  |
| India | 1.76 | -4.24 | 8.14 | 0.527 |  |
| Israel | -0.12 | -11.12 | 12.24 | 0.981 |  |
| Japan | 3.23 | -1.38 | 8.05 | 0.147 |  |
| Korea | 2.53 | -4.14 | 9.66 | 0.417 |  |
| Kuwait | -9.92 | -25.02 | 8.22 | 0.265 |  |
| Philippines | -12.95 | -27.74 | 4.87 | 0.124 |  |
| Thailand | 0.47 | -6.46 | 7.90 | 0.885 |  |
| Turkey | 5.00 | -9.04 | 21.20 | 0.456 |  |
| ***Oceania*** |  |  |  |  |  |
| Australia | 3.81 | 1.41 | 6.27 | 0.006 | * |
| New Zealand | 8.61 | -8.70 | 29.21 | 0.304 |  |
| ***Northern America*** |  |  |  |  |  |
| Canada | 1.40 | -1.93 | 4.85 | 0.364 |  |
| USA | 1.80 | -0.73 | 4.41 | 0.141 |  |
| ***Southern America*** |  |  |  |  |  |
| Brazil | -2.99 | -14.29 | 9.80 | 0.587 |  |
| Chile | NA | NA | NA | NA | NA |
| Colombia | 3.87 | -9.31 | 18.98 | 0.537 |  |
| Ecuador | -0.75 | -7.87 | 6.93 | 0.843 |  |
| Martinique | 31.13 | 17.28 | 46.61 | 0.001 | * |
| ***Northern Europe*** |  |  |  |  |  |
| Denmark | 6.01 | 2.43 | 9.71 | 0.004 | * |
| Estonia | 31.04 | 11.66 | 53.78 | 0.001 | * |

| Iceland | 8.27 | -8.60 | 28.24 | 0.311 |  |
| --- | --- | --- | --- | --- | --- |
| Ireland | 3.83 | -3.22 | 11.39 | 0.253 |  |
| Lithuania | 2.58 | -4.47 | 10.15 | 0.433 |  |
| Norway | 10.08 | 3.02 | 17.63 | 0.010 | * |
| United Kingdom | 7.00 | 5.19 | 8.85 | <0.001 | * |
| ***Western Europe*** |  |  |  |  |  |
| Austria | 4.10 | -0.29 | 8.69 | 0.064 |  |
| France | 5.62 | 1.41 | 10.01 | 0.015 | * |
| Germany | 2.47 | -3.25 | 8.52 | 0.356 |  |
| Netherlands | 2.73 | 0.40 | 5.12 | 0.027 | * |
| Switzerland | -1.48 | -9.59 | 7.36 | 0.699 |  |
| ***Southern Europe*** |  |  |  |  |  |
| Croatia | 2.74 | -2.85 | 8.64 | 0.297 |  |
| Cyprus | 10.50 | -9.39 | 34.77 | 0.324 |  |
| Italy | 2.99 | -4.89 | 11.52 | 0.418 |  |
| Malta | 4.99 | 0.98 | 9.14 | 0.014 | * |
| Slovenia | 5.52 | 0.70 | 10.57 | 0.029 | * |
| Spain | 6.44 | 0.13 | 13.15 | 0.046 | * |
| ***Eastern Europe*** |  |  |  |  |  |
| Bulgaria | 8.90 | 1.70 | 16.61 | 0.021 | * |
| Czech Republic | 4.81 | 2.50 | 7.18 | 0.001 | * |
| Poland | 8.27 | -3.49 | 21.47 | 0.150 |  |
| ***Africa*** |  |  |  |  |  |
| Uganda | -4.08 | -26.28 | 24.81 | 0.725 |  |

AAPC, annual percentage change; CI, confidence interval; * p values less than 0.05.

NA, not available as it reported zero cases during the period and joinpoint regression could not be performed in such circumstances.

1. oropharynx– young

| **Region** | **AAPC** | **Lower CI** | **Upper CI** | **p-value** | **Significant** |
| --- | --- | --- | --- | --- | --- |
| ***Asia*** |  |  |  |  |  |
| Bahrain | -7.96 | -16.54 | 1.49 | 0.096 |  |
| China | 8.27 | -5.60 | 24.18 | 0.218 |  |
| India | -10.36 | -17.37 | -2.75 | 0.015 | * |
| Israel | 1.90 | -13.19 | 19.62 | 0.793 |  |
| Japan | 3.70 | -4.65 | 12.78 | 0.348 |  |
| Korea | 5.76 | -1.51 | 13.57 | 0.107 |  |
| Kuwait | -13.17 | -24.36 | -0.34 | 0.045 | * |
| Philippines | -4.46 | -20.63 | 15.00 | 0.586 |  |
| Thailand | 4.30 | -6.75 | 16.67 | 0.411 |  |
| Turkey | -6.50 | -13.29 | 0.82 | 0.074 |  |
| ***Oceania*** |  |  |  |  |  |
| Australia | 3.52 | 0.94 | 6.17 | 0.013 | * |
| New Zealand | 6.07 | -0.64 | 13.24 | 0.071 |  |
| ***Northern America*** |  |  |  |  |  |
| Canada | -0.16 | -3.39 | 3.18 | 0.913 |  |
| USA | -0.03 | -2.56 | 2.57 | 0.978 |  |
| ***Southern America*** |  |  |  |  |  |
| Brazil | 3.94 | -8.32 | 17.84 | 0.498 |  |
| Chile | NA | NA | NA | NA | NA |
| Colombia | 17.52 | 4.41 | 32.28 | 0.014 | * |
| Ecuador | 13.15 | 1.29 | 26.39 | 0.029 | * |
| Martinique | -0.81 | -10.69 | 10.16 | 0.862 |  |
| ***Northern Europe*** |  |  |  |  |  |
| Denmark | 1.22 | -0.75 | 3.22 | 0.192 |  |
| Estonia | 3.25 | -12.29 | 21.53 | 0.663 |  |

| Iceland | 3.41 | -7.84 | 16.03 | 0.568 |  |
| --- | --- | --- | --- | --- | --- |
| Ireland | 4.52 | -6.02 | 16.26 | 0.365 |  |
| Lithuania | -2.75 | -18.42 | 15.94 | 0.724 |  |
| Norway | -2.37 | -5.32 | 0.67 | 0.109 |  |
| United Kingdom | 5.31 | 3.25 | 7.42 | <0.001 | * |
| ***Western Europe*** |  |  |  |  |  |
| Austria | -2.45 | -5.34 | 0.53 | 0.094 |  |
| France | -4.14 | -8.28 | 0.18 | 0.058 |  |
| Germany | -14.79 | -25.44 | -2.63 | 0.019 | * |
| Netherlands | -5.13 | -7.87 | -2.31 | 0.003 | * |
| Switzerland | 4.50 | -7.25 | 17.73 | 0.420 |  |
| ***Southern Europe*** |  |  |  |  |  |
| Croatia | -5.29 | -9.51 | -0.87 | 0.025 | * |
| Cyprus | 13.16 | 3.83 | 23.34 | 0.011 | * |
| Italy | -3.42 | -13.49 | 7.82 | 0.487 |  |
| Malta | 1.49 | 0.17 | 2.84 | 0.032 | * |
| Slovenia | -0.35 | -4.44 | 3.92 | 0.853 |  |
| Spain | -9.47 | -14.53 | -4.10 | 0.004 | * |
| ***Eastern Europe*** |  |  |  |  |  |
| Bulgaria | 2.40 | -0.97 | 5.89 | 0.140 |  |
| Czech Republic | -3.27 | -7.35 | 0.99 | 0.113 |  |
| Poland | 4.01 | -9.00 | 18.88 | 0.517 |  |
| ***Africa*** |  |  |  |  |  |
| Uganda | -3.10 | -24.87 | 24.99 | 0.783 |  |

AAPC, annual percentage change; CI, confidence interval; * p values less than 0.05.

NA, not available as it reported zero cases during the period and joinpoint regression could not be performed in such circumstances.

1. oropharynx- old

| **Region** | **AAPC** | **Lower CI** | **Upper CI** | **p-value** | **Significant** |
| --- | --- | --- | --- | --- | --- |
| ***Asia*** |  |  |  |  |  |
| Bahrain | 1.06 | -7.45 | 10.36 | 0.814 |  |
| China | 5.13 | 0.14 | 10.36 | 0.045 | * |
| India | 0.22 | -2.91 | 3.46 | 0.875 |  |
| Israel | 4.60 | -0.33 | 9.77 | 0.064 |  |
| Japan | 5.90 | 2.64 | 9.25 | 0.003 | * |
| Korea | 3.15 | 0.44 | 5.93 | 0.027 | * |
| Kuwait | -3.15 | -9.55 | 3.71 | 0.312 |  |
| Philippines | -1.35 | -8.84 | 6.74 | 0.700 |  |
| Thailand | 0.56 | -2.65 | 3.88 | 0.701 |  |
| Turkey | -1.12 | -16.96 | 17.74 | 0.899 |  |
| ***Oceania*** |  |  |  |  |  |
| Australia | 6.01 | 4.58 | 7.45 | <0.001 | * |
| New Zealand | 6.72 | 1.68 | 12.00 | 0.015 | * |
| ***Northern America*** |  |  |  |  |  |
| Canada | 4.25 | 2.39 | 6.14 | 0.001 | * |
| USA | 4.05 | 3.02 | 5.10 | <0.001 | * |
| ***Southern America*** |  |  |  |  |  |
| Brazil | 5.60 | -10.42 | 24.49 | 0.467 |  |
| Chile | -2.26 | -3.67 | -0.82 | 0.007 | * |
| Colombia | 3.23 | -3.91 | 10.90 | 0.337 |  |
| Ecuador | -3.81 | -14.59 | 8.32 | 0.472 |  |
| Martinique | -2.47 | -13.23 | 9.61 | 0.634 |  |
| ***Northern Europe*** |  |  |  |  |  |
| Denmark | 8.31 | 6.74 | 9.91 | <0.001 | * |
| Estonia | 4.31 | 1.41 | 7.30 | 0.009 | * |

| Iceland | 9.79 | -5.04 | 26.95 | 0.176 |  |
| --- | --- | --- | --- | --- | --- |
| Ireland | 5.66 | -0.41 | 12.09 | 0.064 |  |
| Lithuania | -2.97 | -6.69 | 0.89 | 0.113 |  |
| Norway | 6.96 | 3.56 | 10.48 | 0.001 | * |
| United Kingdom | 7.92 | 6.94 | 8.92 | <0.001 | * |
| ***Western Europe*** |  |  |  |  |  |
| Austria | 1.60 | -0.44 | 3.68 | 0.109 |  |
| France | 1.60 | -0.58 | 3.82 | 0.130 |  |
| Germany | 2.14 | -1.85 | 6.28 | 0.256 |  |
| Netherlands | 2.90 | 1.51 | 4.31 | 0.001 | * |
| Switzerland | -0.39 | -4.09 | 3.46 | 0.819 |  |
| ***Southern Europe*** |  |  |  |  |  |
| Croatia | -0.09 | -2.52 | 2.40 | 0.935 |  |
| Cyprus | 16.45 | 8.55 | 24.92 | 0.001 | * |
| Italy | 1.96 | -2.43 | 6.55 | 0.339 |  |
| Malta | -2.60 | -16.21 | 13.22 | 0.697 |  |
| Slovenia | 3.57 | -0.45 | 7.76 | 0.075 |  |
| Spain | 1.49 | -2.04 | 5.14 | 0.363 |  |
| ***Eastern Europe*** |  |  |  |  |  |
| Bulgaria | 3.54 | -5.01 | 12.86 | 0.429 |  |
| Czech Republic | 3.31 | 1.17 | 5.49 | 0.007 | * |
| Poland | 9.62 | 0.47 | 19.61 | 0.041 | * |
| ***Africa*** |  |  |  |  |  |
| Uganda | -9.51 | -19.67 | 1.93 | 0.089 |  |

AAPC, annual percentage change; CI, confidence interval; * p values less than 0.05.

1. nasopharynx– both

| **Region** | **AAPC** | **Lower CI** | **Upper CI** | **p-value** | **Significant** |
| --- | --- | --- | --- | --- | --- |
| ***Asia*** |  |  |  |  |  |
| Bahrain | -14.33 | -28.86 | 3.17 | 0.091 |  |
| China | -1.26 | -2.80 | 0.30 | 0.100 |  |
| India | -1.87 | -6.90 | 3.44 | 0.433 |  |
| Israel | -4.99 | -9.83 | 0.11 | 0.055 |  |
| Japan | 1.01 | -1.95 | 4.06 | 0.458 |  |
| Korea | -1.56 | -4.02 | 0.97 | 0.192 |  |
| Kuwait | 2.42 | -3.59 | 8.80 | 0.389 |  |
| Philippines | -4.52 | -6.43 | -2.58 | 0.001 | * |
| Thailand | -1.44 | -4.19 | 1.39 | 0.272 |  |
| Turkey | -1.17 | -5.83 | 3.71 | 0.588 |  |
| ***Oceania*** |  |  |  |  |  |
| Australia | 0.03 | -1.92 | 2.01 | 0.974 |  |
| New Zealand | -2.48 | -6.17 | 1.36 | 0.172 |  |
| ***Northern America*** |  |  |  |  |  |
| Canada | -0.55 | -2.23 | 1.16 | 0.478 |  |
| USA | -1.28 | -3.48 | 0.97 | 0.224 |  |
| ***Southern America*** |  |  |  |  |  |
| Brazil | -1.45 | -17.98 | 18.42 | 0.859 |  |
| Chile | NA | NA | NA | NA | NA |
| Colombia | -1.98 | -11.96 | 9.14 | 0.679 |  |
| Ecuador | -0.35 | -12.58 | 13.59 | 0.952 |  |
| Martinique | -7.84 | -21.17 | 7.75 | 0.263 |  |
| ***Northern Europe*** |  |  |  |  |  |
| Denmark | 2.72 | -1.37 | 6.98 | 0.166 |  |
| Estonia | -9.58 | -21.85 | 4.61 | 0.150 |  |

| Iceland | -12.19 | -22.90 | 0.02 | 0.050 |
| --- | --- | --- | --- | --- |
| Ireland | 3.67 | -0.20 | 7.69 | 0.060 |
| Lithuania | 2.44 | -5.05 | 10.52 | 0.486 |
| Norway | 1.70 | -7.71 | 12.08 | 0.699 |
| United Kingdom | -1.35 | -3.00 | 0.32 | 0.099 |
| ***Western Europe*** |  |  |  |  |
| Austria | -2.43 | -5.52 | 0.76 | 0.116 |
| France | -0.20 | -7.80 | 8.02 | 0.954 |
| Germany | 2.03 | -5.57 | 10.23 | 0.567 |
| Netherlands | 1.74 | -2.46 | 6.13 | 0.373 |
| Switzerland | -2.47 | -9.42 | 5.02 | 0.458 |
| ***Southern Europe*** |  |  |  |  |
| Croatia | 0.42 | -5.90 | 7.16 | 0.886 |
| Cyprus | 0.29 | -16.31 | 20.18 | 0.971 |
| Italy | 0.31 | -2.57 | 3.27 | 0.814 |
| Malta | 0.46 | -7.20 | 8.75 | 0.898 |
| Slovenia | 6.22 | -2.84 | 16.13 | 0.157 |
| Spain | -1.24 | -4.47 | 2.09 | 0.411 |
| ***Eastern Europe*** |  |  |  |  |
| Bulgaria | 1.45 | -1.43 | 4.42 | 0.282 |
| Czech Republic | -0.50 | -4.03 | 3.15 | 0.756 |
| Poland | -0.60 | -6.72 | 5.93 | 0.834 |
| ***Africa*** |  |  |  |  |
| Uganda | 5.06 | -9.75 | 22.31 | 0.475 |

AAPC, annual percentage change; CI, confidence interval; * p values less than 0.05.

NA, not available as it reported zero cases during the period and joinpoint regression could not be performed in such circumstances.

1. nasopharynx– male

| **Region** | **AAPC** | **Lower CI** | **Upper CI** | **p-value** | **Significant** |
| --- | --- | --- | --- | --- | --- |
| ***Asia*** |  |  |  |  |  |
| Bahrain | -12.80 | -27.38 | 4.71 | 0.123 |  |
| China | -0.37 | -2.53 | 1.84 | 0.706 |  |
| India | -2.68 | -12.71 | 8.50 | 0.581 |  |
| Israel | -3.34 | -7.39 | 0.89 | 0.105 |  |
| Japan | 0.13 | -5.16 | 5.72 | 0.958 |  |
| Korea | -1.42 | -3.90 | 1.13 | 0.233 |  |
| Kuwait | 1.94 | -12.48 | 18.73 | 0.805 |  |
| Philippines | -5.34 | -8.25 | -2.33 | 0.004 | * |
| Thailand | -0.59 | -3.72 | 2.64 | 0.683 |  |
| Turkey | 0.77 | -4.31 | 6.11 | 0.742 |  |
| ***Oceania*** |  |  |  |  |  |
| Australia | 0.21 | -1.78 | 2.23 | 0.819 |  |
| New Zealand | -0.73 | -5.54 | 4.32 | 0.741 |  |
| ***Northern America*** |  |  |  |  |  |
| Canada | -0.15 | -2.36 | 2.11 | 0.881 |  |
| USA | -1.06 | -3.39 | 1.33 | 0.335 |  |
| ***Southern America*** |  |  |  |  |  |
| Brazil | -1.81 | -22.19 | 23.91 | 0.861 |  |
| Chile | NA | NA | NA | NA | NA |
| Colombia | 1.82 | -11.75 | 17.47 | 0.779 |  |
| Ecuador | -8.51 | -18.61 | 2.83 | 0.136 |  |
| Martinique | -6.61 | -19.38 | 8.18 | 0.315 |  |
| ***Northern Europe*** |  |  |  |  |  |
| Denmark | 3.90 | -1.72 | 9.84 | 0.151 |  |
| Estonia | -3.77 | -16.49 | 10.89 | 0.550 |  |

| Iceland | -13.53 | -22.68 | -3.29 | 0.011 | * |
| --- | --- | --- | --- | --- | --- |
| Ireland | 2.53 | -2.38 | 7.69 | 0.274 |  |
| Lithuania | 3.68 | -6.33 | 14.76 | 0.435 |  |
| Norway | 1.72 | -10.22 | 15.24 | 0.761 |  |
| United Kingdom | -1.67 | -4.12 | 0.84 | 0.163 |  |
| ***Western Europe*** |  |  |  |  |  |
| Austria | -0.96 | -4.65 | 2.87 | 0.574 |  |
| France | -1.39 | -7.24 | 4.83 | 0.612 |  |
| Germany | 3.31 | -2.58 | 9.56 | 0.237 |  |
| Netherlands | 0.40 | -2.90 | 3.82 | 0.788 |  |
| Switzerland | -4.15 | -10.34 | 2.47 | 0.181 |  |
| ***Southern Europe*** |  |  |  |  |  |
| Croatia | 2.56 | -4.44 | 10.08 | 0.433 |  |
| Cyprus | -0.97 | -18.51 | 20.35 | 0.911 |  |
| Italy | 0.90 | -3.86 | 5.89 | 0.680 |  |
| Malta | -2.36 | -12.62 | 9.10 | 0.633 |  |
| Slovenia | 7.73 | -3.02 | 19.68 | 0.141 |  |
| Spain | -4.09 | -8.76 | 0.82 | 0.101 |  |
| ***Eastern Europe*** |  |  |  |  |  |
| Bulgaria | 1.99 | -1.00 | 5.07 | 0.166 |  |
| Czech Republic | -0.43 | -4.72 | 4.05 | 0.827 |  |
| Poland | 1.15 | -6.11 | 8.97 | 0.732 |  |
| ***Africa*** |  |  |  |  |  |
| Uganda | 4.69 | -12.77 | 25.65 | 0.578 |  |

AAPC, annual percentage change; CI, confidence interval; * p values less than 0.05.

NA, not available as it reported zero cases during the period and joinpoint regression could not be performed in such circumstances.

1. nasopharynx– female

| **Region** | **AAPC** | **Lower CI** | **Upper CI** | **p-value** | **Significant** |
| --- | --- | --- | --- | --- | --- |
| ***Asia*** |  |  |  |  |  |
| Bahrain | -15.50 | -24.76 | -5.11 | 0.010 | * |
| China | -3.89 | -6.07 | -1.65 | 0.001 | * |
| India | -0.20 | -6.75 | 6.82 | 0.948 |  |
| Israel | -5.44 | -12.30 | 1.97 | 0.126 |  |
| Japan | 3.25 | -2.18 | 8.99 | 0.209 |  |
| Korea | -2.57 | -6.65 | 1.68 | 0.197 |  |
| Kuwait | -4.57 | -18.82 | 12.17 | 0.523 |  |
| Philippines | -3.88 | -9.05 | 1.59 | 0.161 |  |
| Thailand | -2.82 | -5.73 | 0.18 | 0.062 |  |
| Turkey | -6.54 | -12.99 | 0.39 | 0.061 |  |
| ***Oceania*** |  |  |  |  |  |
| Australia | -0.43 | -4.02 | 3.30 | 0.795 |  |
| New Zealand | -5.04 | -14.82 | 5.87 | 0.305 |  |
| ***Northern America*** |  |  |  |  |  |
| Canada | -1.36 | -3.43 | 0.75 | 0.173 |  |
| USA | -1.82 | -5.99 | 2.55 | 0.360 |  |
| ***Southern America*** |  |  |  |  |  |
| Brazil | -2.20 | -18.80 | 17.79 | 0.789 |  |
| Chile | NA | NA | NA | NA | NA |
| Colombia | -12.42 | -25.02 | 2.30 | 0.085 |  |
| Ecuador | 5.46 | -2.14 | 13.64 | 0.140 |  |
| Martinique | -4.11 | -16.76 | 10.45 | 0.560 |  |
| ***Northern Europe*** |  |  |  |  |  |
| Denmark | -1.46 | -9.34 | 7.09 | 0.694 |  |
| Estonia | -8.70 | -23.30 | 8.67 | 0.263 |  |

| Iceland | -20.55 | -24.97 | -15.87 | <0.001 | * |
| --- | --- | --- | --- | --- | --- |
| Ireland | 7.96 | -3.03 | 20.20 | 0.139 |  |
| Lithuania | -0.80 | -15.84 | 16.91 | 0.913 |  |
| Norway | 4.81 | -10.47 | 22.70 | 0.511 |  |
| United Kingdom | -0.61 | -2.22 | 1.02 | 0.412 |  |
| ***Western Europe*** |  |  |  |  |  |
| Austria | -4.77 | -13.17 | 4.44 | 0.257 |  |
| France | 3.73 | -12.21 | 22.57 | 0.626 |  |
| Germany | 0.39 | -17.85 | 22.69 | 0.965 |  |
| Netherlands | 5.28 | -3.43 | 14.77 | 0.207 |  |
| Switzerland | 2.89 | -9.12 | 16.49 | 0.611 |  |
| ***Southern Europe*** |  |  |  |  |  |
| Croatia | -9.57 | -25.33 | 9.52 | 0.260 |  |
| Cyprus | -1.61 | -13.87 | 12.40 | 0.811 |  |
| Italy | -1.74 | -9.06 | 6.17 | 0.616 |  |
| Malta | 3.35 | -6.44 | 14.16 | 0.467 |  |
| Slovenia | 4.08 | -10.82 | 21.47 | 0.567 |  |
| Spain | 3.82 | -4.42 | 12.77 | 0.327 |  |
| ***Eastern Europe*** |  |  |  |  |  |
| Bulgaria | 0.55 | -6.80 | 8.49 | 0.871 |  |
| Czech Republic | -0.46 | -9.03 | 8.93 | 0.910 |  |
| Poland | -1.12 | -14.35 | 14.15 | 0.861 |  |
| ***Africa*** |  |  |  |  |  |
| Uganda | 13.81 | -10.22 | 44.29 | 0.244 |  |

AAPC, annual percentage change; CI, confidence interval; * p values less than 0.05.

NA, not available as it reported zero cases during the period and joinpoint regression could not be performed in such circumstances.

1. nasopharynx– young

| **Region** | **AAPC** | **Lower CI** | **Upper CI** | **p-value** | **Significant** |
| --- | --- | --- | --- | --- | --- |
| ***Asia*** |  |  |  |  |  |
| Bahrain | 1.61 | -13.47 | 19.31 | 0.846 |  |
| China | 0.51 | -1.40 | 2.46 | 0.555 |  |
| India | -0.36 | -8.18 | 8.12 | 0.921 |  |
| Israel | -2.99 | -6.84 | 1.01 | 0.121 |  |
| Japan | 4.98 | -3.70 | 14.44 | 0.230 |  |
| Korea | -0.85 | -5.17 | 3.67 | 0.670 |  |
| Kuwait | -5.47 | -19.28 | 10.70 | 0.435 |  |
| Philippines | -2.70 | -4.46 | -0.91 | 0.009 | * |
| Thailand | 0.71 | -3.08 | 4.65 | 0.683 |  |
| Turkey | 0.89 | -5.02 | 7.17 | 0.744 |  |
| ***Oceania*** |  |  |  |  |  |
| Australia | -0.17 | -3.28 | 3.04 | 0.904 |  |
| New Zealand | -3.27 | -9.94 | 3.88 | 0.361 |  |
| ***Northern America*** |  |  |  |  |  |
| Canada | -1.50 | -5.37 | 2.52 | 0.409 |  |
| USA | -0.66 | -2.82 | 1.55 | 0.509 |  |
| ***Southern America*** |  |  |  |  |  |
| Brazil | 2.51 | -9.40 | 15.97 | 0.656 |  |
| Chile | NA | NA | NA | NA | NA |
| Colombia | -0.02 | -16.56 | 19.79 | 0.998 |  |
| Ecuador | 5.00 | 2.77 | 7.29 | <0.001 | * |
| Martinique | 0.47 | -11.40 | 13.92 | 0.934 |  |
| ***Northern Europe*** |  |  |  |  |  |
| Denmark | 2.40 | -4.71 | 10.04 | 0.469 |  |
| Estonia | 0.32 | -14.26 | 17.38 | 0.964 |  |

| Iceland | -3.73 | -12.70 | 6.16 | 0.446 |  |
| --- | --- | --- | --- | --- | --- |
| Ireland | -8.48 | -22.62 | 8.24 | 0.301 |  |
| Lithuania | 14.79 | -2.63 | 35.33 | 0.089 |  |
| Norway | 21.62 | 3.93 | 42.31 | 0.021 | * |
| United Kingdom | -1.94 | -3.94 | 0.11 | 0.060 |  |
| ***Western Europe*** |  |  |  |  |  |
| Austria | -3.46 | -9.35 | 2.80 | 0.232 |  |
| France | 3.88 | -6.60 | 15.53 | 0.433 |  |
| Germany | -4.51 | -11.62 | 3.16 | 0.206 |  |
| Netherlands | 4.34 | -0.87 | 9.82 | 0.092 |  |
| Switzerland | -13.93 | -25.54 | -0.51 | 0.044 | * |
| ***Southern Europe*** |  |  |  |  |  |
| Croatia | 2.91 | -6.44 | 13.19 | 0.508 |  |
| Cyprus | -8.13 | -20.37 | 5.98 | 0.208 |  |
| Italy | 5.63 | -0.54 | 12.18 | 0.069 |  |
| Malta | 1.22 | -11.92 | 16.32 | 0.846 |  |
| Slovenia | 19.46 | 11.56 | 27.91 | <0.001 | * |
| Spain | 1.49 | -3.86 | 7.13 | 0.547 |  |
| ***Eastern Europe*** |  |  |  |  |  |
| Bulgaria | 3.36 | -2.13 | 9.17 | 0.200 |  |
| Czech Republic | -5.09 | -8.39 | -1.66 | 0.009 | * |
| Poland | -2.33 | -13.10 | 9.77 | 0.654 |  |
| ***Africa*** |  |  |  |  |  |
| Uganda | 5.17 | -5.52 | 17.06 | 0.310 |  |

AAPC, annual percentage change; CI, confidence interval; * p values less than 0.05.

NA, not available as it reported zero cases during the period and joinpoint regression could not be performed in such circumstances.

1. nasopharynx- old

| **Region** | **AAPC** | **Lower CI** | **Upper CI** | **p-value** | **Significant** |
| --- | --- | --- | --- | --- | --- |
| ***Asia*** |  |  |  |  |  |
| Bahrain | -16.51 | -24.57 | -7.58 | 0.003 | * |
| China | -2.79 | -4.67 | -0.88 | 0.010 | * |
| India | -8.85 | -23.00 | 7.90 | 0.282 |  |
| Israel | -4.16 | -9.29 | 1.27 | 0.114 |  |
| Japan | -0.87 | -4.68 | 3.10 | 0.621 |  |
| Korea | -2.58 | -5.92 | 0.89 | 0.124 |  |
| Kuwait | 12.47 | 0.21 | 26.24 | 0.047 | * |
| Philippines | -5.19 | -8.40 | -1.87 | 0.007 | * |
| Thailand | -3.51 | -7.23 | 0.37 | 0.070 |  |
| Turkey | -1.57 | -7.26 | 4.46 | 0.556 |  |
| ***Oceania*** |  |  |  |  |  |
| Australia | 0.42 | -2.75 | 3.69 | 0.771 |  |
| New Zealand | -1.55 | -8.78 | 6.26 | 0.650 |  |
| ***Northern America*** |  |  |  |  |  |
| Canada | -0.43 | -2.22 | 1.38 | 0.595 |  |
| USA | -1.80 | -4.40 | 0.87 | 0.157 |  |
| ***Southern America*** |  |  |  |  |  |
| Brazil | -13.74 | -21.84 | -4.80 | 0.009 | * |
| Chile | NA | NA | NA | NA | NA |
| Colombia | -7.63 | -20.48 | 7.29 | 0.256 |  |
| Ecuador | -2.14 | -9.76 | 6.11 | 0.555 |  |
| Martinique | 12.73 | -16.32 | 51.86 | 0.431 |  |
| ***Northern Europe*** |  |  |  |  |  |
| Denmark | 3.76 | -0.72 | 8.46 | 0.090 |  |
| Estonia | -7.85 | -22.81 | 10.01 | 0.318 |  |

| Iceland | -13.92 | -21.66 | -5.40 | 0.006 | * |
| --- | --- | --- | --- | --- | --- |
| Ireland | 7.52 | -4.77 | 21.40 | 0.205 |  |
| Lithuania | -3.97 | -13.69 | 6.84 | 0.407 |  |
| Norway | -8.08 | -17.79 | 2.77 | 0.120 |  |
| United Kingdom | -1.38 | -3.90 | 1.21 | 0.252 |  |
| ***Western Europe*** |  |  |  |  |  |
| Austria | -1.62 | -7.63 | 4.79 | 0.568 |  |
| France | -2.61 | -9.76 | 5.10 | 0.446 |  |
| Germany | 5.74 | -3.94 | 16.39 | 0.217 |  |
| Netherlands | -0.43 | -5.59 | 5.01 | 0.855 |  |
| Switzerland | 5.22 | -3.08 | 14.24 | 0.191 |  |
| ***Southern Europe*** |  |  |  |  |  |
| Croatia | -1.18 | -8.50 | 6.72 | 0.731 |  |
| Cyprus | 5.51 | -7.60 | 20.49 | 0.378 |  |
| Italy | -1.32 | -5.51 | 3.05 | 0.498 |  |
| Malta | -0.85 | -10.57 | 9.94 | 0.854 |  |
| Slovenia | 2.57 | -8.08 | 14.46 | 0.608 |  |
| Spain | -4.13 | -9.28 | 1.32 | 0.116 |  |
| ***Eastern Europe*** |  |  |  |  |  |
| Bulgaria | 2.12 | -3.41 | 7.97 | 0.411 |  |
| Czech Republic | 3.18 | -2.57 | 9.27 | 0.244 |  |
| Poland | -3.83 | -14.74 | 8.46 | 0.524 |  |
| ***Africa*** |  |  |  |  |  |
| Uganda | 0.15 | -13.35 | 15.75 | 0.982 |  |

AAPC, annual percentage change; CI, confidence interval; * p values less than 0.05.

NA, not available as it reported zero cases during the period and joinpoint regression could not be performed in such circumstances.

1. hypopharynx– both

| **Region** | **AAPC** | **Lower CI** | **Upper CI** | **p-value** | **Significant** |
| --- | --- | --- | --- | --- | --- |
| ***Asia*** |  |  |  |  |  |
| Bahrain | -0.69 | -10.67 | 10.40 | 0.883 |  |
| China | 10.81 | 7.06 | 14.70 | <0.001 | * |
| India | -1.60 | -4.99 | 1.92 | 0.321 |  |
| Israel | -1.59 | -11.32 | 9.21 | 0.732 |  |
| Japan | 5.19 | 1.71 | 8.80 | 0.009 | * |
| Korea | -0.55 | -3.72 | 2.71 | 0.702 |  |
| Kuwait | 8.77 | -6.02 | 25.90 | 0.260 |  |
| Philippines | -4.66 | -12.03 | 3.32 | 0.208 |  |
| Thailand | -5.19 | -9.33 | -0.86 | 0.025 | * |
| Turkey | -0.14 | -6.64 | 6.81 | 0.963 |  |
| ***Oceania*** |  |  |  |  |  |
| Australia | -1.12 | -2.53 | 0.32 | 0.111 |  |
| New Zealand | -3.55 | -8.86 | 2.07 | 0.179 |  |
| ***Northern America*** |  |  |  |  |  |
| Canada | -3.34 | -5.77 | -0.84 | 0.009 | * |
| USA | -2.45 | -3.18 | -1.73 | <0.001 | * |
| ***Southern America*** |  |  |  |  |  |
| Brazil | -11.22 | -18.69 | -3.06 | 0.014 | * |
| Chile | -4.27 | -17.13 | 10.60 | 0.554 |  |
| Colombia | -7.00 | -17.50 | 4.84 | 0.200 |  |
| Ecuador | 7.32 | -6.51 | 23.18 | 0.316 |  |
| Martinique | -7.33 | -14.62 | 0.58 | 0.064 |  |
| ***Northern Europe*** |  |  |  |  |  |
| Denmark | 1.98 | -0.01 | 4.00 | 0.051 |  |
| Estonia | 0.38 | -2.51 | 3.34 | 0.774 |  |

| Iceland | -9.71 | -20.07 | 2.00 | 0.089 |  |
| --- | --- | --- | --- | --- | --- |
| Ireland | -0.48 | -4.06 | 3.23 | 0.769 |  |
| Lithuania | 1.48 | -1.93 | 5.00 | 0.351 |  |
| Norway | -6.36 | -12.97 | 0.76 | 0.073 |  |
| United Kingdom | 2.12 | -0.18 | 4.49 | 0.067 |  |
| ***Western Europe*** |  |  |  |  |  |
| Austria | -2.15 | -3.32 | -0.97 | 0.003 | * |
| France | -3.28 | -4.95 | -1.59 | 0.002 | * |
| Germany | -2.64 | -5.65 | 0.46 | 0.085 |  |
| Netherlands | 0.28 | -1.93 | 2.54 | 0.779 |  |
| Switzerland | -5.19 | -8.77 | -1.45 | 0.013 | * |
| ***Southern Europe*** |  |  |  |  |  |
| Croatia | -4.76 | -6.41 | -3.08 | <0.001 | * |
| Cyprus | -11.52 | -21.69 | -0.03 | 0.050 | * |
| Italy | -0.69 | -5.84 | 4.74 | 0.772 |  |
| Malta | 20.45 | 6.96 | 35.64 | 0.007 | * |
| Slovenia | -1.66 | -6.63 | 3.58 | 0.479 |  |
| Spain | -3.71 | -6.86 | -0.47 | 0.030 | * |
| ***Eastern Europe*** |  |  |  |  |  |
| Bulgaria | 1.72 | -1.10 | 4.63 | 0.199 |  |
| Czech Republic | 0.08 | -2.67 | 2.91 | 0.949 |  |
| Poland | -1.31 | -8.14 | 6.03 | 0.683 |  |
| ***Africa*** |  |  |  |  |  |
| Uganda | -4.39 | -20.44 | 14.88 | 0.588 |  |

AAPC, annual percentage change; CI, confidence interval; * p values less than 0.05.

1. hypopharynx– male

| **Region** | **AAPC** | **Lower CI** | **Upper CI** | **p-value** | **Significant** |
| --- | --- | --- | --- | --- | --- |
| ***Asia*** |  |  |  |  |  |
| Bahrain | -12.25 | -19.32 | -4.57 | 0.007 | * |
| China | 11.56 | 8.29 | 14.93 | <0.001 | * |
| India | -1.91 | -5.71 | 2.04 | 0.293 |  |
| Israel | -0.80 | -13.35 | 13.57 | 0.895 |  |
| Japan | 5.13 | 1.54 | 8.84 | 0.011 | * |
| Korea | -1.15 | -4.40 | 2.22 | 0.450 |  |
| Kuwait | 22.23 | 7.62 | 38.82 | 0.002 | * |
| Philippines | 0.54 | -9.69 | 11.92 | 0.911 |  |
| Thailand | -4.99 | -9.37 | -0.40 | 0.037 | * |
| Turkey | -2.46 | -9.34 | 4.95 | 0.455 |  |
| ***Oceania*** |  |  |  |  |  |
| Australia | -1.83 | -3.28 | -0.35 | 0.022 | * |
| New Zealand | -3.99 | -10.12 | 2.56 | 0.193 |  |
| ***Northern America*** |  |  |  |  |  |
| Canada | -2.54 | -4.21 | -0.85 | 0.009 | * |
| USA | -2.80 | -4.28 | -1.30 | 0.003 | * |
| ***Southern America*** |  |  |  |  |  |
| Brazil | -11.11 | -19.13 | -2.31 | 0.021 | * |
| Chile | -4.94 | -17.18 | 9.12 | 0.472 |  |
| Colombia | -12.80 | -20.87 | -3.91 | 0.012 | * |
| Ecuador | NA | NA | NA | NA | NA |
| Martinique | -5.30 | -12.61 | 2.62 | 0.156 |  |
| ***Northern Europe*** |  |  |  |  |  |
| Denmark | 1.73 | -0.37 | 3.88 | 0.094 |  |
| Estonia | -0.46 | -3.69 | 2.87 | 0.754 |  |

| Iceland | -12.45 | -27.44 | 5.63 | 0.165 |  |
| --- | --- | --- | --- | --- | --- |
| Ireland | 0.85 | -3.58 | 5.48 | 0.675 |  |
| Lithuania | 1.11 | -2.57 | 4.93 | 0.511 |  |
| Norway | -6.20 | -12.22 | 0.23 | 0.057 |  |
| United Kingdom | 2.01 | -0.38 | 4.45 | 0.088 |  |
| ***Western Europe*** |  |  |  |  |  |
| Austria | -2.60 | -3.70 | -1.49 | 0.001 | * |
| France | -3.43 | -5.06 | -1.77 | 0.001 | * |
| Germany | -2.87 | -7.03 | 1.47 | 0.163 |  |
| Netherlands | -0.10 | -2.34 | 2.19 | 0.923 |  |
| Switzerland | -6.86 | -10.78 | -2.77 | 0.005 | * |
| ***Southern Europe*** |  |  |  |  |  |
| Croatia | -5.34 | -6.66 | -4.00 | <0.001 | * |
| Cyprus | -8.98 | -11.89 | -5.98 | <0.001 | * |
| Italy | 0.39 | -6.32 | 7.58 | 0.900 |  |
| Malta | 12.48 | -2.44 | 29.68 | 0.093 |  |
| Slovenia | -1.54 | -7.05 | 4.29 | 0.551 |  |
| Spain | -4.03 | -6.86 | -1.11 | 0.013 | * |
| ***Eastern Europe*** |  |  |  |  |  |
| Bulgaria | 1.51 | -1.85 | 4.98 | 0.335 |  |
| Czech Republic | -0.11 | -2.90 | 2.76 | 0.931 |  |
| Poland | -3.29 | -9.03 | 2.81 | 0.243 |  |
| ***Africa*** |  |  |  |  |  |
| Uganda | -15.61 | -25.91 | -3.88 | 0.017 | * |

AAPC, annual percentage change; CI, confidence interval; * p values less than 0.05.

NA, not available as it reported zero cases during the period and joinpoint regression could not be performed in such circumstances.

1. hypopharynx– female

| **Region** | **AAPC** | **Lower CI** | **Upper CI** | **p-value** | **Significant** |
| --- | --- | --- | --- | --- | --- |
| ***Asia*** |  |  |  |  |  |
| Bahrain | 29.34 | 24.50 | 34.37 | <0.001 | * |
| China | -13.43 | -29.10 | 5.71 | 0.157 |  |
| India | 1.77 | -4.65 | 8.63 | 0.597 |  |
| Israel | -14.67 | -32.80 | 8.35 | 0.164 |  |
| Japan | 5.69 | -0.21 | 11.95 | 0.057 |  |
| Korea | 2.03 | -3.66 | 8.05 | 0.443 |  |
| Kuwait | 10.32 | 6.50 | 14.27 | <0.001 | * |
| Philippines | -10.86 | -17.52 | -3.66 | 0.009 | * |
| Thailand | -6.53 | -23.11 | 13.63 | 0.448 |  |
| Turkey | 5.38 | -3.53 | 15.12 | 0.208 |  |
| ***Oceania*** |  |  |  |  |  |
| Australia | 2.77 | -1.00 | 6.68 | 0.130 |  |
| New Zealand | -5.21 | -20.97 | 13.70 | 0.517 |  |
| ***Northern America*** |  |  |  |  |  |
| Canada | -4.22 | -9.59 | 1.47 | 0.123 |  |
| USA | -0.88 | -5.43 | 3.89 | 0.676 |  |
| ***Southern America*** |  |  |  |  |  |
| Brazil | -9.15 | -15.51 | -2.31 | 0.010 | * |
| Chile | 7.32 | -6.51 | 23.18 | 0.316 |  |
| Colombia | -5.86 | -12.50 | 1.28 | 0.093 |  |
| Ecuador | 7.32 | -6.51 | 23.18 | 0.316 |  |
| Martinique | -23.61 | -30.18 | -16.43 | <0.001 | * |
| ***Northern Europe*** |  |  |  |  |  |
| Denmark | 2.65 | -1.42 | 6.89 | 0.174 |  |
| Estonia | 19.09 | 6.83 | 32.76 | 0.002 | * |

| Iceland | NA | NA | NA | NA | NA |
| --- | --- | --- | --- | --- | --- |
| Ireland | -5.14 | -10.86 | 0.94 | 0.086 |  |
| Lithuania | 15.61 | 3.25 | 29.45 | 0.018 | * |
| Norway | -7.31 | -21.25 | 9.11 | 0.315 |  |
| United Kingdom | 2.01 | -1.08 | 5.20 | 0.174 |  |
| ***Western Europe*** |  |  |  |  |  |
| Austria | 3.66 | -3.71 | 11.60 | 0.339 |  |
| France | -1.65 | -4.35 | 1.12 | 0.204 |  |
| Germany | -2.91 | -13.28 | 8.70 | 0.563 |  |
| Netherlands | 1.40 | -2.45 | 5.40 | 0.431 |  |
| Switzerland | 6.18 | -4.83 | 18.46 | 0.242 |  |
| ***Southern Europe*** |  |  |  |  |  |
| Croatia | -0.36 | -6.44 | 6.11 | 0.898 |  |
| Cyprus | -17.66 | -25.40 | -9.11 | 0.002 | * |
| Italy | -1.55 | -18.25 | 18.55 | 0.851 |  |
| Malta | 33.26 | 21.74 | 45.87 | <0.001 | * |
| Slovenia | -4.83 | -16.65 | 8.67 | 0.415 |  |
| Spain | 1.81 | -7.86 | 12.49 | 0.690 |  |
| ***Eastern Europe*** |  |  |  |  |  |
| Bulgaria | 5.15 | -5.53 | 17.03 | 0.311 |  |
| Czech Republic | 1.04 | -5.69 | 8.25 | 0.738 |  |
| Poland | 11.22 | -1.21 | 25.22 | 0.072 |  |
| ***Africa*** |  |  |  |  |  |
| Uganda | -7.21 | -17.87 | 4.84 | 0.230 |  |

AAPC, annual percentage change; CI, confidence interval; * p values less than 0.05.

NA, not available as it reported zero cases during the period and joinpoint regression could not be performed in such circumstances.

1. hypopharynx– young

| **Region** | **AAPC** | **Lower CI** | **Upper CI** | **p-value** | **Significant** |
| --- | --- | --- | --- | --- | --- |
| ***Asia*** |  |  |  |  |  |
| Bahrain | NA | NA | NA | NA | NA |
| China | 6.67 | -5.05 | 19.84 | 0.236 |  |
| India | -1.96 | -6.36 | 2.64 | 0.348 |  |
| Israel | 32.03 | 21.65 | 43.29 | <0.001 | * |
| Japan | 3.54 | -12.33 | 22.28 | 0.643 |  |
| Korea | -1.46 | -12.72 | 11.25 | 0.786 |  |
| Kuwait | 7.32 | -6.51 | 23.18 | 0.316 |  |
| Philippines | -16.95 | -24.44 | -8.72 | 0.002 | * |
| Thailand | 4.10 | -1.30 | 9.79 | 0.120 |  |
| Turkey | -1.08 | -10.72 | 9.60 | 0.813 |  |
| ***Oceania*** |  |  |  |  |  |
| Australia | -5.12 | -10.67 | 0.77 | 0.079 |  |
| New Zealand | 6.62 | -5.52 | 20.32 | 0.299 |  |
| ***Northern America*** |  |  |  |  |  |
| Canada | -0.49 | -6.01 | 5.35 | 0.848 |  |
| USA | -7.98 | -16.16 | 0.99 | 0.073 |  |
| ***Southern America*** |  |  |  |  |  |
| Brazil | 0.27 | -12.48 | 14.87 | 0.970 |  |
| Chile | NA | NA | NA | NA | NA |
| Colombia | -11.53 | -25.05 | 4.42 | 0.147 |  |
| Ecuador | NA | NA | NA | NA | NA |
| Martinique | -15.49 | -17.94 | -12.96 | <0.001 | * |
| ***Northern Europe*** |  |  |  |  |  |
| Denmark | -5.55 | -13.91 | 3.63 | 0.194 |  |
| Estonia | 2.01 | -5.66 | 10.30 | 0.573 |  |

| Iceland | 1.21 | -6.85 | 9.98 | 0.776 |  |
| --- | --- | --- | --- | --- | --- |
| Ireland | -4.73 | -20.00 | 13.46 | 0.541 |  |
| Lithuania | 3.90 | -2.42 | 10.64 | 0.197 |  |
| Norway | -8.37 | -22.08 | 7.74 | 0.290 |  |
| United Kingdom | -0.70 | -4.65 | 3.41 | 0.700 |  |
| ***Western Europe*** |  |  |  |  |  |
| Austria | -2.84 | -11.11 | 6.21 | 0.526 |  |
| France | -7.30 | -13.37 | -0.81 | 0.032 | * |
| Germany | -13.89 | -22.98 | -3.74 | 0.015 | * |
| Netherlands | -8.43 | -14.22 | -2.24 | 0.015 | * |
| Switzerland | -10.56 | -19.26 | -0.94 | 0.036 | * |
| ***Southern Europe*** |  |  |  |  |  |
| Croatia | -8.98 | -14.05 | -3.60 | 0.005 | * |
| Cyprus | -18.80 | -21.93 | -15.54 | <0.001 | * |
| Italy | -16.54 | -23.46 | -8.98 | 0.001 | * |
| Malta | 7.32 | -6.51 | 23.18 | 0.316 |  |
| Slovenia | -0.18 | -7.03 | 7.16 | 0.954 |  |
| Spain | -9.23 | -17.55 | -0.06 | 0.049 | * |
| ***Eastern Europe*** |  |  |  |  |  |
| Bulgaria | 2.01 | -3.78 | 8.16 | 0.454 |  |
| Czech Republic | -5.54 | -13.05 | 2.61 | 0.151 |  |
| Poland | 8.60 | -8.13 | 28.36 | 0.288 |  |
| ***Africa*** |  |  |  |  |  |
| Uganda | 14.06 | 10.37 | 17.87 | <0.001 | * |

AAPC, annual percentage change; CI, confidence interval; * p values less than 0.05.

NA, not available as it reported zero cases during the period and joinpoint regression could not be performed in such circumstances.

1. hypopharynx- old

| **Region** | **AAPC** | **Lower CI** | **Upper CI** | **p-value** | **Significant** |
| --- | --- | --- | --- | --- | --- |
| ***Asia*** |  |  |  |  |  |
| Bahrain | -1.10 | -3.98 | 1.88 | 0.465 |  |
| China | 13.97 | 9.07 | 19.09 | <0.001 | * |
| India | -2.14 | -5.82 | 1.68 | 0.229 |  |
| Israel | -9.95 | -23.90 | 6.56 | 0.189 |  |
| Japan | 5.60 | 2.26 | 9.05 | 0.005 | * |
| Korea | -1.30 | -5.21 | 2.77 | 0.477 |  |
| Kuwait | 12.50 | 4.20 | 21.46 | 0.003 | * |
| Philippines | -3.82 | -13.71 | 7.20 | 0.432 |  |
| Thailand | -3.70 | -14.84 | 8.90 | 0.548 |  |
| Turkey | 1.70 | -6.67 | 10.82 | 0.664 |  |
| ***Oceania*** |  |  |  |  |  |
| Australia | -1.21 | -2.79 | 0.38 | 0.117 |  |
| New Zealand | -4.32 | -10.87 | 2.71 | 0.189 |  |
| ***Northern America*** |  |  |  |  |  |
| Canada | -3.03 | -5.27 | -0.75 | 0.016 | * |
| USA | -1.85 | -2.97 | -0.71 | 0.006 | * |
| ***Southern America*** |  |  |  |  |  |
| Brazil | -10.74 | -17.66 | -3.24 | 0.012 | * |
| Chile | -4.27 | -17.13 | 10.60 | 0.554 |  |
| Colombia | -11.30 | -19.17 | -2.66 | 0.018 | * |
| Ecuador | NA | NA | NA | NA | NA |
| Martinique | -6.99 | -15.05 | 1.82 | 0.102 |  |
| ***Northern Europe*** |  |  |  |  |  |
| Denmark | 3.01 | 0.10 | 6.00 | 0.044 | * |
| Estonia | 1.34 | -2.56 | 5.40 | 0.456 |  |

| Iceland | -18.88 | -19.60 | -18.16 | <0.001 | * |
| --- | --- | --- | --- | --- | --- |
| Ireland | -0.20 | -5.39 | 5.28 | 0.934 |  |
| Lithuania | 1.06 | -2.47 | 4.71 | 0.513 |  |
| Norway | -6.10 | -12.60 | 0.88 | 0.077 |  |
| United Kingdom | 2.86 | 0.28 | 5.51 | 0.034 | * |
| ***Western Europe*** |  |  |  |  |  |
| Austria | -1.83 | -3.08 | -0.56 | 0.011 | * |
| France | -2.42 | -4.66 | -0.14 | 0.041 | * |
| Germany | -0.64 | -5.21 | 4.16 | 0.763 |  |
| Netherlands | 1.42 | -0.85 | 3.73 | 0.188 |  |
| Switzerland | -4.66 | -8.59 | -0.56 | 0.031 | * |
| ***Southern Europe*** |  |  |  |  |  |
| Croatia | -3.98 | -5.83 | -2.09 | 0.001 | * |
| Cyprus | -9.41 | -9.93 | -8.88 | <0.001 | * |
| Italy | 0.36 | -6.12 | 7.30 | 0.904 |  |
| Malta | 19.05 | 6.54 | 33.03 | 0.007 | * |
| Slovenia | -2.41 | -8.32 | 3.87 | 0.393 |  |
| Spain | -3.29 | -7.37 | 0.96 | 0.111 |  |
| ***Eastern Europe*** |  |  |  |  |  |
| Bulgaria | 2.00 | -1.18 | 5.28 | 0.187 |  |
| Czech Republic | 1.68 | -1.62 | 5.09 | 0.278 |  |
| Poland | -1.60 | -7.37 | 4.53 | 0.555 |  |
| ***Africa*** |  |  |  |  |  |
| Uganda | -8.19 | -23.69 | 10.46 | 0.318 |  |

AAPC, annual percentage change; CI, confidence interval; * p values less than 0.05.

NA, not available as it reported zero cases during the period and joinpoint regression could not be performed in such circumstances.
